# In silico simulations of occurrence of transcription factor binding sites in bacterial genomes

**DOI:** 10.1186/s12862-019-1381-8

**Published:** 2019-03-01

**Authors:** Jan Mrázek, Anna C. Karls

**Affiliations:** 1 Department of Microbiology, University of Georgia, Athens, GA USA; 2 Institute of Bioinformatics, University of Georgia, Athens, GA USA

**Keywords:** Gene regulatory networks, Evolution, Position-specific score matrix, Random sequence, Protein-DNA interactions

## Abstract

**Background:**

Interactions between transcription factors and their specific binding sites are a key component of regulation of gene expression. Until recently, it was generally assumed that most bacterial transcription factor binding sites are located at or near promoters. However, several recent works utilizing high-throughput technology to detect transcription factor binding sites in bacterial genomes found a large number of binding sites in unexpected locations, particularly inside genes, as opposed to known or expected promoter regions. While some of these intragenic binding sites likely have regulatory functions, an alternative scenario is that many of these binding sites arise by chance in the absence of selective constraints. The latter possibility was supported by in silico simulations for σ^54^ binding sites in *Salmonella*.

**Results:**

In this work, we extend these simulations to more than forty transcription factors from *E. coli* and other bacteria. The results suggest that binding sites for all analyzed transcription factors are likely to arise throughout the genome by random genetic drift and many transcription factor binding sites found in genomes may not have specific regulatory functions. In addition, when comparing observed and expected patterns of occurrence of binding sites in genomes, we observed distinct differences among different transcription factors.

**Conclusions:**

We speculate that transcription factor binding sites randomly occurring throughout the genome could be beneficial in promoting emergence of new regulatory interactions and thus facilitating evolution of gene regulatory networks.

**Electronic supplementary material:**

The online version of this article (10.1186/s12862-019-1381-8) contains supplementary material, which is available to authorized users.

## Background

Transcription factors regulate gene expression by binding to specific short DNA sequences in or near promoters and either activate or repress initiation of transcription. Most transcription factors interact with other components of the transcription initiation complex upon binding to DNA whereas some help initiate transcription by remodeling the DNA structure at the promoter, which subsequently allows the RNA polymerase to assemble at the promoter and initiate transcription (reviewed in [1]). Understandably, determining the DNA sequences recognized by individual transcription factors – the transcription factor binding sites – is of utmost interest with respect to understanding gene regulatory networks and connecting the transcription factor activity to the organism physiology in general. Generally, combinations of experimental and computational techniques are used for this purpose.

Recent genome-wide analyses of σ^54^ regulons in *E. coli* and *Salmonella* using chromatin immunoprecipitation coupled with deep sequencing (ChIP-seq) or microarray technology (ChIP-chip) yielded an unexpected result in detecting many σ^54^ binding sites outside known or potential promoters and mostly within genes [2–4]. The functions of these binding sites are subject of speculation; in particular, depending on location and orientation of these binding sites they could modulate gene expression by transcription interference or promoter competition [5, 6]. Bonocora and coworkers [3] proffered that many of the intragenic binding sites detected in *E. coli* were conserved in other bacteria and, therefore, likely to have functional relevance. On the other hand, we used a Monte Carlo approach and computer simulations to estimate how many σ^54^ binding sites are likely to occur by chance in the genome and found that many of the binding sites detected by the ChIP-chip experiments could be random occurrences arising in the absence of direct selective constraints on the binding sites and consequently may have no specific physiological function [2]. Instead, we proposed that these randomly occurring binding sites could play a significant role in the evolution of the regulatory networks. Comparisons of regulatory networks elucidated by reverse engineering from gene expression data among related bacteria showed that regulatory networks evolve rapidly by loss or gain of regulatory genes, as well as new regulatory interactions [7, 8]. Randomly occurring transcription factor binding sites may not necessarily have a significant negative effect on the organism fitness in most instances and could promote emergence of new regulatory interactions, thus contributing to the evolution of gene regulatory networks.

Widespread intragenic binding has also been reported for other transcription factors. Grainger et al. [9] used ChIP-chip to examine binding of the *E. coli* cAMP receptor protein (Crp) to the chromosomal DNA in vivo and reported that while the strongest binding sites were generally associated with known Crp-dependent promoters a large number of weaker binding sites were distributed throughout the chromosome. Intragenic binding was also reported for the pyrimidine catabolism master regulator RutR, apparently with no effect on the transcript levels [10]. More recently, extensive intragenic binding was detected in studies of genome-wide binding sites for 116 transcription factors in *E. coli* using genomic SELEX [11] and for 154 transcription factors in *Mycobacterium tuberculosis* using ChIP-seq [12]. The majority of the intragenic binding sites for these diverse transcription factors are not associated with demonstrated transcription start sites and have no known function. Fitzgerald and coworkers [13] investigated intragenic FliA-dependent promoters in *E. coli* and suggested that they could play evolutionary roles analogous to those we previously proposed for σ^54^ binding [2]. Considering the different mechanisms for regulating transcription and wide-ranging roles of transcription factors that bind to numerous intragenic sites, we have extended the simulation performed for σ^54^ binding sites to more than 40 additional transcription factors to investigate whether our results for σ^54^ apply generally to transcription factors and whether there are significant differences among different regulatory proteins.

## Results and discussion

### Accurate representation of the null hypothesis requires incorporating Markov dependencies and genome heterogeneity in the null model

The goal of the simulations was to assess occurrence of the motif (transcription factor binding site) sequences under the conditions of the null hypothesis, which assumes that the binding sites are not subject to direct selective constraints but might be influenced by other biases, such as biased codon and amino acid usage, dinucleotide usage biases, or local variance in GC content. We tested three different methods for generating random sequences (representing different null models) implemented in Genome Randomizer [14] (http://www.cmbl.uga.edu/software.html). The simplest model, ‘b’ for “homogeneous Bernoulli model”, reproduces only the overall GC content of the genome. This is the most commonly used model to assess whether a certain sequence feature is statistically unusual and it assumes that the probability of finding a particular nucleotide (A, C, G, or T) at a particular position in the sequence does not depend on the context or the location in the chromosome. In model ‘bb’, the genome annotation is used to divide the genome into segments consisting of individual protein-coding genes and intergenic regions; a random sequence is generated for each segment to mimic its nucleotide composition, and the randomized genome is reassembled from these segments. Consequently, the model reproduces the compositional heterogeneity of the sequence at the scale of individual genes (for example, AT-rich genes or intergenic segments in an otherwise GC-rich genome retain their lower GC content) as well as asymmetry between the coding (sense) and template (antisense) strand and between the leading and lagging strand with respect to the direction of replication (GC-skew) [15–17]. However, the ‘bb’ model still does not take into account the immediate context in terms of nearest-neighbor biases. These biases are reflected in the ‘m1c1’ model, which models each intergenic region as a first order Markov chain using the nucleotide alphabet and each gene as a first order Markov chain using the codon alphabet, where the next codon probability depends on the last base of the previous codon. Consequently, this model reproduces the sequence heterogeneity at the gene scale like the ‘bb’ model but also dinucleotide frequencies in each intergenic region and codon frequencies as well as frequencies of dinucleotides spanning adjacent codons in each gene.

**Fig. 1:**
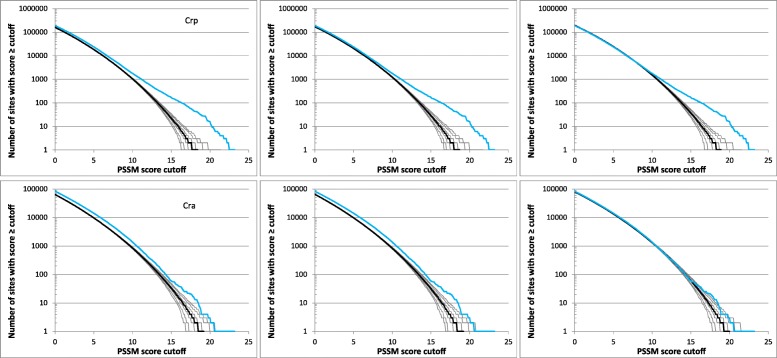
Reverse cumulative distributions of PSSM scores for Crp (top) and Cra (bottom) binding sites in the *E. coli* genome (blue) and random sequences (black). The ordinate displays the number of sites in the genome with scores greater or equal to the score cutoff indicated on the abscissa. The thick black line refers to the median value in 1000 random sequences and the thin lines to the 1st, 5th, 25th, 75th, 95th, and 99th percentiles, respectively. The random sequences were generated by the models ‘b’ (left), ‘bb’ (center), and ‘m1c1’ (right; see the text for details)

Figure 1 compares reverse cumulative distributions of PSSM (position-specific score matrix) scores in the *E. coli* genome and 1000 randomized genomes for two of the investigated transcription factors (*E. coli* Crp and Cra). Only the right tail of the distribution with scores > 0 is shown. Because the PSSM score equal to zero implies that the sequence at hand is equally likely to match a probabilistic model of the binding site derived from the training set as it is to match the probabilistic model of the background derived from the genome, it is reasonable to expect that scores close to zero are not affected by selection and the fit of the distribution for random sequences and actual genome sequence can be used as a measure of how accurately the null model captures biases unrelated to direct selection on the binding sites. The plots show that the model ‘b’ is the poorest match (the values for random sequences are systematically lower than those for the actual *E. coli* genome) and the model ‘bb’ is only slightly better, whereas the more complex ‘m1c1’ model provides a good match between the data and the model for scores close to zero with deviations occurring only for high scores which are likely to include physiologically important binding sites that are maintained by selection. A similar trend was observed for all other investigated transcription factors, with the ‘m1c1’ model generally providing a very good match to the observed values for scores close to zero. The most notable exception among the analyzed transcription factors is FlhDC, where the null model underestimates the number of sites even for PSSM scores close to zero (Additional file 1: Figure S1). Although we cannot reliably pinpoint the exact source of the discrepancy, one possible reason is that pentamers TATTT and CCNTT, which comprise the most conserved segments of the consensus FlhDC binding site, are more abundant in the *E. coli* genome than an average pentamer (TATTT has 12,114 copies compared to expected 9061 for an average pentamer and CCNTT has 37,725 copies compared to expected 36,243); because the null model takes into account only the biases at the level of codons and dinucleotides, systematic biases related to larger oligonucleotides, such as pentamers, could potentially lead to discrepancies such as the one observed for FlhDC. Using a higher order Markov chain for the null hypothesis could resolve such issues but it could also lead to overfitting. We therefore used the “m1c1” model in our analyses because it yields a good fit to the complete genome for scores close to zero for almost all transcription factors and invariably a better match than the simpler models (the complete set of the simulation results is available at http://www.cmbl.uga.edu/downloads/data_sets/2018/PSSM_simulations).

### Transcription factor binding sites are likely to arise by chance even in the absence of selection

**Fig. 2:**
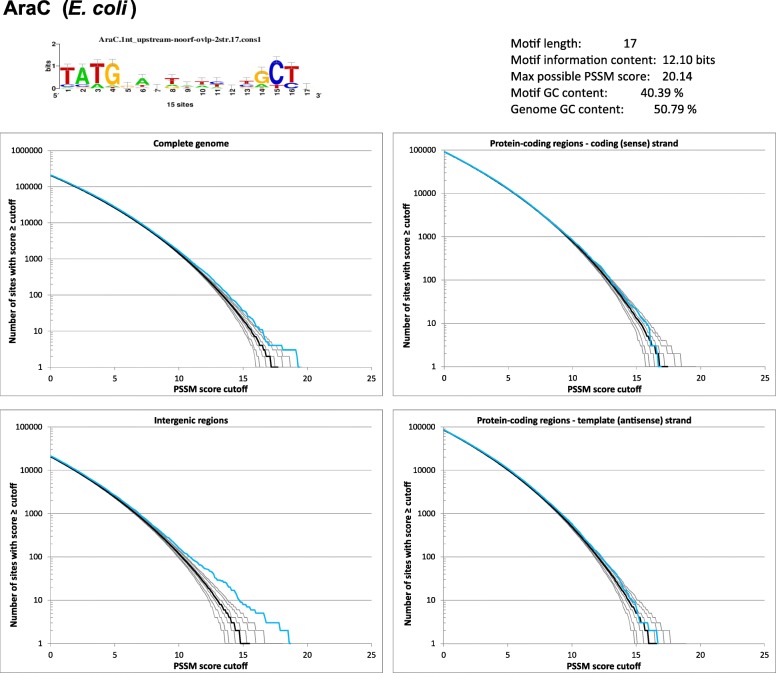
The ‘report card’ for *E. coli* AraC binding site. The information provided include the sequence logo for the site (top left), basic information about the motif (top right), and comparison of reverse cumulative distributions of PSSM scores in the genome and random sequences generated by the ‘m1c1’ model. The distributions are shown for all sites in the genome, restricted to intergenic sequences, and to protein-coding genes separately for coding and template strands. See Methods and legend to Fig. 1 for details

We summarized basic information about each analyzed transcription factor binding site and the main results of the simulations in a ‘report card’ such as the one shown in Fig. 2 for AraC. The complete set of report cards for all investigated transcription factors is presented in Additional file 1: Figure S1. The sequence logos were either downloaded from RegulonDB (http://regulondb.ccg.unam.mx/) [18] or generated by the WebLogo server at http://weblogo.berkeley.edu/logo.cgi [19]. The motif information content is derived from information entropy of each site in the alignment of motif sequences in the training set and equal to the sum of the height of all letters in the sequence logo [20]. Note that, whereas the motif information content is determined solely by the training set of high-confidence binding site sequences, the maximum PSSM score also depends on the sequence in which the search was performed, specifically on its GC content. That is because the PSSM scores are defined as log-ratios of target and background probabilities [21]. The report card also contains the key results of the simulations, including the distribution of PSSM scores in the complete genome, restricted to intergenic sequences, and protein-coding genes. The key observations from the AraC report card in Fig. 2 are the following: (i) For scores close to zero, the simulations match the observed values; (ii) the observed values begin to deviate from the simulations between the PSSM scores 10 and 15; not surprisingly, this is most apparent in the intergenic regions where promoters and the most physiologically important transcription factor binding sites are expected to reside; (iii) the observed values in protein-coding genes are close to those found in random sequences.

Another relevant observation is that although the observed values deviate from the simulations for high scores, the deviations are small. It is important to reiterate that the null model used to generate the random sequences reproduces the biases resulting from selection on codon usage and dinucleotide frequencies but not those reflecting potential selective constraints operating on longer oligonucleotides, including the transcription factor binding sites. The values in random sequences therefore represent an estimate of the number of binding sites that occur in the genome in the absence of selection and the small differences between the observed and expected values suggest that some of the binding sites in the genome have likely arisen by random genetic drift. It is important to note that the simulations can only provide an estimate of the number of sites expected to occur by chance; they cannot determine which specific binding sites are functional and which represent such random occurrences. Specifically, for simulations using the complete genome (the upper left panel in Fig. 2), there are only 4 hits in the *E. coli* genome with scores higher than the highest score expected to be found by chance (the median value of the highest scores found in the 1000 random sequences). We refer to this value as ΔN_genome_. Considering that the training set contained 15 AraC binding sites present in the *E. coli* genome and supported by evidence, this observation suggests that predicted binding sites in the *E. coli* genome with scores matching at least some of the verified binding sites are expected to arise from random genetic drift rather than from direct selection on the binding site. When the simulations are restricted to intergenic regions, the analogous value, ΔN_ig_, rises to 6 but this is still below the number of binding sites supported by evidence, suggesting that even intergenic regions, which represent a small fraction of a bacterial genome, may contain AraC binding sites that arise de novo by chance. Not surprisingly, ΔN_cod_ and ΔN_tem_, which refer to simulations using coding and template strands of genes, are 0 and 1, respectively, and the PSSM score distributions match closely the expected values; this is consistent with the expectation that protein-coding regions contain none or only few AraC binding sites maintained by selective constraints in addition to those that arise by chance.

Table 1 lists the ΔN values and other relevant information for all 43 transcription factors investigated in this work. Notably, only four of the 43 transcription factors have ΔN_genome_ ≥ 10 and all have more sites in the training set than ΔN_genome_, whereas three of the 43 transcription factors have ΔN_ig_ at least equal to the size of the training set, including Fnr in *R. sphaeroides* (but not Fnr in *E. coli*), LexA in *M. tuberculosis* (but not in *C. difficile* and just below the size of the training set in *E. coli*), and PurR with ΔN_ig_ = 21, the same as the size of the training set. These data suggest that the reasoning presented above for AraC is widely applicable to other transcription factors and that the genomes likely contain a number of transcription factor binding sites that arise by chance in absence of selection and probably do not have regulatory functions. We speculate that such spontaneous appearance of transcription factor binding sites could be important in providing sufficient plasticity of regulatory networks to allow adaptations to new conditions. ΔN_cod_ and ΔN_tem_ never exceed 3 (Table 1), suggesting that most binding sites found in genes, like those for σ^54^ in *E. coli* and *Salmonella* [2, 3], may be spurious occurrences resulting from random genetic drift and do not necessarily have a physiological function.

**Table 1:** Selected data about investigated transcription factor binding sites

Protein	Genome	Motif length	# of sites	Information content	ΔN_genome_	ΔN_ig_	ΔN_cod_	ΔN_tem_
AraC	*E. coli*	17	15	12.1	4	6	0	1
ArcA	*E. coli*	17	77	9.9	1	5	0	0
ArgP	*E. coli*	18	16	11.3	2	3	2	0
ArgR	*E. coli*	18	29	15.7	8	10	0	0
CpxR	*E. coli*	14	58	9.0	2	3	1	0
Cra	*E. coli*	16	42	14.7	2	14	0	0
Crp	*E. coli*	22	260	11.3	28	55	1	3
CsgD	*E. coli*	17	24	8.4	1	2	0	1
CytR	*E. coli*	18	18	11.3	1	2	1	0
DnaA	*E. coli*	11	14	13.0	0	8	0	0
FadR	*E. coli*	18	16	15.6	5	12	0	2
Fis	*E. coli*	15	214	6.9	0	3	0	0
FlhDC	*E. coli*	16	16	12.8	2	13	1	0
Fnr	*E. coli*	14	84	11.1	4	10	0	0
Fnr	*R. sphaeroides*	14	27	17.1	18	38	0	1
Fur	*E. coli*	18	48	15.8	9	27	1	1
GadW	*E. coli*	20	17	14.5	1	5	0	0
GadX	*E. coli*	21	24	11.3	2	5	0	0
GalR	*E. coli*	15	12	16.3	3	9	0	2
GlpR	*E. coli*	19	17	15.3	3	5	1	0
H-NS	*E. coli*	13	48	8.2	0	0	0	1
IHF	*E. coli*	13	95	8.4	0	4	0	3
LexA	*C. difficile*	16	17	15.4	1	0	0	0
LexA	*E. coli*	20	40	17.9	24	36	0	1
LexA	*M. tuberculosis*	18	23	22.5	21	24	2	3
Lrp	*E. coli*	15	80	7.1	0	3	0	1
MalT	*E. coli*	10	15	12.0	0	3	0	0
MarA	*E. coli*	19	23	12.4	2	6	0	0
MetJ	*E. coli*	16	15	14.1	2	14	0	2
Nac	*E. coli*	17	14	10.6	1	2	0	0
NagC	*E. coli*	22	20	19.9	7	10	1	1
NarL	*E. coli*	17	67	7.8	0	5	0	0
NsrR	*E. coli*	14	39	12.2	0	1	0	0
NtrC	*E. coli*	18	25	14.4	5	9	0	0
OmpR	*E. coli*	19	20	13.5	3	4	0	1
OxyR	*E. coli*	20	34	11.2	0	2	1	1
PhoB	*E. coli*	22	24	13.4	4	9	1	0
PhoP	*E. coli*	17	33	12.0	4	4	2	1
PurR	*E. coli*	16	21	20.5	8	21	1	0
Rob	*E. coli*	17	13	14.2	3	7	0	0
σ^54^	*S. enterica*	18	53	16.9	3	4	1	0
SoxS	*E. coli*	18	27	12.5	3	7	0	0
TyrR	*E. coli*	17	19	15.8	6	7	0	0

The DNA-binding proteins investigated in this work are listed together with the length of the motif in nucleotides, number of sites in the training set, and motif information content (in bits). ΔN_genome_ is the number of sites found in the genome that have higher PSSM score than the highest score expected to be found in the random sequence (median value among the 1000 simulations). ΔN_ig_ is the analogous value when the search is restricted to intergenic sequences and ΔN_cod_ and ΔN_tem_ are the analogous values for the search restricted to codon and template strands of protein-coding genes, respectively

### Similarities and differences among transcription factors

**Fig. 3:**
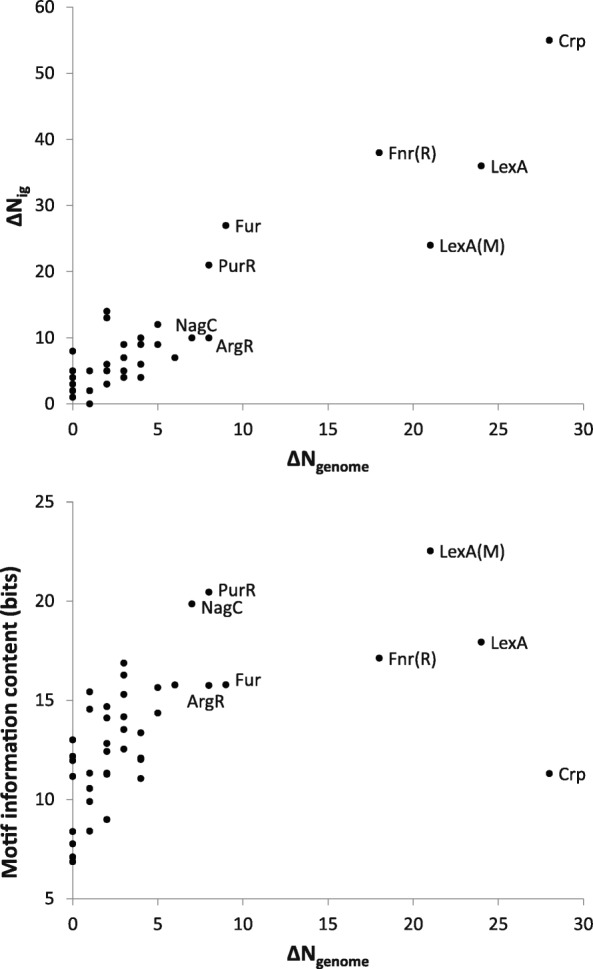
Relationship between ΔN_genome_ and ΔN_ig_ (top) and between ΔN_genome_ and the motif information content (bottom). Outliers are labeled. For transcription factors from other bacteria than *E. coli* the species is signified by the letter in parentheses, M for *M. tuberculosis* and R for *R. sphaeroides*

Transcription factors that stand out in terms of high ΔN are identified in Fig. 3. Not surprisingly, ΔN_genome_ and ΔN_ig_ exhibit a strong correlation, consistent with the notion that the transcription factor binding sites that are maintained by selection are predominantly located in intergenic regions. The most significant outlier is the catabolic repression protein Crp, followed by LexA in *E. coli* and *M. tuberculosis* (but not LexA in *C. difficile*), Fnr in *R. sphaeroides* (but not Fnr in *E. coli*; we discuss the differences among the same transcription factors from different species in the following section), and to lesser extent by *E. coli* Fur and PurR.

Also shown in Fig. 3 is the relationship between the ΔN_genome_ and the motif information content. The information content of the motif can serve as a surrogate measure of the transcription factor binding specificity (caveat: this assumes that the training set is accurate and representative, which may not be the case and the quality of the training set may differ for different transcription factors). Functionally important binding sites that are maintained by selection are therefore more likely to stand out from the random background if the sequence motif has a high information content, which in turn can lead to high ΔN values. As expected, the transcription factor binding sites with high ΔN_genome_ also tend to have high motif information contents with the notable exception of Crp, which has the highest ΔN_genome_ and ΔN_ig_ among all transcription factor binding sites investigated in this work but below average motif information content.

**Fig. 4:**
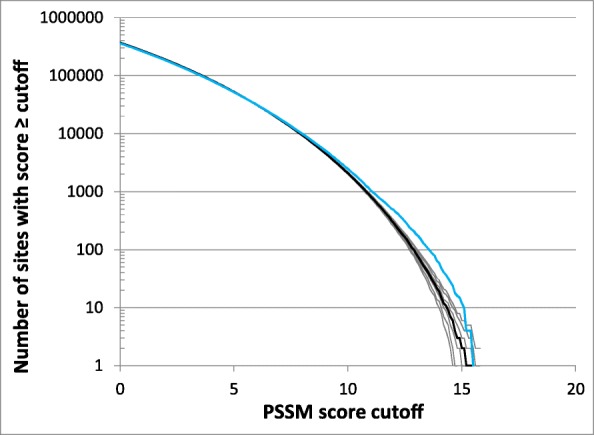
Reverse cumulative distributions of PSSM scores for IHF binding sites in the *E. coli* genome (blue) and random sequences (black). See legend to Fig. 1

What makes Crp unusual? Being a global regulator can be a reason for high ΔN_genome_ and ΔN_ig_. The Crp binding site training set in RegulonDB contains 260 sequences supported by experimental evidence, by far the largest among the transcription factors analyzed in this work (Table 1). According to other sources, Crp in *E. coli* regulates at least 190 genes [11, 22]. The high number of Crp binding sites that have regulatory functions and are subject to selective constraints leads to an excess of sites with high PSSM scores (Fig. 1 and Additional file 1: Figure S1). Crp is also functionally distinct from other transcription factors in our list that are considered global regulators, notably IHF, Fis, Lrp, and H-NS which all have ΔN_genome_ = 0 and ΔN_ig_ ≤ 4 (Table 1, Fig. 4, and Additional file 1: Figure S1). In addition to their role in regulation of transcription, IHF, Fis, Lrp, and H-NS are also nucleoid-associated proteins, which contribute to maintenance of bacterial nucleoid structure and their regulatory function is related to their roles in remodeling the local and global structure of the nucleoid [23]. They have relatively low binding specificity, which is reflected in low information contents of their binding sites (Table 1). Unfortunately, we were not able to find a usable training set to include another important nucleoid-associated protein HU in our simulations, which is in part because it interacts with DNA in a different manner than typical transcription factors. The HU binding is thought to have a low sequence specificity and its binding is largely determined by DNA structure and supercoiling [24].

### Comparison of LexA and Fnr binding sites in distantly related bacteria

**Fig. 5:**
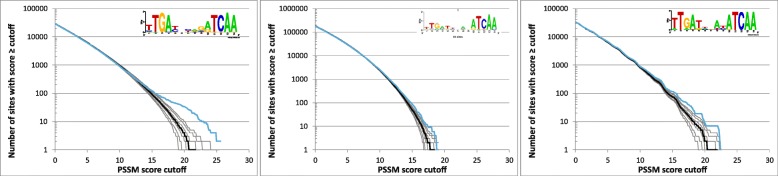
Reverse cumulative distributions of PSSM scores for the Fnr binding site in *R. sphaeroides* (left), in *E. coli* using the complete training set (center), and in *E. coli* but using reduced training set of top 20 binding sites in the training set (right). The inserts show the sequence logos representing the training sets used to construct the PSSM

While our results center on transcription factors from *E. coli* (a γ-proteobacterium) for which we could obtain training sets from RegulonDB, we also included transcription factor binding sites from other bacteria, namely the Fnr binding site from the α-proteobacterium *Rhodobacter sphaeroides* [25] and LexA binding sites from *Mycobacterium tuberculosis* (phylum Actinobacteria) [26] and *Clostridium difficile* (phylum Firmicutes) [27]. The reverse cumulative distributions of PSSM scores for the Fnr binding sites in *E. coli* and *R*. *sphaeroides* are markedly different despite similar consensus sequence (Fig. 5; see also Additional file 1: Figure S1). The main obvious difference in the sequence logos that represent the training sets used to construct the PSSM is that the Fnr binding site in *R. sphaeroides* has higher information content. This difference is also reflected in higher ΔN_genome_ and ΔN_ig_ in *R. sphaeroides* (Table 1). This could be a result of how the training sets were assembled; the *E. coli* training set was downloaded from RegulonDB, which compiles information from multiple sources and the evidence in support of an individual binding site may originate from studies relying on different methodologies, whereas the training set for *R. sphaeroides* contains Fnr binding sites identified in a single study and supported by ChIP-chip data [25]. Moreover, the *E. coli* training set contains 84 binding sites compared to 27 in the *R. sphaeroides* Fnr training set.

To investigate whether the differences in the motif information contents and the size of the training set could cause the differences in the PSSM score distributions, we reduced the *E. coli* training set to 20 sites most similar to the consensus and repeated the simulations (Fig. 5 and Additional file 2: Figure S2). Although the information content of the sequence motif generated from the reduced training set increased to 19.6 bits compared to 11.1 bits when using the complete training set of 84 sites and 17.1 bits for the Fnr binding site from *R. sphaeroides* (Table 1), the resulting distribution of PSSM scores resembles that of the *E. coli* Fnr with the full training set and lacks the ‘bump’ in the tail seen in the score distribution for Fnr in *R. sphaeroides*. We also performed cross-species simulations, i.e., searching for *R. sphaeroides* Fnr motif in the *E. coli* genome and vice versa, and both resulted in PSSM score distributions more similar to those shown in Fig. 5 for *E. coli* than those for *R. sphaeroides* (Additional file 2: Figure S2). We therefore conclude that the differences in our results for Fnr in *E. coli* and *R. sphaeroides* are not due to the differences in the training sets or the motif information contents.

One factor that could contribute to the difference between Fnr binding sites in *E. coli* and *R. sphaeroides* is the level of contrast between the GC content of the binding set and that of the genome; the *R. sphaeroides* genome is GC-rich (69% GC) and the *E. coli* genome is GC-neutral (51% GC), whereas the Fnr binding site is AT-rich in both bacteria. However, the difference in our results for Fnr in *E. coli* and *R. sphaeroides* may also arise from physiological differences between the two bacteria, specifically the number of genes regulated by Fnr. In most bacteria, the core Fnr regulon includes genes involved in response to O_2_ deprivation but in *R. sphaeroides* it also regulates photosynthetic genes, which are not present in *E. coli* and most other bacteria [25].

**Fig. 6:**
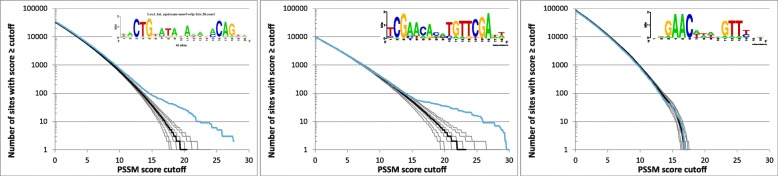
Reverse cumulative distributions of PSSM scores for the LexA binding site in *E. coli* (left), *M. tuberculosis* (center), and *C. difficile* (right). The inserts show the sequence logos representing the training sets used to construct the PSSM

Unlike Fnr, which has similar binding site consensus in *E. coli* and *R. sphaeroides*, the LexA binding site motifs differ significantly among the three compared genomes, *E. coli*, *M. tuberculosis*, and *C. difficile* (Fig. 6). The PSSM score distributions show clear excess of high-scoring predicted LexA binding sites in *E. coli* and *M. tuberculosis* compared to random sequences but little differences between the observed and simulated PSSM score distributions in *C. difficile* (Fig. 6 and Additional file 1: Figure S1). It is intriguing to speculate that the divergences in the PSSM score distributions relate to variations in the LexA roles among different species, possibly in combination with differences in the genome GC contents. However, the detailed roles of LexA in different bacteria are not well understood and there is no obvious connection to the observed differences in PSSM score distributions. Distinctions in the LexA roles in *C. difficile* include positive regulation of sporulation, which is a cellular process that is not exhibited by either *E. coli* or *M. tuberculosis*, and negative regulation of essential housekeeping genes, *rpoB* and *rplR*, which has not been reported for the LexA regulon of *E. coli* or *M. tuberculosis* and is likely to have pleiotropic effects on the cell [26–29]. With respect to GC content, the binding site motifs are AT-rich in all three genomes (32% GC in *C. difficile*, 36% in *E. coli*, and 41% in *M. tuberculosis*), whereas genome GC contents are dramatically different, ranging from 29% in *C. difficile*, to 51% in *E. coli*, and 66% in *M. tuberculosis*. Interestingly, the LexA binding sites themselves are more similar between *C. difficile* and *M. tuberculosis* with both exhibiting the consensus pattern GAAC(N)_4_GTT, than any of them is to *E. coli*, which has a consensus GTG(N)_10_CAG (Fig. 6). Cross-species searches (for example, searching for *C. difficile* LexA binding site in *M. tuberculosis* genome) resulted in PSSM score distributions similar to those found in randomized genomes (Additional file 2: Figure S2), which is not surprising considering the differences among the binding site consensus sequences (Fig. 6).

### Transcription factor binding sites in protein coding regions

In our previous work [2], we noted a mild suppression of high-scoring σ^54^ binding sites in the template strand of protein coding genes (but not the coding strand) and we hypothesized that the binding sites in the template strand could be detrimental due to conflicts of RNA polymerases progressing in the opposite directions if the σ^54^ binding sites in the template strand resulted in transcription initiation. It should be noted that unlike any of the transcription factors analyzed in this study, σ^54^ binds DNA as part of the RNA polymerase holoenzyme and each binding site has the potential to be an active promoter. In contrast to σ-factors, transcription factor binding is not sufficient to initiate transcription in the absence of a proximal promoter and randomly occurring transcription factor binding sites are therefore less likely to result in potentially detrimental transcriptional interference. Inspection of data in Additional file 1: Figure S1 suggests that although such suppression of transcription factor binding sites in protein-coding genes is not common, some of the investigated transcription factor binding sites have fewer high-scoring hits in genes and particularly in the template strand, including ArcA, MarA, OmpR, and PurR. On the other hand, none of the investigated transcription factors have significantly more high-scoring binding sites in genes than expected (Table 1 and Additional file 1: Figure S1).

### Potential implications for evolution of regulatory networks

Our results are consistent with a scenario in which the regulatory DNA-binding proteins have ‘just the right’ level of specificity for their respective binding sites that facilitates high-affinity binding to physiologically important promoters (sites with the highest PSSM scores tend to be located in intergenic regions) while also allowing binding to randomly occurring sites in the genome. This allows for emergence of new regulatory interactions, which, when beneficial, could become fixed in the population. This scenario is also consistent with earlier works showing that regulatory networks evolve rapidly by gain and loss of regulatory proteins as well as specific regulatory interactions determined by the interface between the DNA-binding domain of the regulatory protein and its binding sites in the DNA [7, 8] and may explain the widespread binding of transcription factors to sites located in unexpected places, including inside protein-coding regions [2, 11–13]. Some of such randomly occurring transcription factor binding sites may subsequently be incorporated into regulatory networks, which may include standard transcriptional regulation as well as non-canonical mechanisms of transcriptional or posttranscriptional control, such as synthesis of small regulatory RNAs or transcriptional interference [5, 6], regulation of adjacent operons [30], or act as transcriptional regulators for previously unrecognized protein-coding genes [31].

A caveat in this interpretation of the results relates to the use of PSSM scores as a surrogate measure of binding affinity to a particular site in the DNA. The PSSM model is widely used for computational prediction of transcription factor binding sites but it cannot capture cooperative effects among multiple binding sites or the influence of supercoiling levels, DNA bending, and/or other variations in DNA structure on the DNA-protein interaction [32–35]. In this regard, our comparison of PSSM scores with ChIP-chip signal intensities for σ^54^ binding sites showed a significant but noisy correlation, suggesting that the PSSM scores offer a meaningful quantitative estimate of the binding affinity but with a limited accuracy, which probably results from factors that cannot be captured in the binding site motif, such as wider sequence context or structure of the surrounding DNA segment [36]. Moreover, despite its simplicity, the PSSM method is still the most commonly used technique to predict transcription factor binding sites and attempts to add more sophisticated improvements did not result in better accuracy [36]. The main limitation, however, is related to the quality and size of the training set, which varies widely among the different transcription factors analyzed in this work. Despite these limitations, we believe that our main conclusion, that many of the transcription factor binding sites identified by recent analyses of genome-wide DNA binding (ChIP-chip, ChIP-seq, and genomic SELEX) may occur randomly in the absence of selective constraints, is justified for the following reasons: (i) The results are consistent for all the analyzed transcription factors regardless of the size of the training set; in addition, restricting the training set to a subset of most conserved sites does not qualitatively change the results. (ii) For low PSSM score cutoffs, the observed values match the expected values from the simulations; that was also true for cross-species searches with LexA binding sites where selection is not expected to play a role. (iii) Finally, the random emergence of binding sites may be required to facilitate the rapid evolution of regulatory networks, which was suggested by independent and very different methodology [7, 8].

## Conclusions

To address potential roles of transcription factor binding sites found outside of known or expected promoters [2–4, 9–13], we performed a series of in silico simulations for 43 transcription factors with the goal to estimate how many of their binding sites are likely to occur in the genome by chance, that is, in the absence of selective constraints operating directly on the binding sites. Using a null model that reflects the codon usage and nearest neighbor biases inherent in the genome, we found that for all transcription factors included in our study, the excess of predicted binding sites in the natural genome relative to the randomized genomes was always smaller than the number of known binding sites for the given transcription factor. Moreover, the numbers of predicted binding sites in the natural genome were often very similar to those in the randomized genomes. We interpret this result as an indication that a significant fraction of the transcription factor binding sites found in a genome could arise from random genetic drift without having a physiological function in the cell. We speculate that such randomly occurring transcription factor binding sites could play an important role in evolution of gene regulatory networks by providing opportunities for emergence of new regulatory interaction. This scenario is consistent with the observation that regulatory networks evolve rapidly by loss or gain of regulatory genes, as well as new regulatory interactions [7, 8].

## Methods

### DNA sequences and motif training sets

The genomic DNA sequence including annotation in the GenBank format were downloaded from the NCBI database (https://www.ncbi.nlm.nih.gov/) for *E. coli* K12 substrain MG1655 (accession number NC_000913), *Salmonella enterica* serovar Typhimurium strain 14028S (NC_016856), *Mycobacterium tuberculosis* H37Rv (AL123456), *Clostridium difficile* R20291 (FN545816), and chromosome 1 of *Rhodobacter sphaeroides* 2.4.1 (NC_007493). For the *E. coli* transcription factor binding sites, the training sets were obtained from RegulonDB (http://regulondb.ccg.unam.mx/) [18] directly in the form of the frequency matrices whereas for the transcription factor binding sites from the other genomes the frequency matrices were constructed from collections of known binding sites obtained from original literature. The sample of H-NS binding sites was extracted from RegulonDB flat file (http://regulondb.ccg.unam.mx/menu/download/datasets/files/PSSMSet.txt). The data files used in the simulations are available for download at http://www.cmbl.uga.edu/downloads/data_sets/2018/PSSM_simulations.

### Motif search

The standard PSSM method implemented in the Motif Locator program previously developed in our laboratory [37] was used to assign scores to potential binding sites. In brief, frequency matrix {*N*_*a*, *i*_}, consisting of counts of the letter (nucleotide) *a* at motif position *i*, is constructed from the training set of known motif sequences. Pseudocounts (an arbitrary small number) are added to the values *N*_*a*, *i*_ to account for the uncertainty resulting from the limited size of the training set. The frequency matrix {*N*_*a*, *i*_} is converted to probability matrix {*p*_*a*, *i*_}, where *p*_*a*, *i*_ is a probability of finding the letter *a* at position *i* of the motif. The position-specific score matrix (PSSM) is subsequently defined as *s*_*a*, *i*_ = log(*p*_*a*, *i*_/*q*_*a*_); *q*_*a*_ are the background probabilities, that is probabilities of finding the letter *a* at any position in the genome, which reflect the genome GC content. Assuming the motif has the length *L* nucleotides, any *L*-mer can be assigned a score $S=\sum \limits_{i=1}^L{s}_{a_i,i}$, where *a*_*i*_ is the nucleotide at position *i* of the *L*-mer at hand. The score *S* is referred to as PSSM score and reflects the ratio of the probability that the *L*-mer matches a randomly selected sequence from the probabilistic model of the motif represented by the probability matrix and the probability that it matches a randomly selected sequence from the probabilistic model of the background represented by the background probabilities. For more details, see, for example, ref. [21]. All overlapping *L*-mers in both strands of the genome are subsequently assigned PSSM scores; for the purposes of this work, the number of *L*-mers with scores greater or equal to a selected score cutoff is recorded. In-house software was used to mask protein-coding segments (the CDS features in the GenBank file Features Table) to evaluate PSSM scores in intergenic regions and to extract annotated protein-coding sequence to evaluate scores in protein-coding genes.

### Monte Carlo simulations

Monte Carlo approach is used to estimate the distribution of the numbers of L-mers with scores greater or equal to a selected score cutoff in the absence of selective constraints operating on the motif. One thousand random sequences were generated by one of the three stochastic models, ‘b’, ‘bb’, and ‘m1c1’, implemented in the Genome Randomizer program previously developed in our laboratory [14] (http://www.cmbl.uga.edu/software.html). The PSSM method was used to find the number of *L*-mers scoring above each selected cutoff in each of the random sequences in exactly the same manner as it was used for the actual genome sequence. The complete set of computer programs used to perform the simulations is available for download at http://www.cmbl.uga.edu/downloads/data_sets/2018/PSSM_simulations.

## Additional files

Additional file 1: **Figure S1.** Complete set of ‘report cards’ for transcription factor binding sites investigaed in this work. (PDF 3337 kb)

Additional file 2: **Figure S2.** ‘Report cards’ for additional tests including cross-species searches. (PDF 849 kb)

## Abbreviations

cAMP: Cyclic adenosine monophosphate
ChIP: Chromatin immunoprecipitation
PSSM: Position-specific score matrix
SELEX: Systematic evolution of ligands by exponential enrichment
