# Supplementary material for: In silico simulations of occurrence of transcription factor binding sites in bacterial genomes
Source: BMC Evol Biol. 2019 Mar 1;19:67. doi: 10.1186/s12862-019-1381-8 (PMC6397444; doi:10.1186/s12862-019-1381-8)

AraC (*E. coli*)

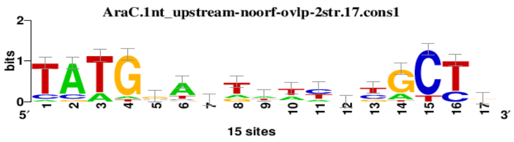

Motif length: 17
Motif information content: 12.10 bits
Max possible PSSM score: 20.14
Motif GC content: 40.39 %
Genome GC content: 50.79 %

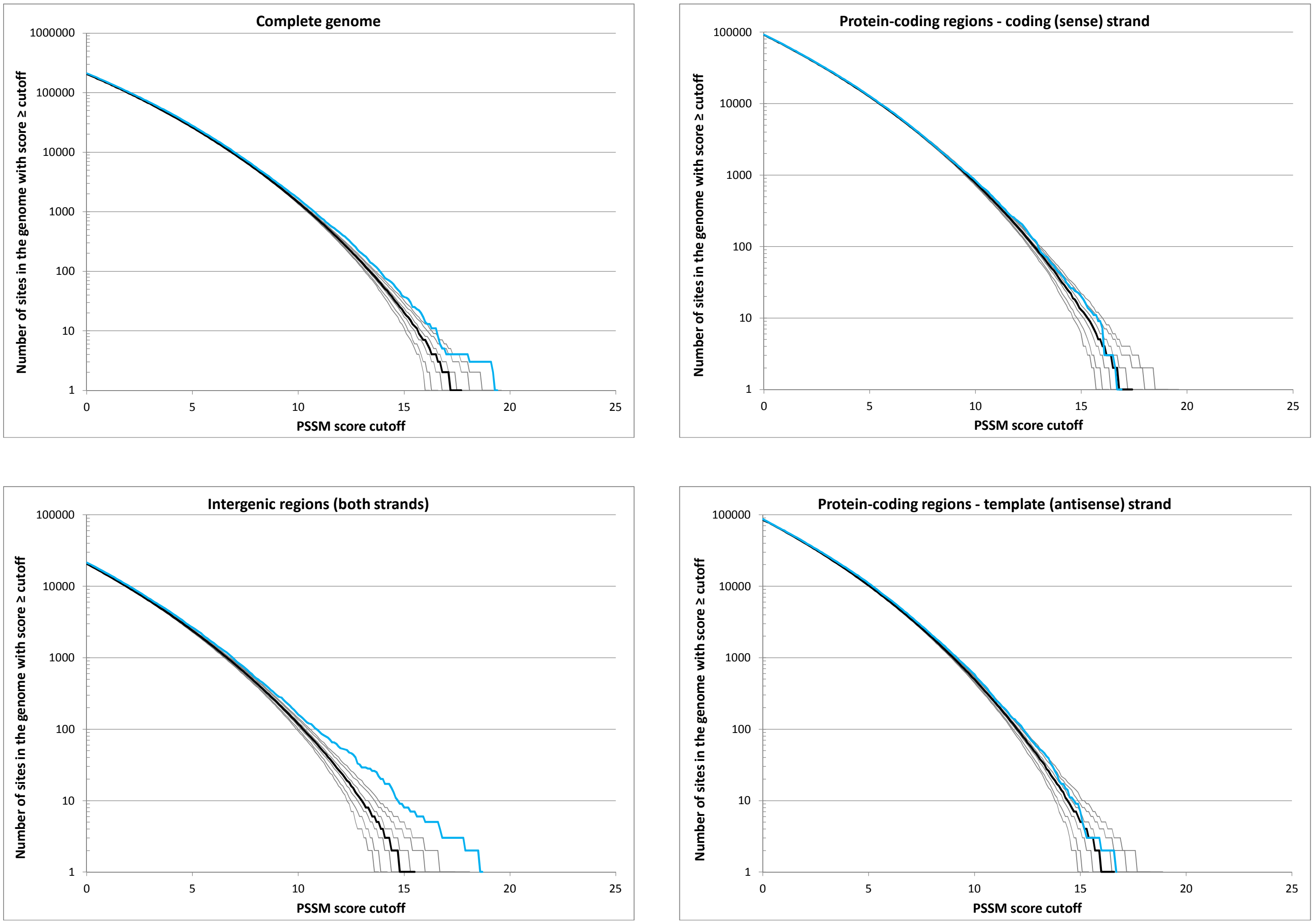

ArcA (*E. coli*)

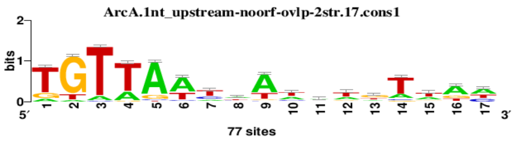

Motif length: 17
Motif information content: 9.89 bits
Max possible PSSM score: 20.27
Motif GC content: 24.37 %
Genome GC content: 50.79 %

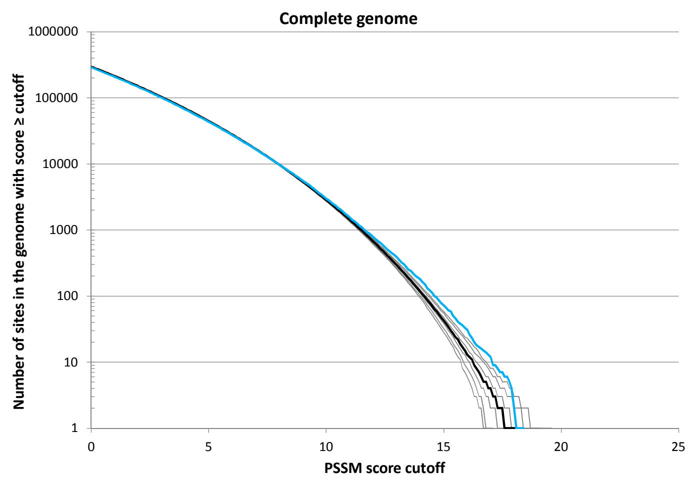

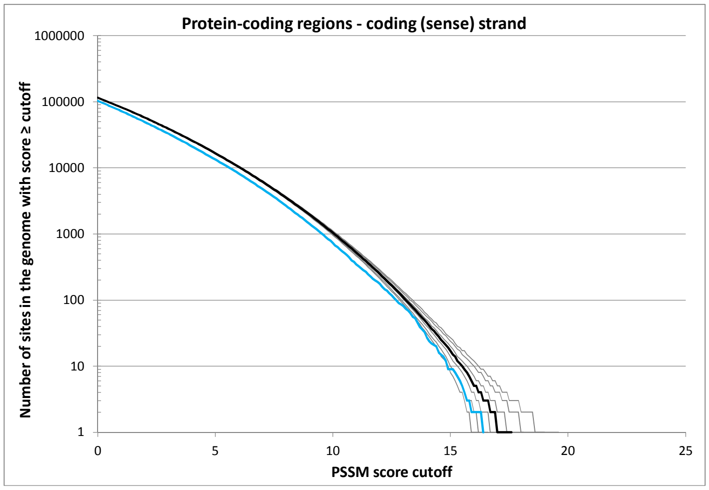

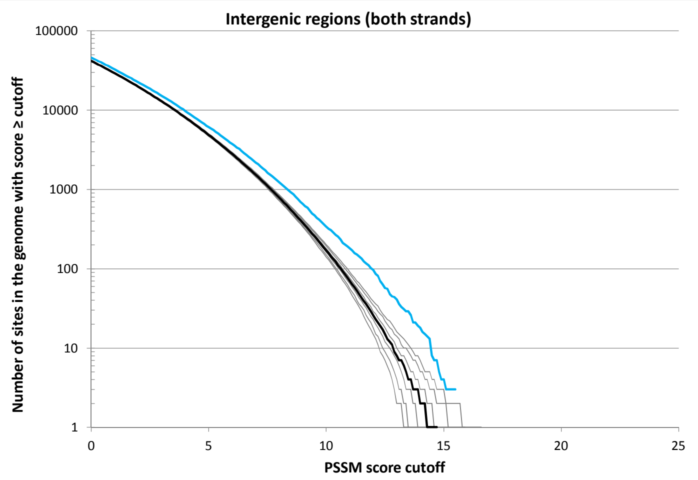

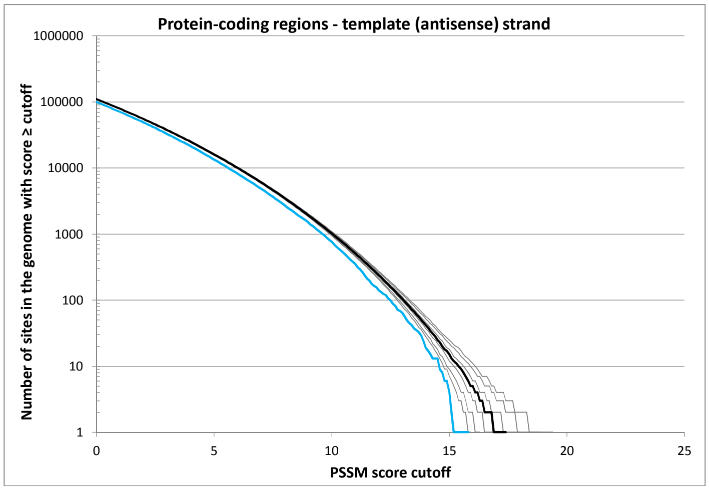

ArgP (*E. coli*)

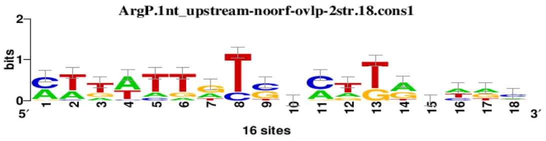

Motif length: 18
Motif information content: 11.27 bits
Max possible PSSM score: 18.58
Motif GC content: 34.72 %
Genome GC content: 50.79 %

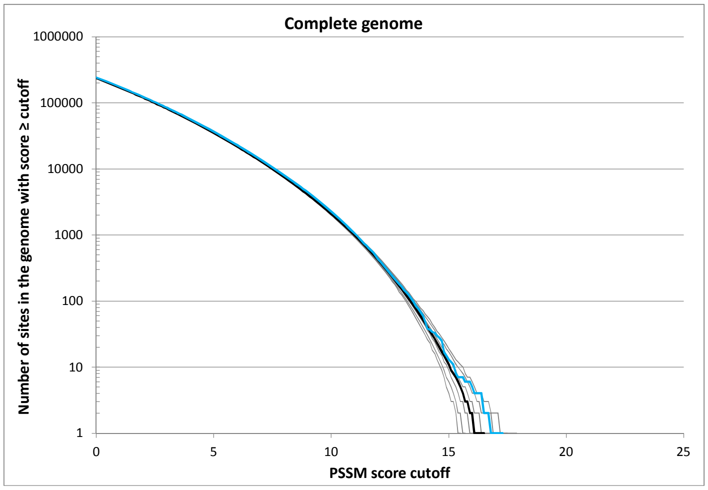

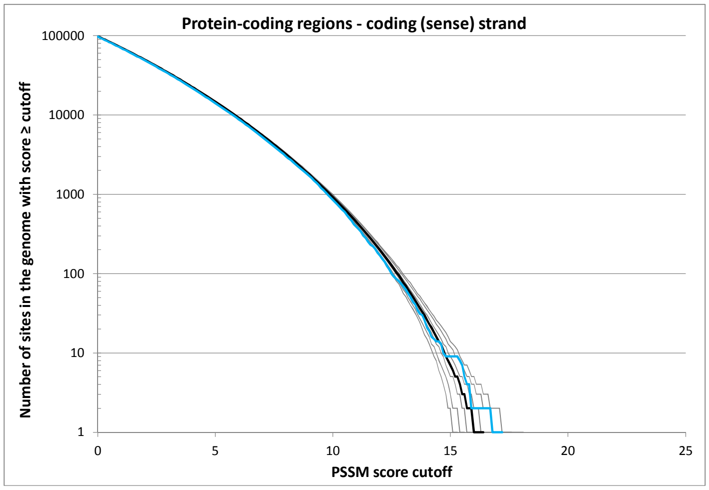

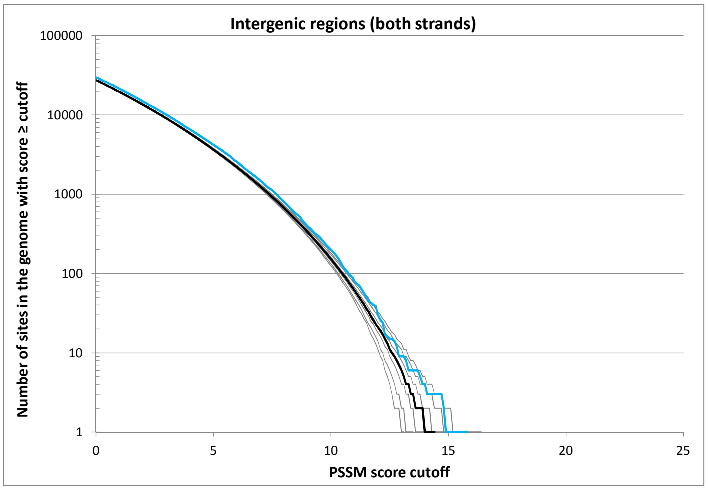

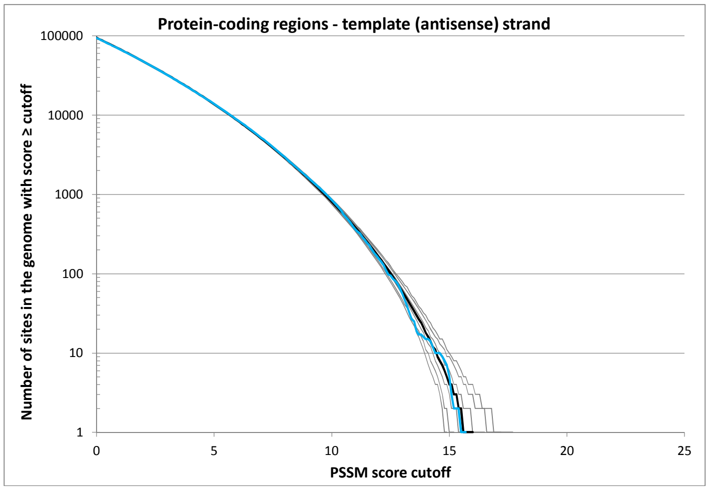

ArgR (*E. coli*)

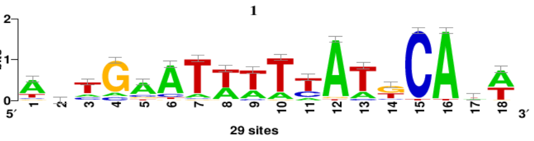

Motif length: 18
Motif information content: 15.75 bits
Max possible PSSM score: 24.59
Motif GC content: 25.29 %
Genome GC content: 50.79 %

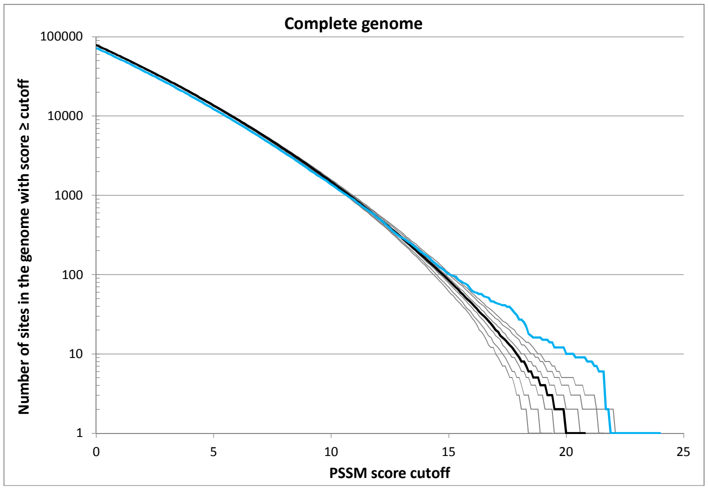

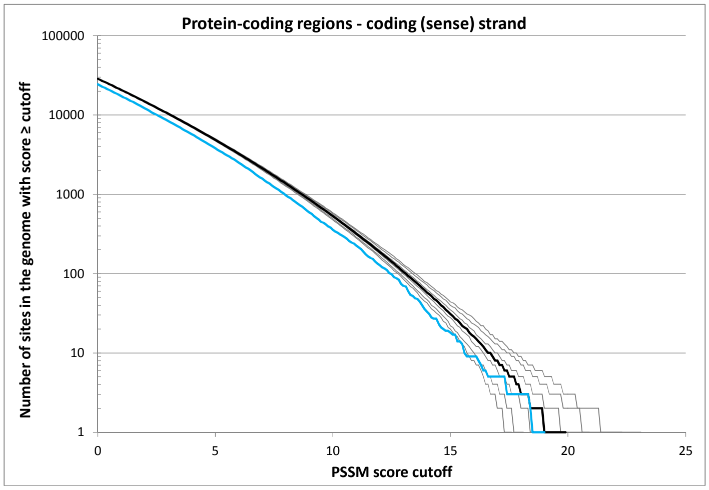

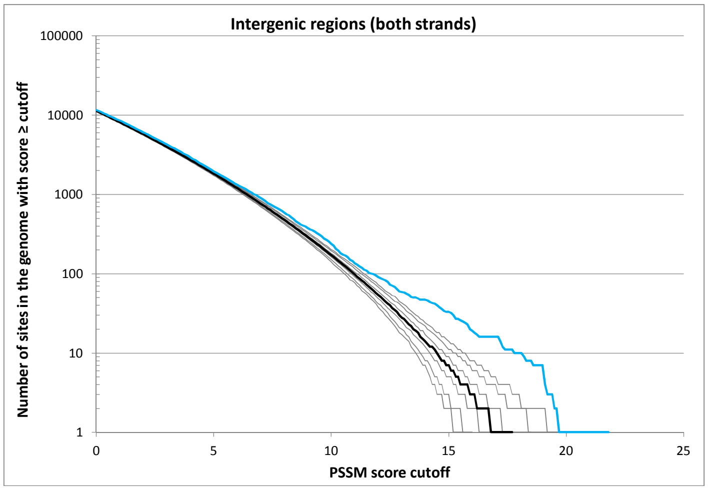

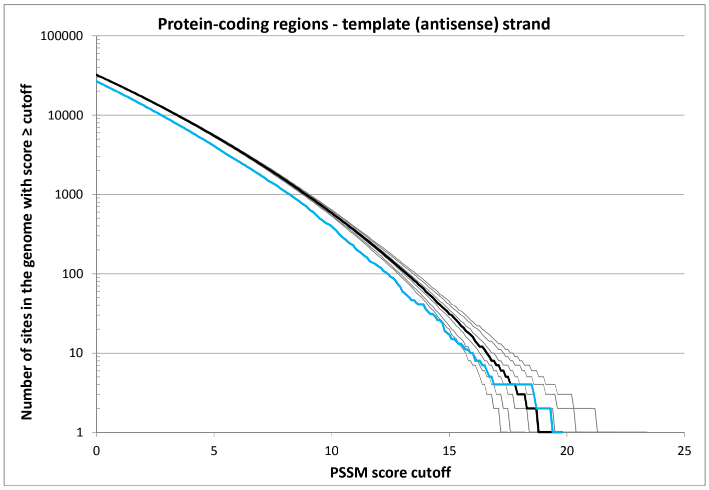

CpxR (*E. coli*)

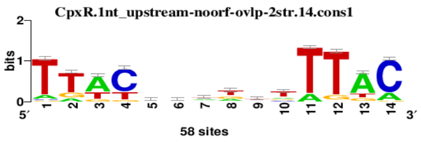

Motif length: 14
Motif information content: 9.00 bits
Max possible PSSM score: 16.98
Motif GC content: 31.40 %
Genome GC content: 50.79 %

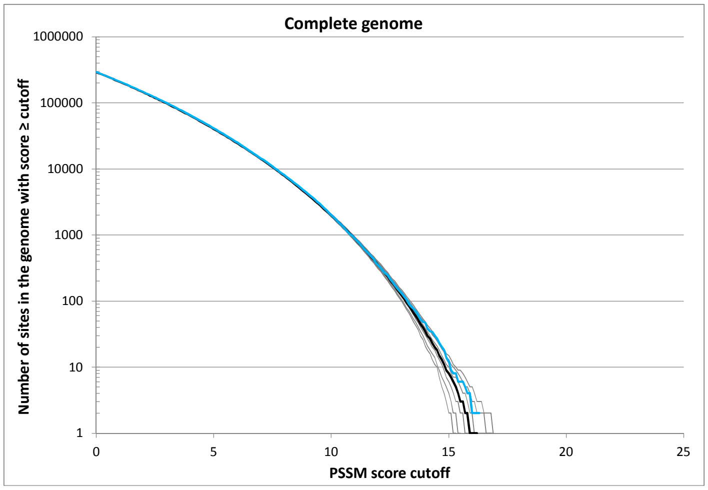

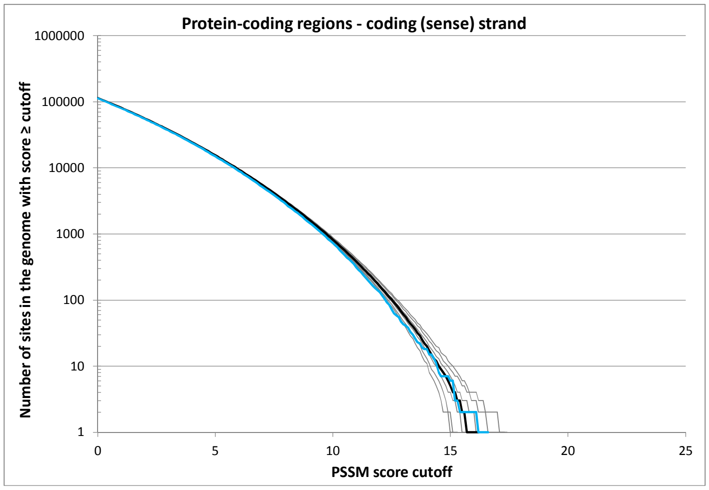

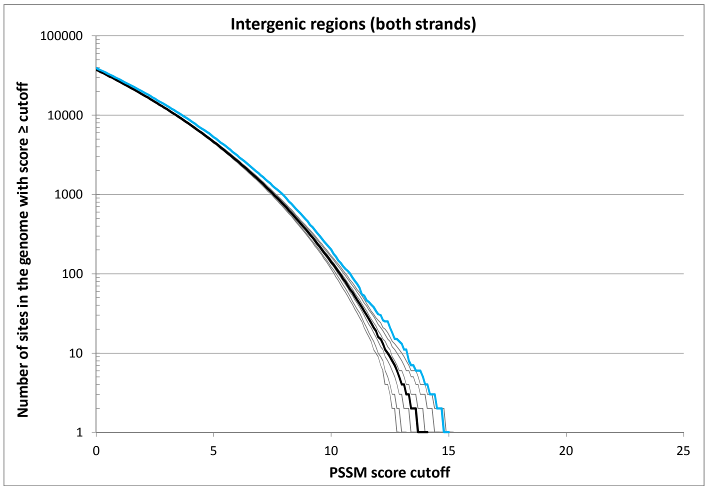

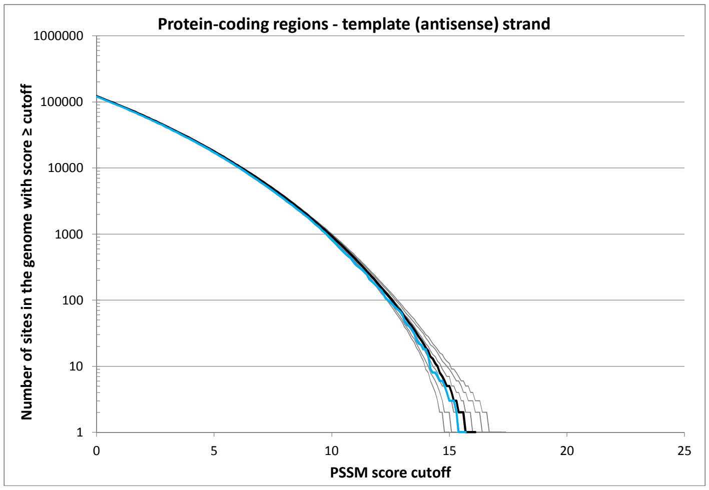

Cra (*E. coli*)

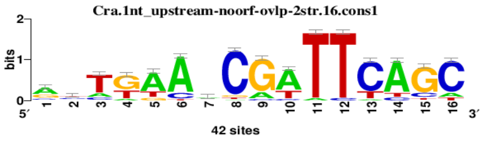

Motif length: 16
Motif information content: 14.68 bits
Max possible PSSM score: 23.24
Motif GC content: 41.07 %
Genome GC content: 50.79 %

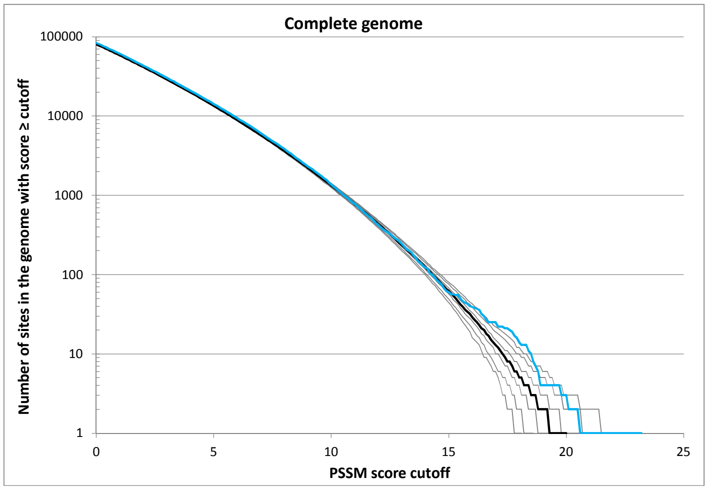

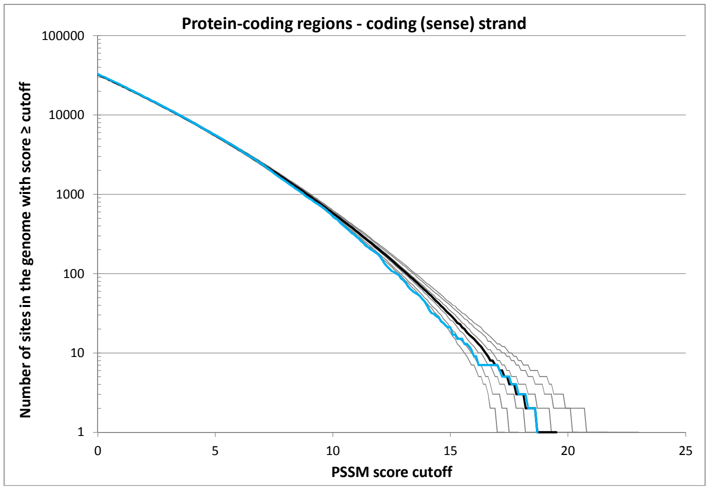

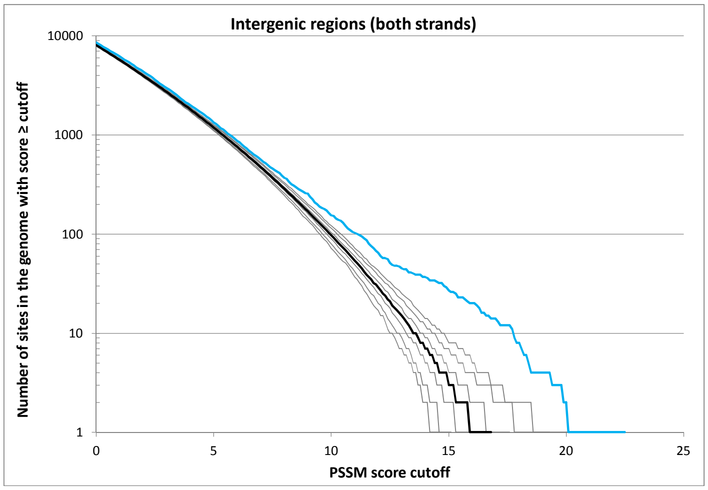

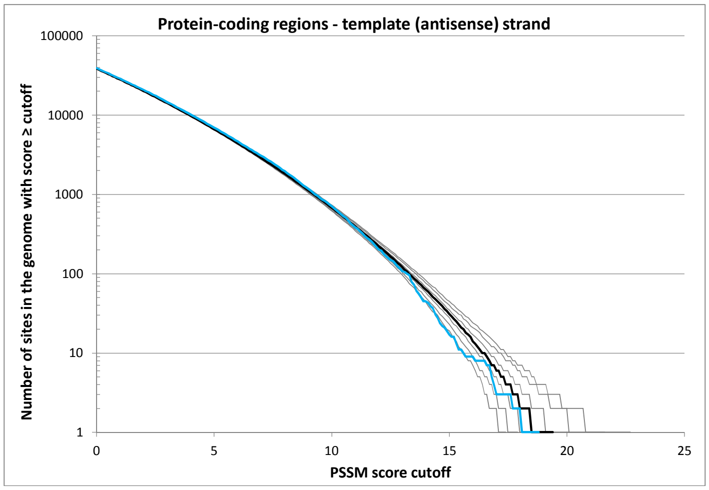

Crp (*E. coli*)

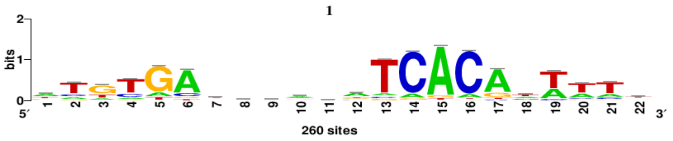

Motif length: 22
Motif information content: 11.31 bits
Max possible PSSM score: 25.13
Motif GC content: 36.94 %
Genome GC content: 50.79 %

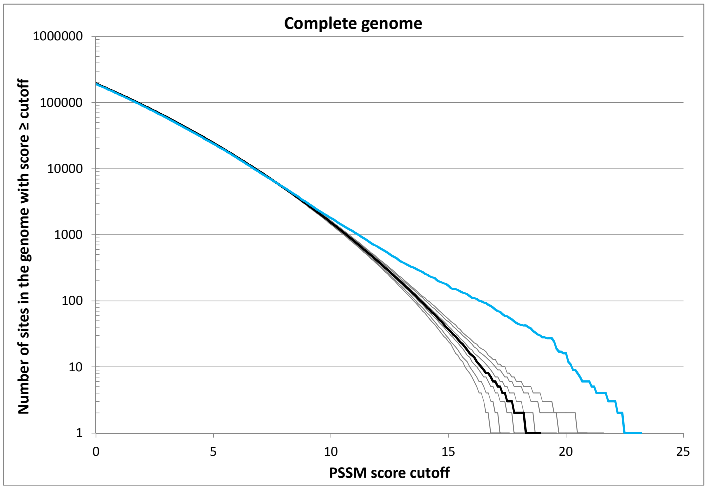

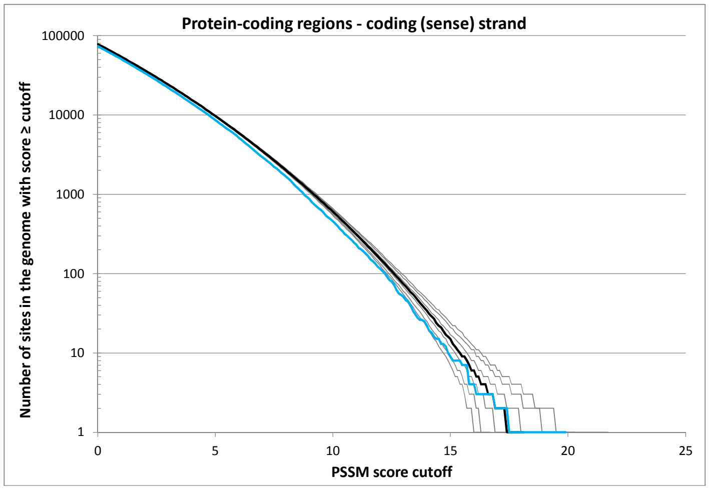

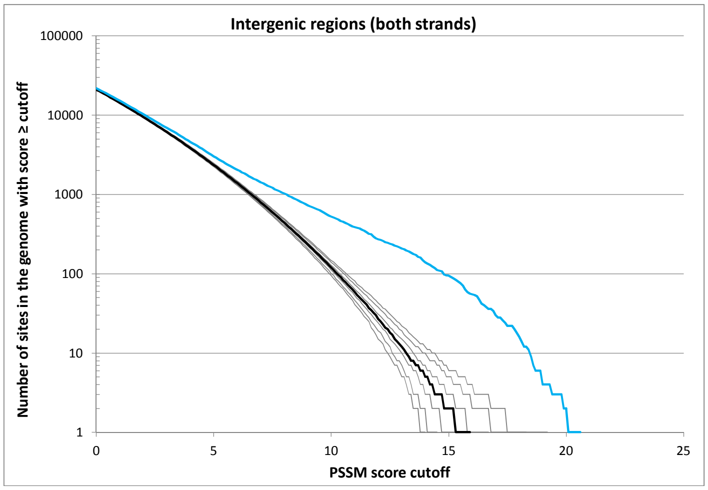

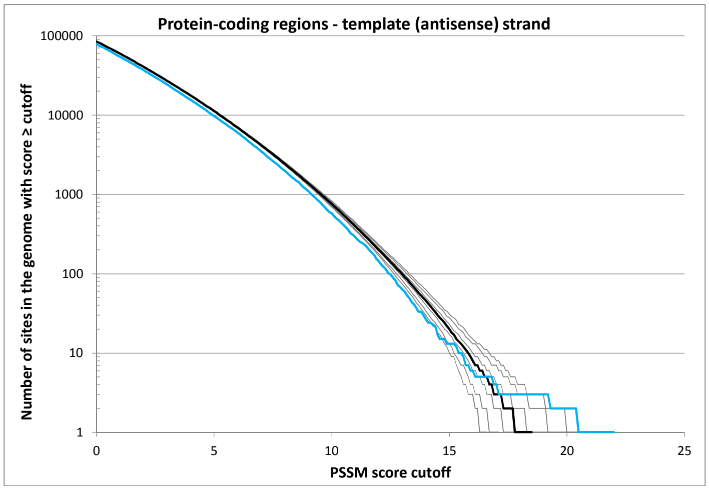

CsgD (*E. coli*)

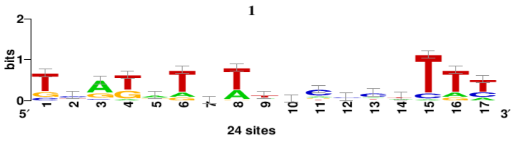

Motif length: 17
Motif information content: 8.41 bits
Max possible PSSM score: 18.87
Motif GC content: 35.78 %
Genome GC content: 50.79 %

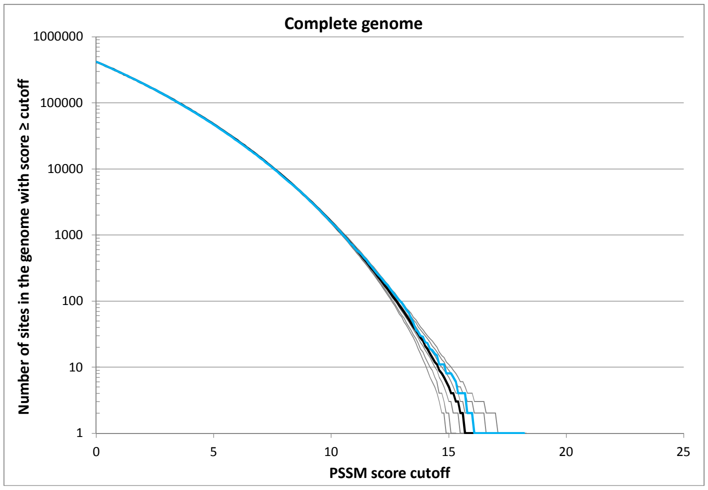

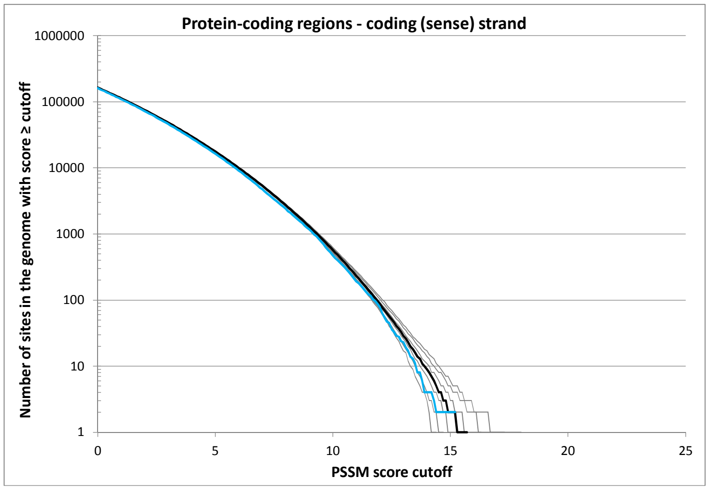

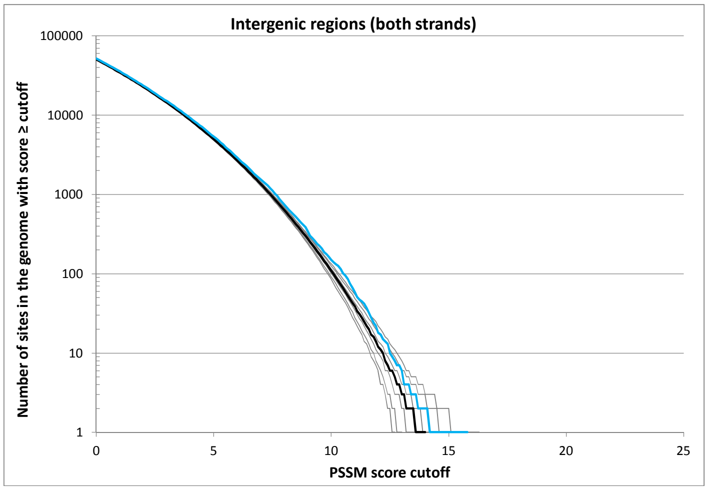

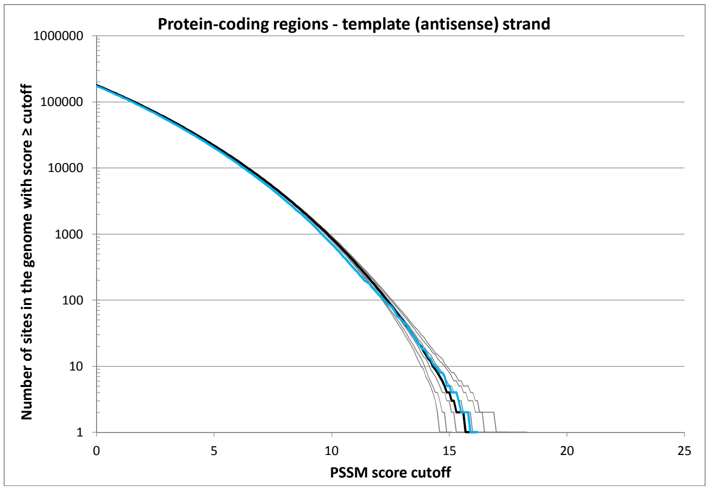

CysB (*E. coli*)

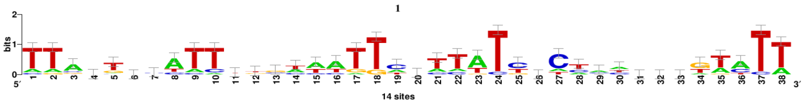

Motif length: 38
Motif information content: 23.94 bits
Max possible PSSM score: 42.12
Motif GC content: 30.64 %
Genome GC content: 50.79 %

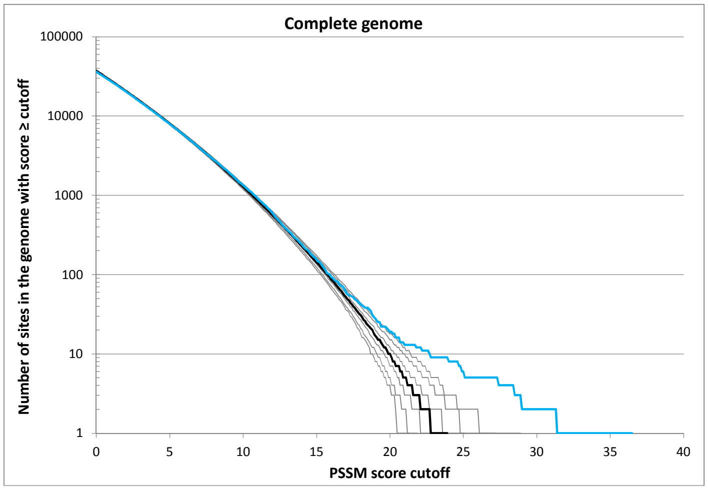

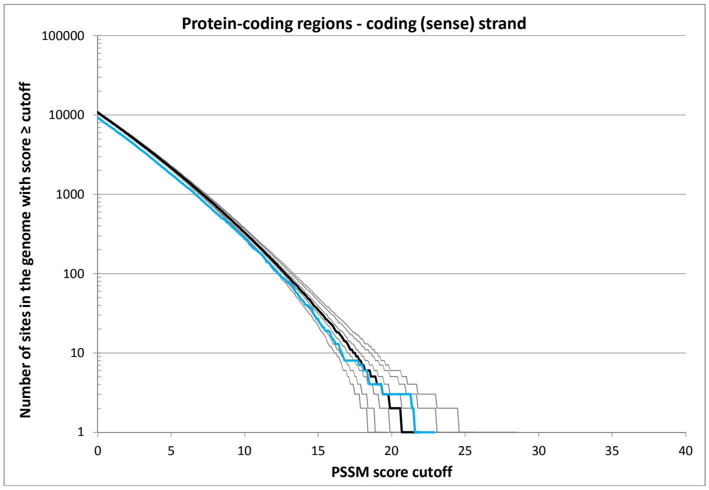

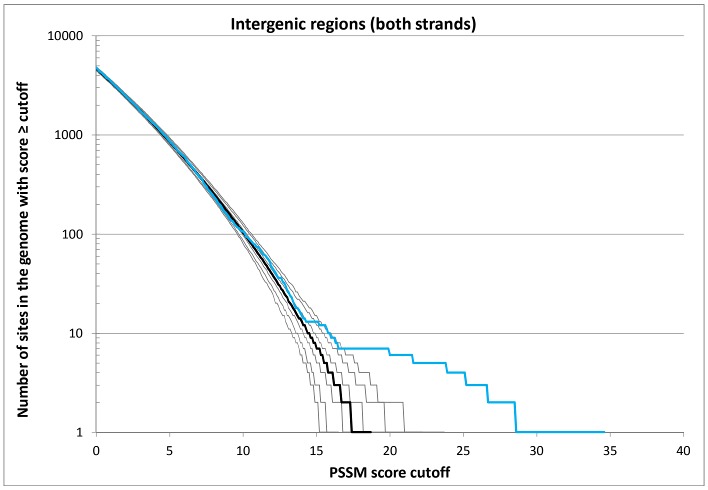

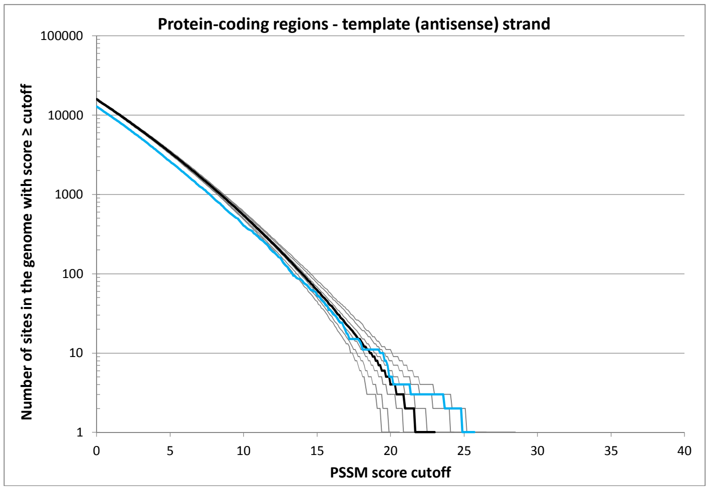

CytR (*E. coli*)

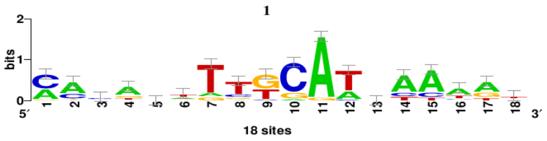

Motif length: 18
Motif information content: 11.33 bits
Max possible PSSM score: 21.23
Motif GC content: 33.64 %
Genome GC content: 50.79 %

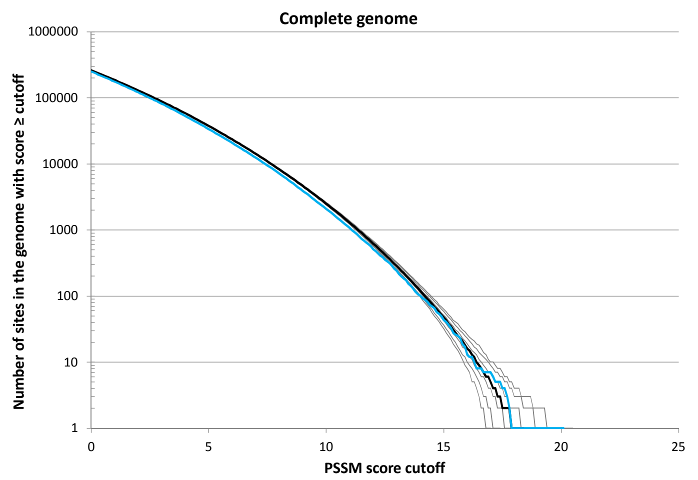

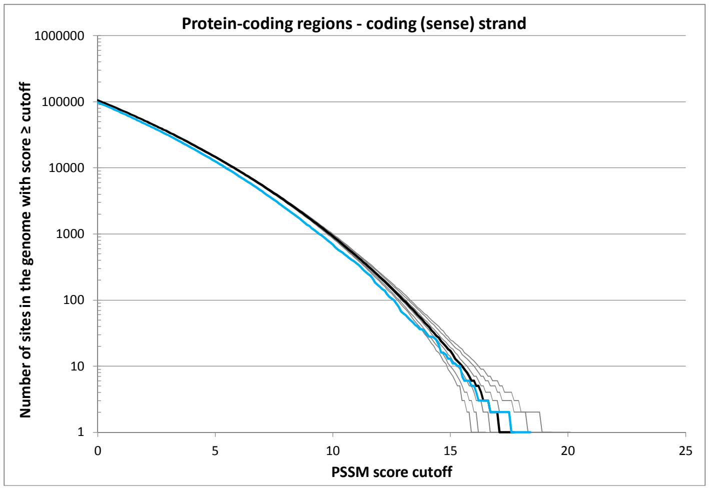

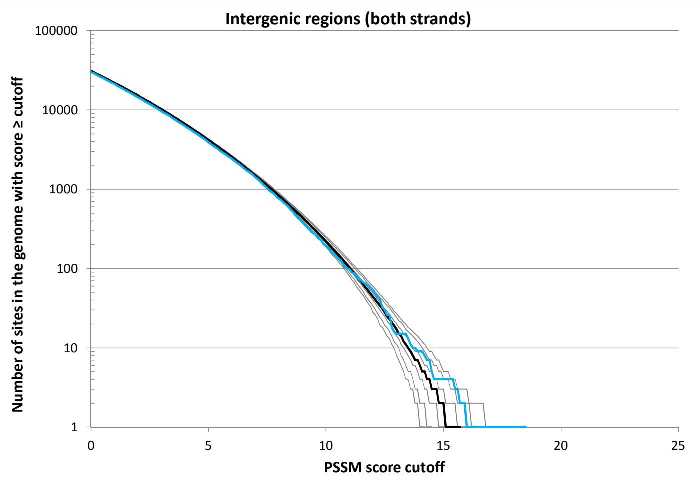

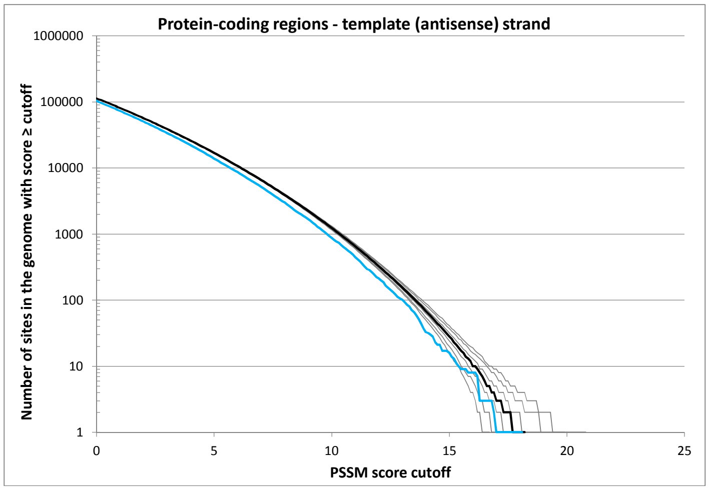

DnaA (*E. coli*)

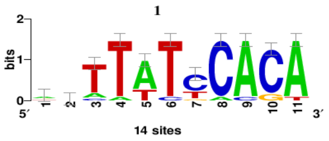

Motif length: 11
Motif information content: 13.01 bits
Max possible PSSM score: 16.70
Motif GC content: 32.47 %
Genome GC content: 50.79 %

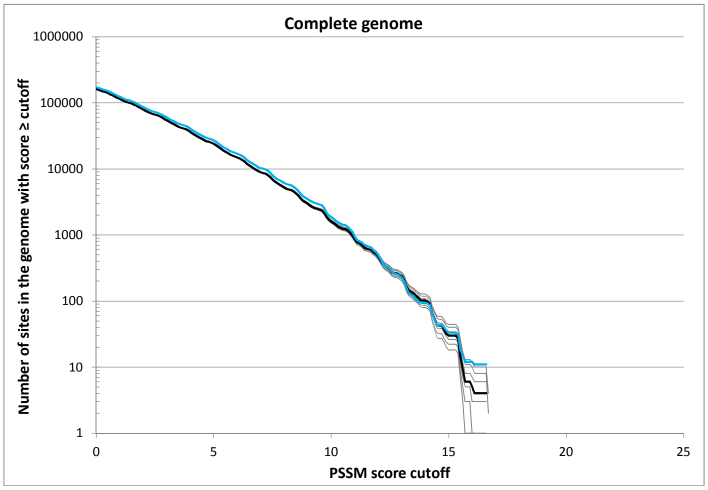

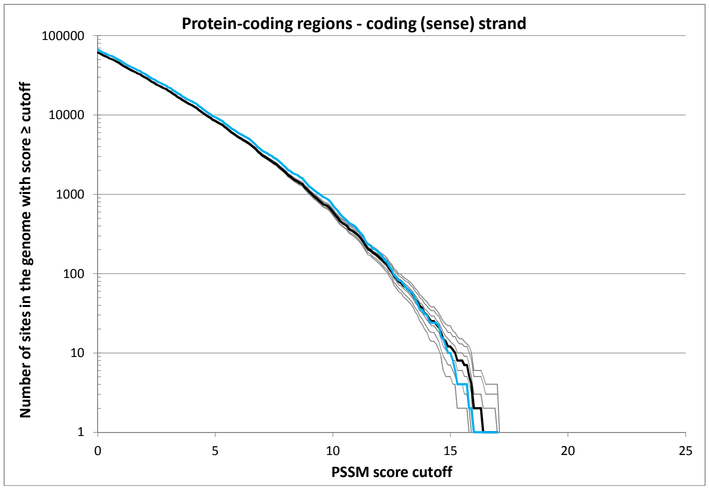

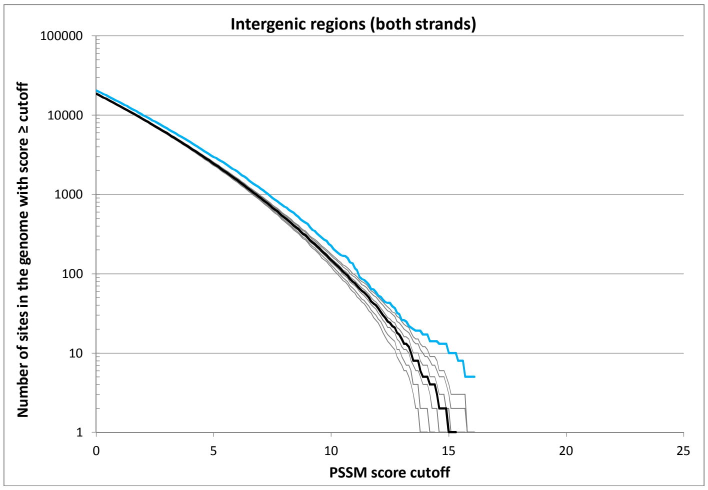

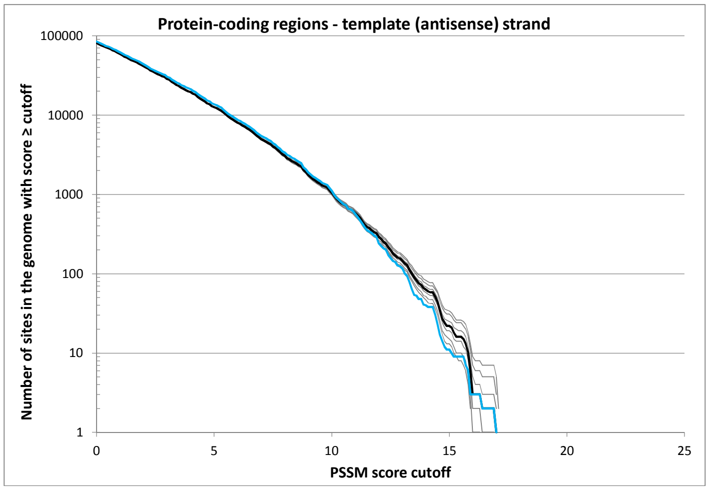

FadR (*E. coli*)

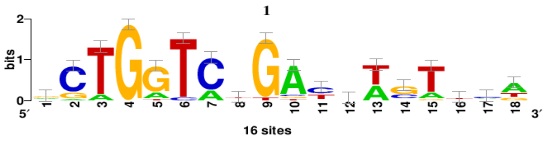

Motif length: 18
Motif information content: 15.65 bits
Max possible PSSM score: 23.41
Motif GC content: 49.31 %
Genome GC content: 50.79 %

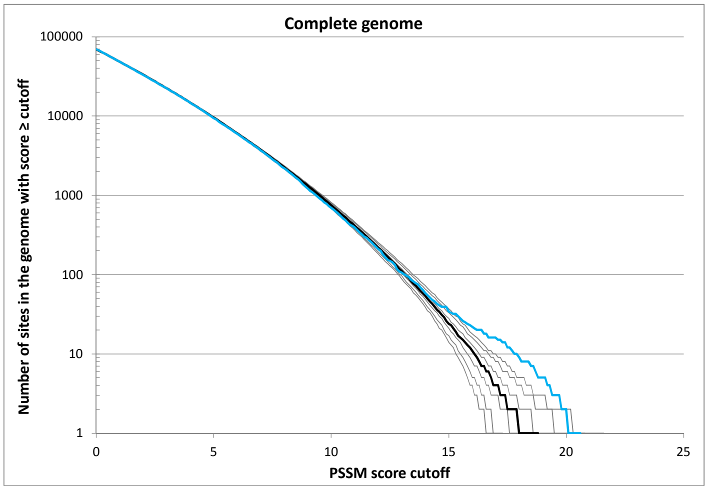

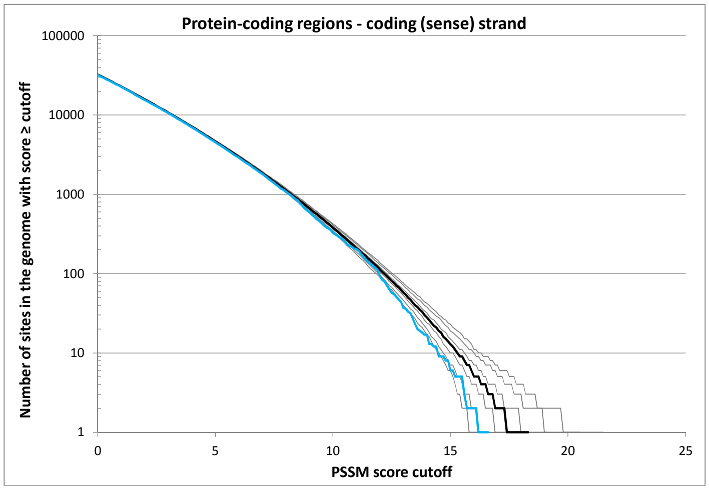

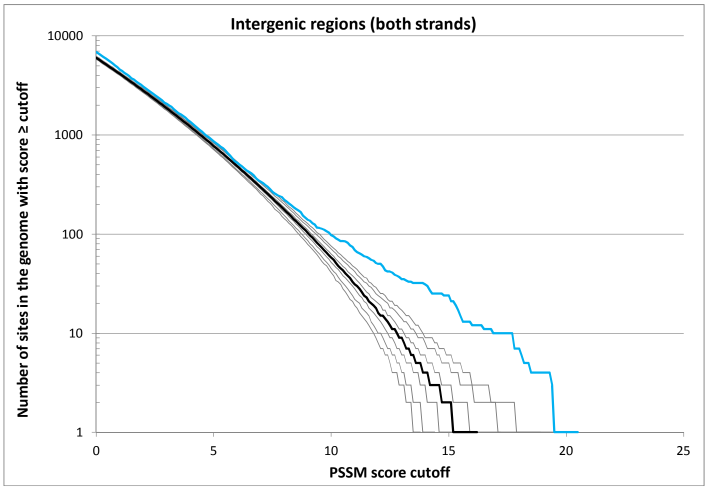

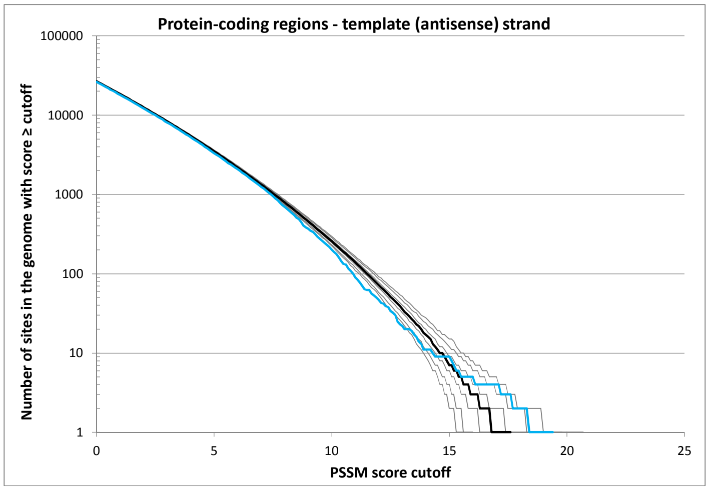

Fis (*E. coli*)

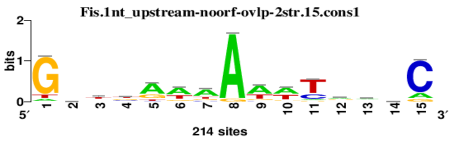

Motif length: 15
Motif information content: 6.87 bits
Max possible PSSM score: 15.00
Motif GC content: 39.41 %
Genome GC content: 50.79 %

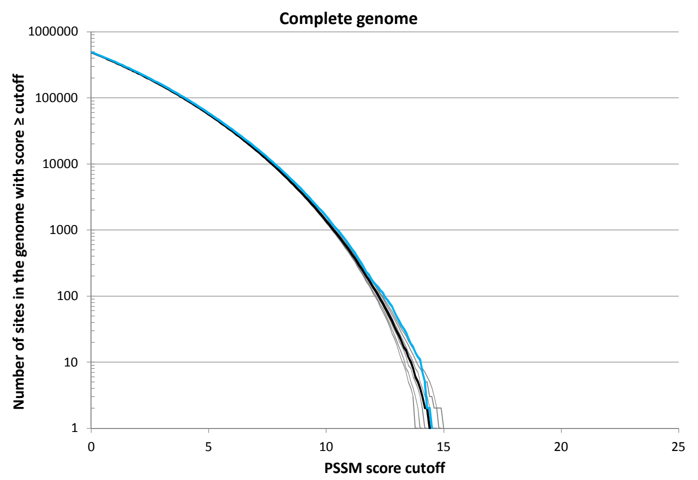

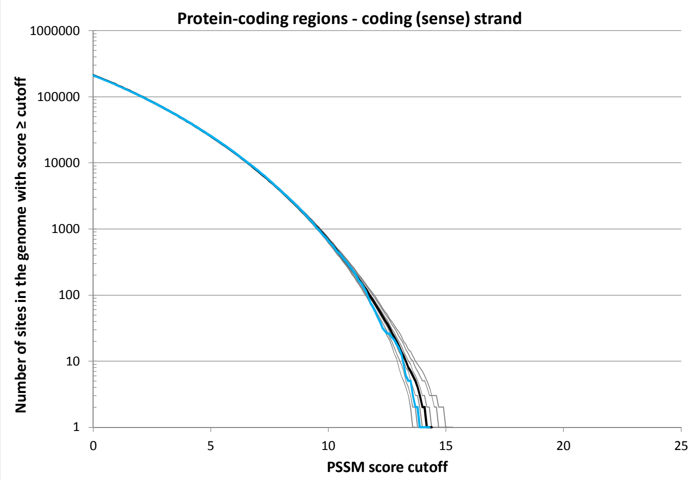

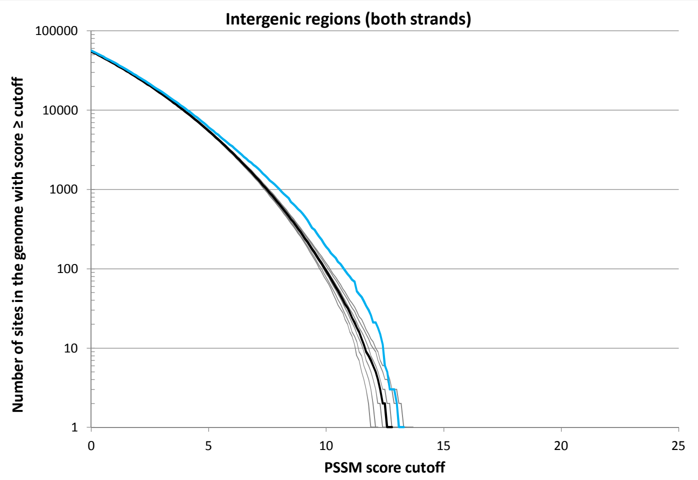

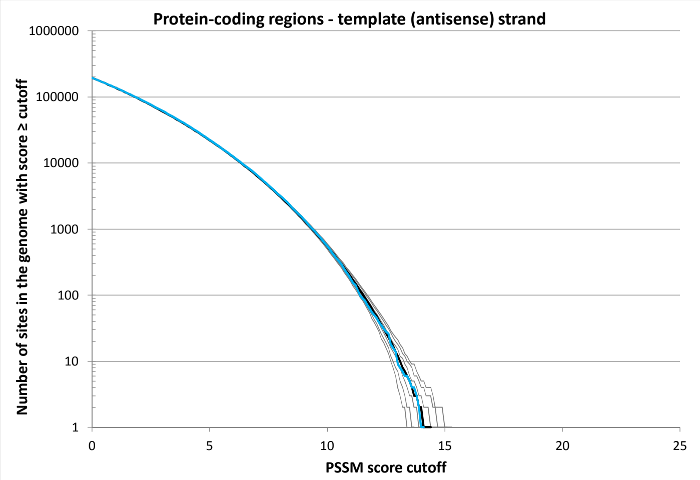

FlhDC (*E. coli*)

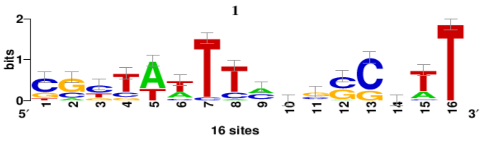

Motif length: 16
Motif information content: 12.83 bits
Max possible PSSM score: 19.22
Motif GC content: 47.66 %
Genome GC content: 50.79 %

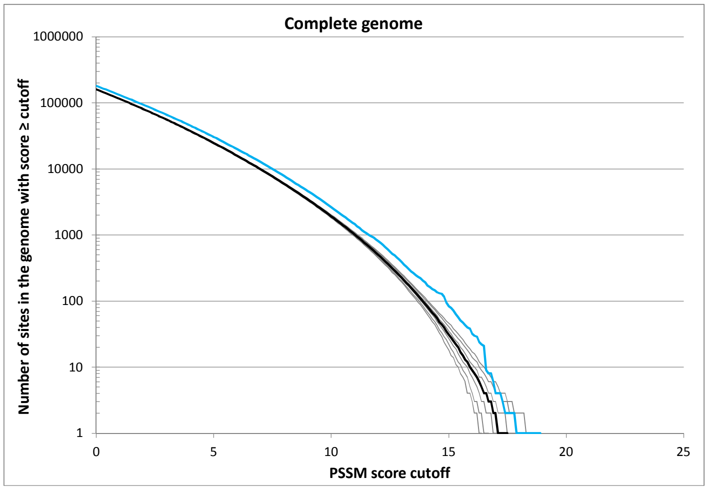

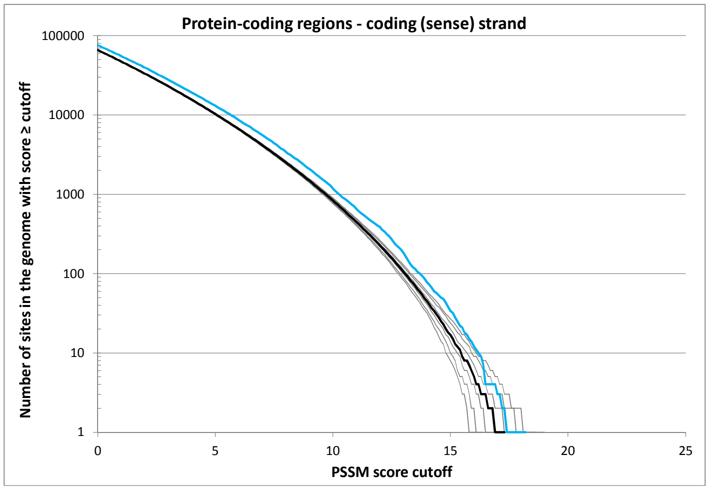

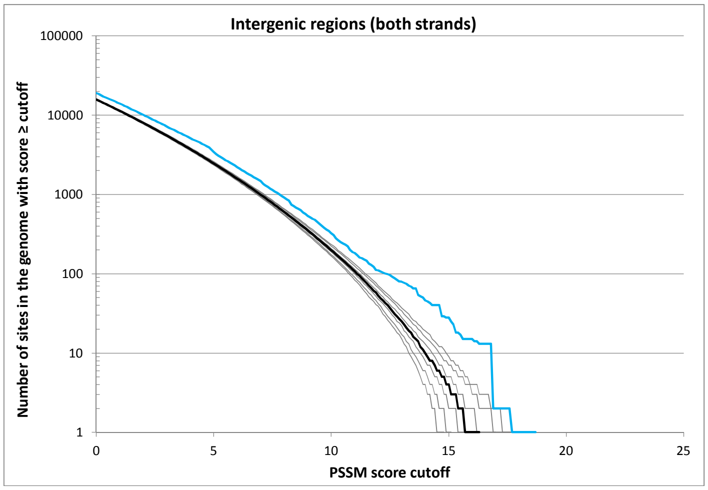

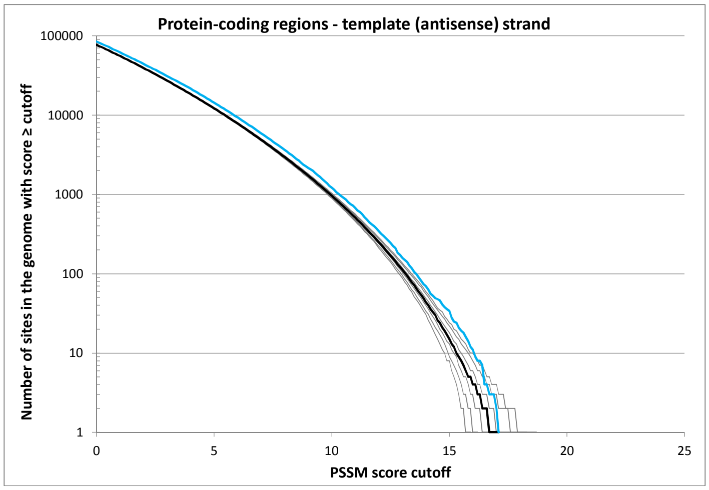

FNR (*E. coli*)

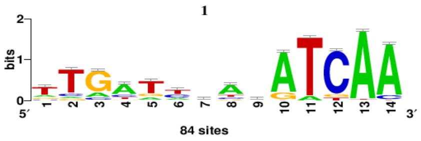

Motif length: 14
Motif information content: 11.06 bits
Max possible PSSM score: 19.17
Motif GC content: 30.10 %
Genome GC content: 50.79 %

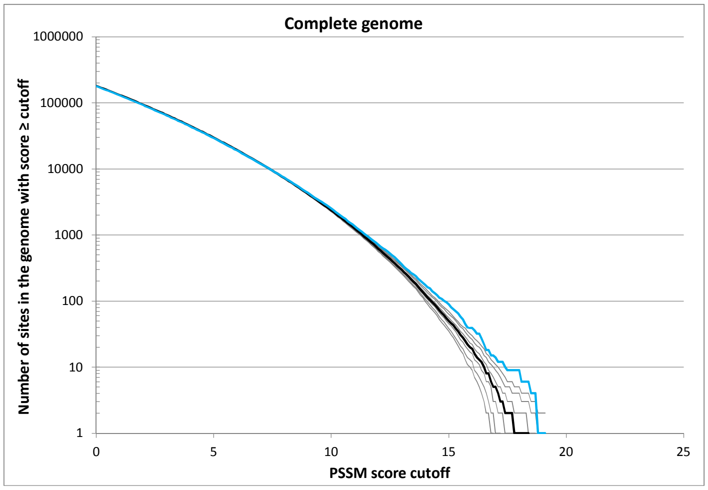

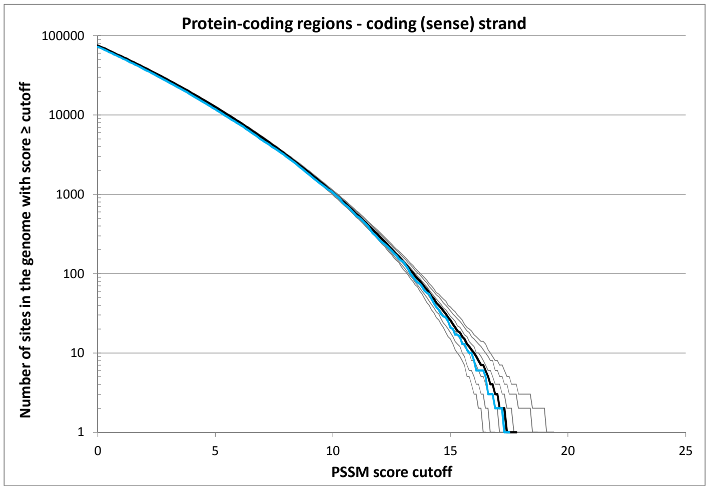

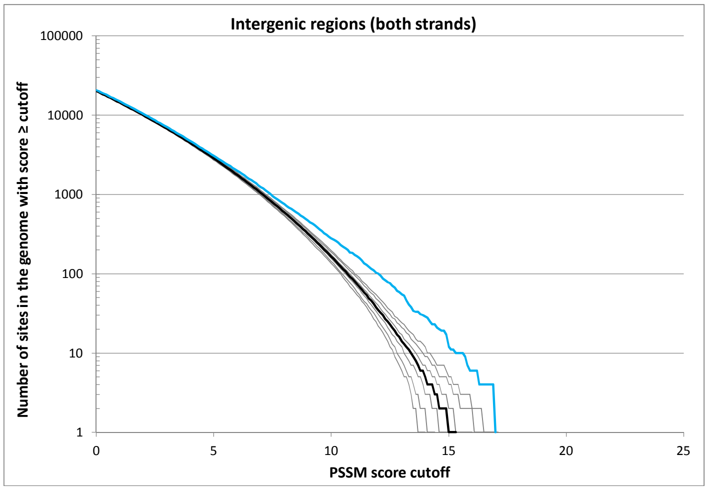

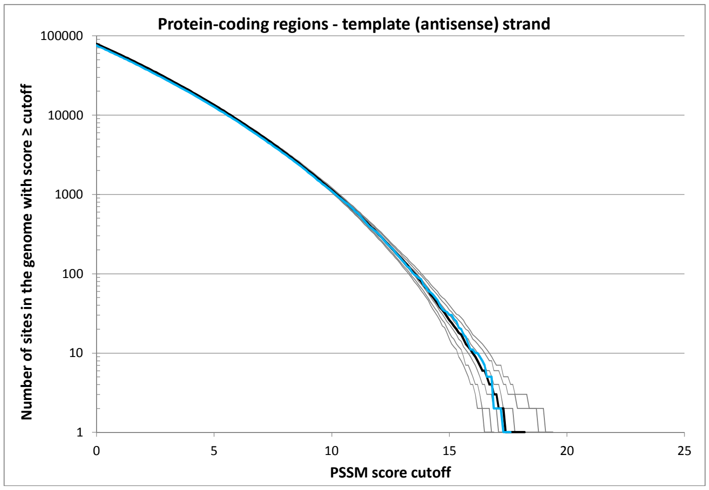

FNR (*R. sphaeroides*)

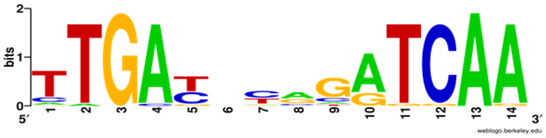

Motif length: 14
Motif information content: 17.13 bits
Max possible PSSM score: 26.44
Motif GC content: 39.95 %
Genome GC content: 69.01 %

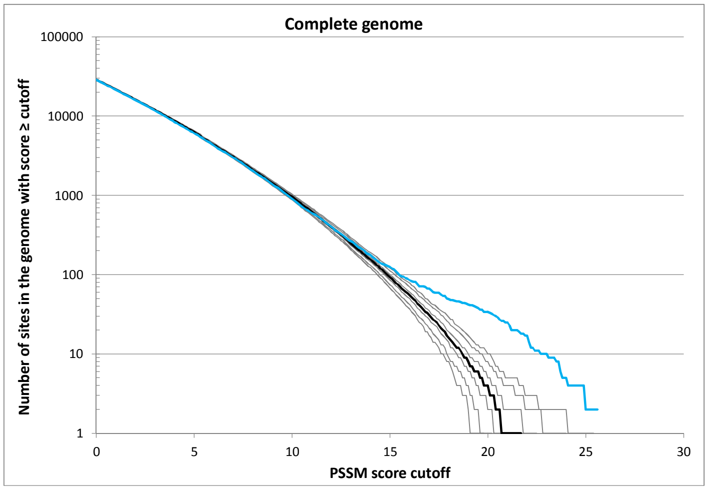

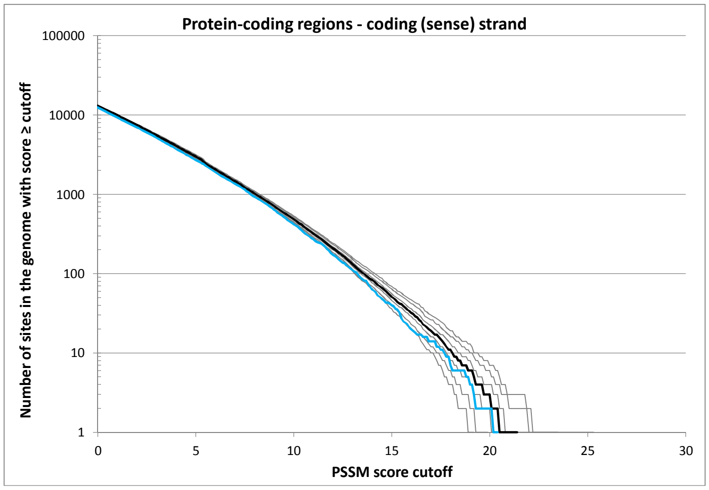

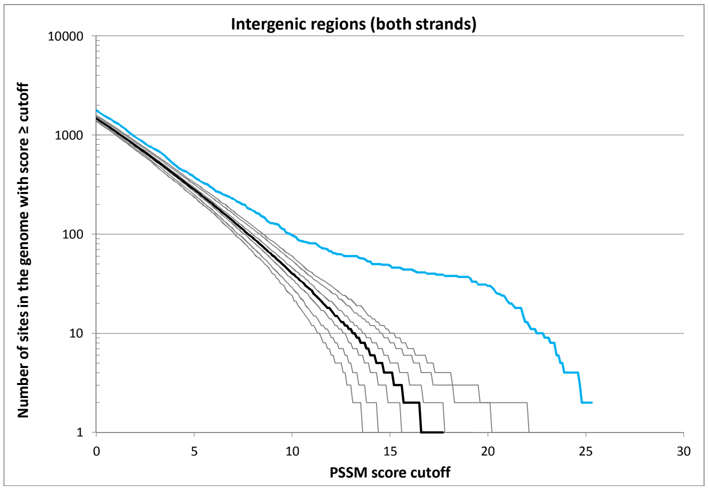

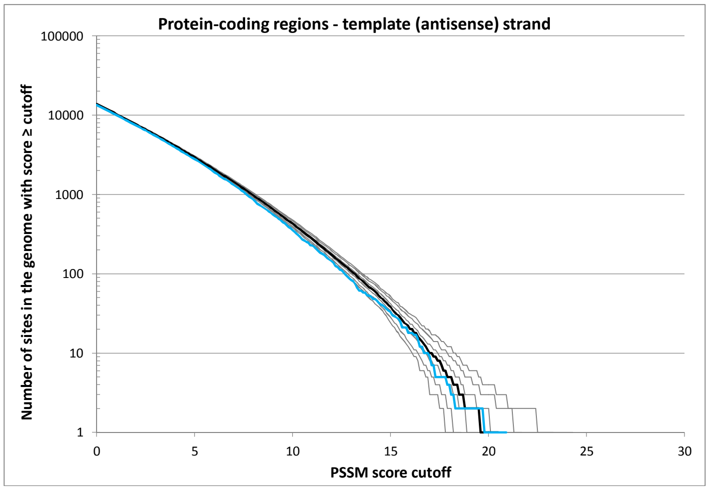

Fur (*E. coli*)

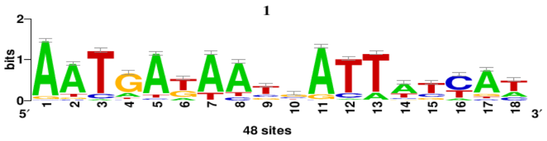

Motif length: 18
Motif information content: 15.78 bits
Max possible PSSM score: 26.69
Motif GC content: 24.19 %
Genome GC content: 50.79 %

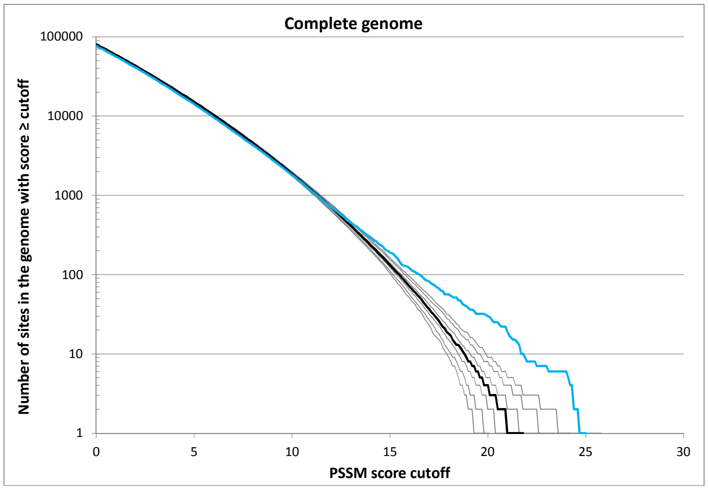

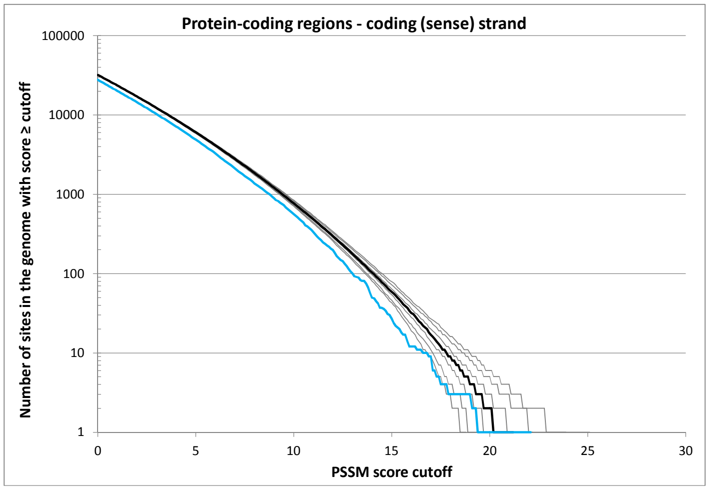

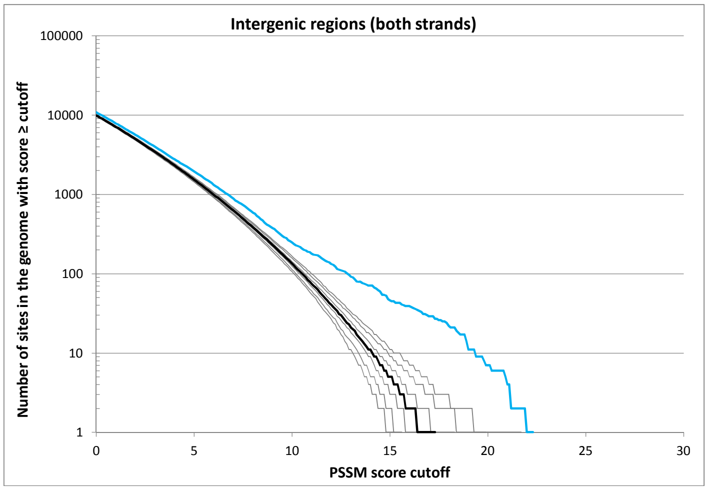

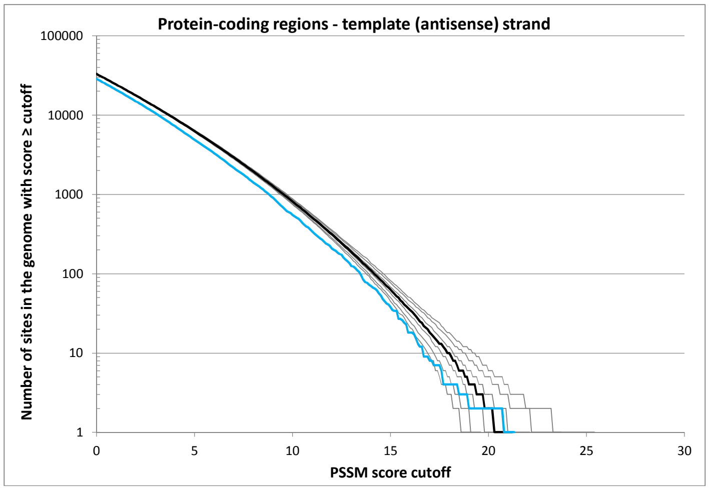

GadW (*E. coli*)

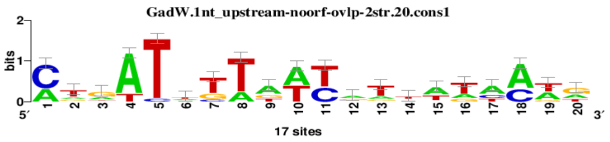

Motif length: 20
Motif information content: 14.55 bits
Max possible PSSM score: 23.79
Motif GC content: 25.88 %
Genome GC content: 50.79 %

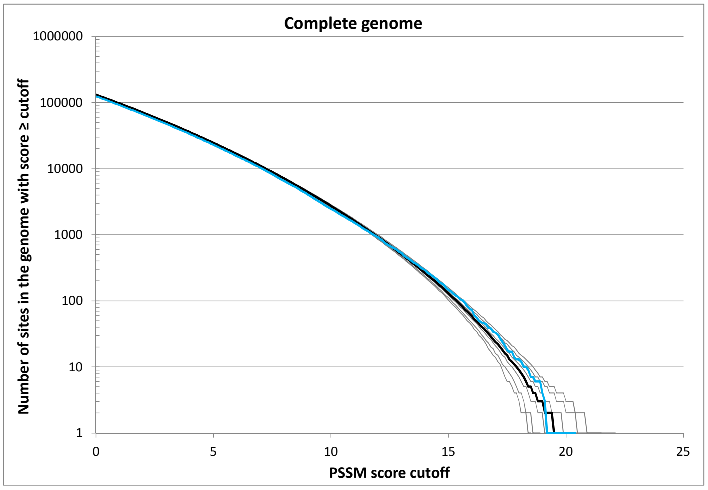

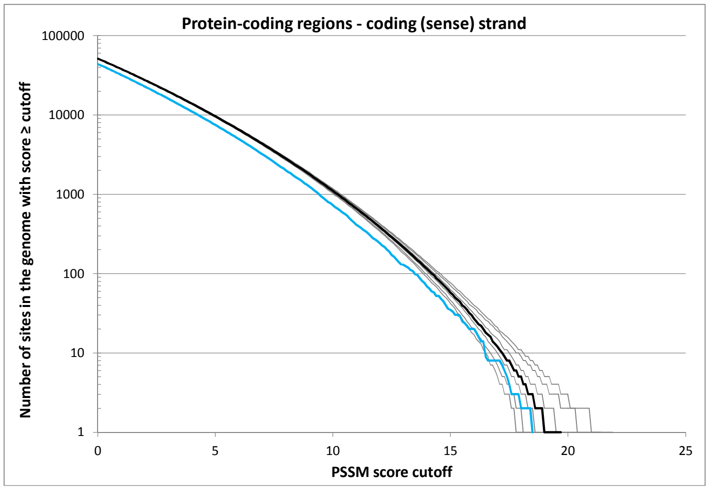

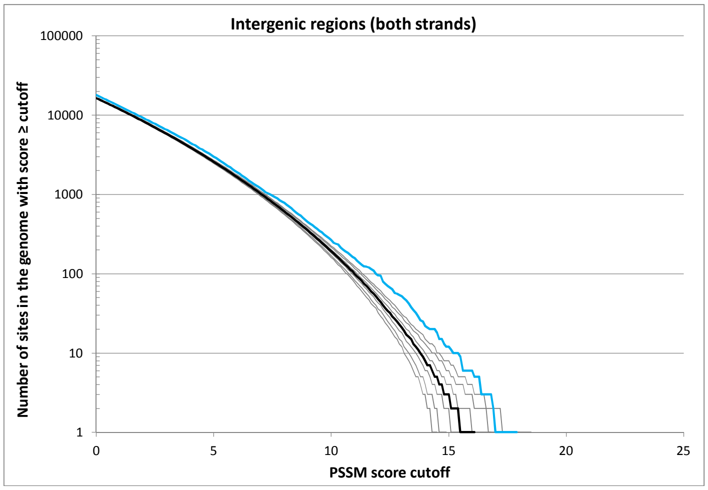

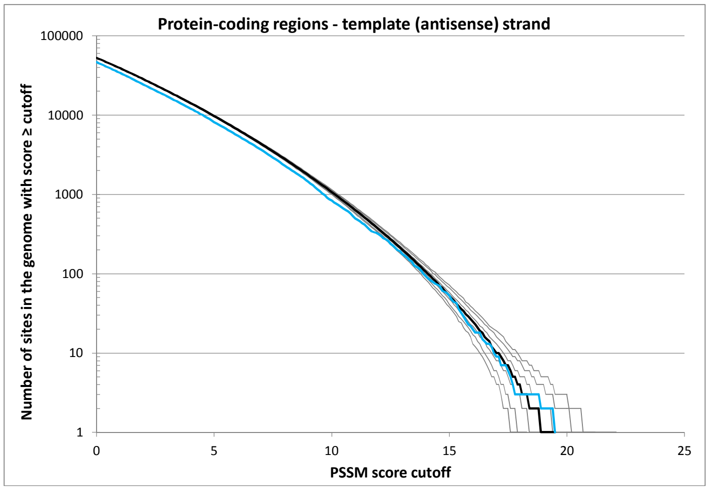

GadX (*E. coli*)

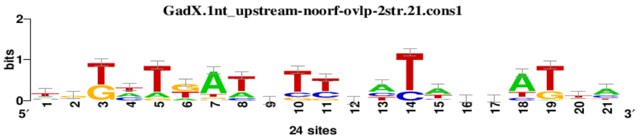

Motif length: 21
Motif information content: 11.33 bits
Max possible PSSM score: 22.78
Motif GC content: 30.36 %
Genome GC content: 50.79 %

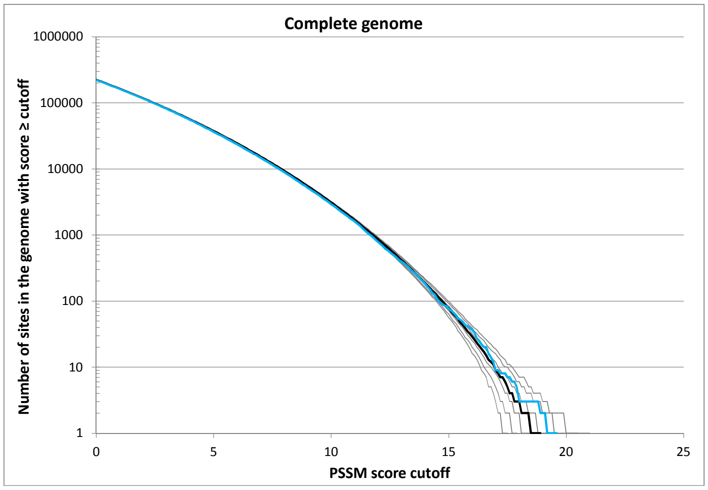

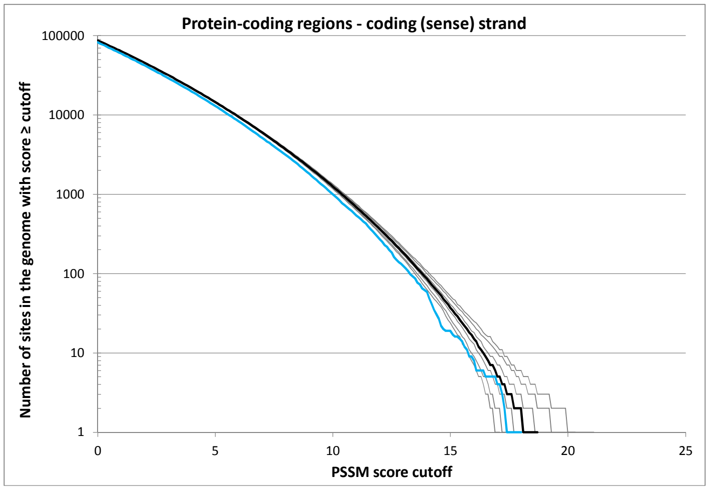

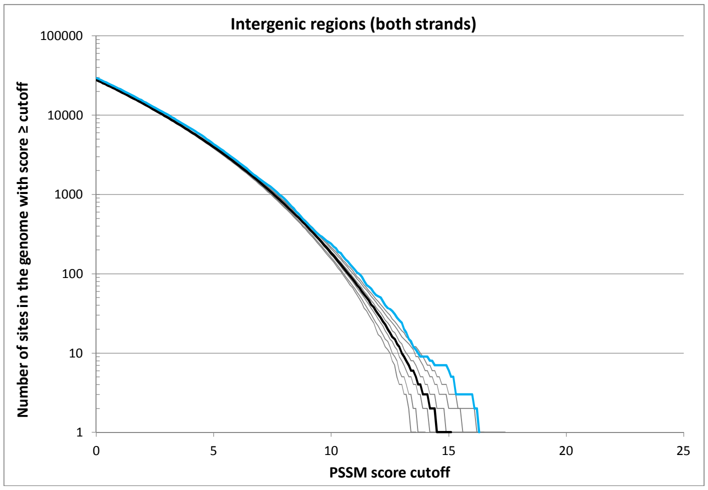

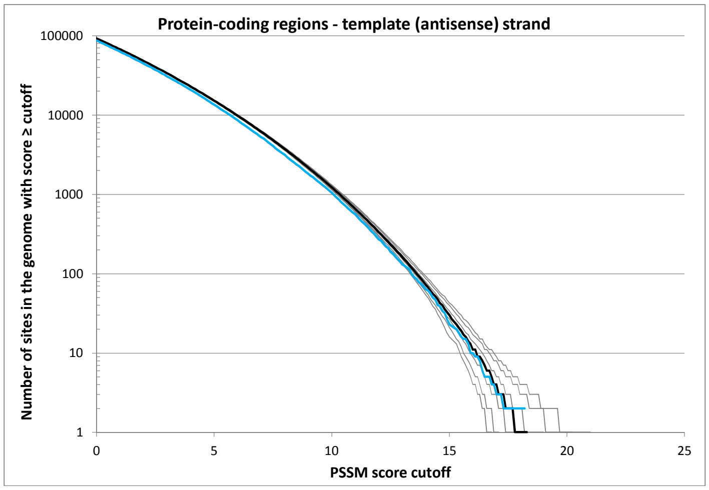

GalR (*E. coli*)

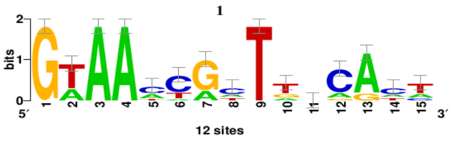

Motif length: 15
Motif information content: 16.27 bits
Max possible PSSM score: 20.83
Motif GC content: 39.44 %
Genome GC content: 50.79 %

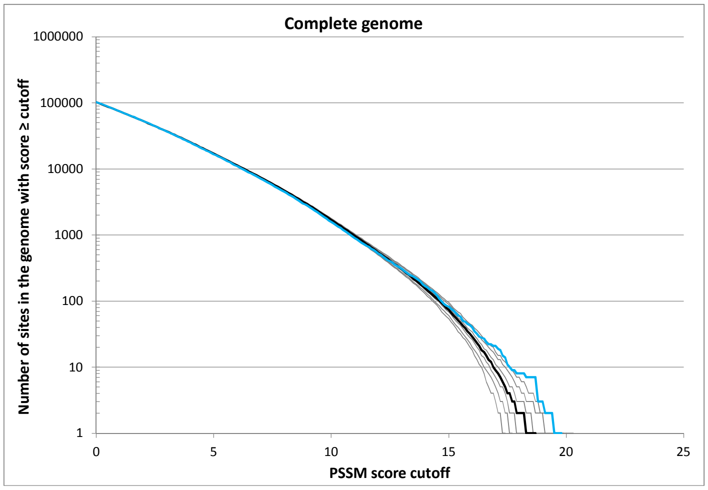

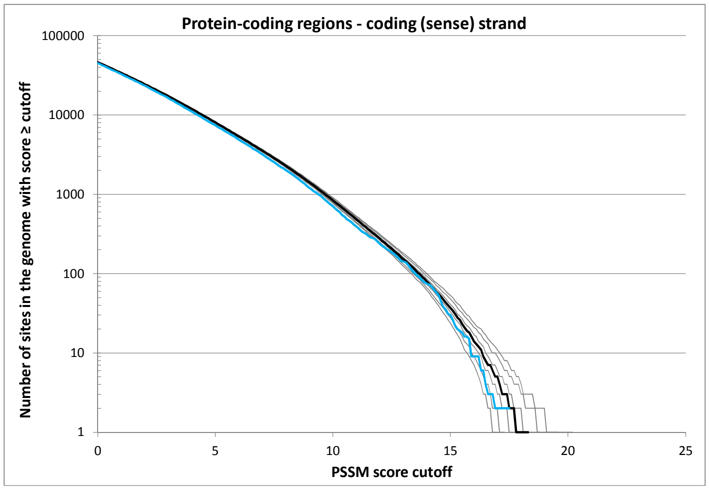

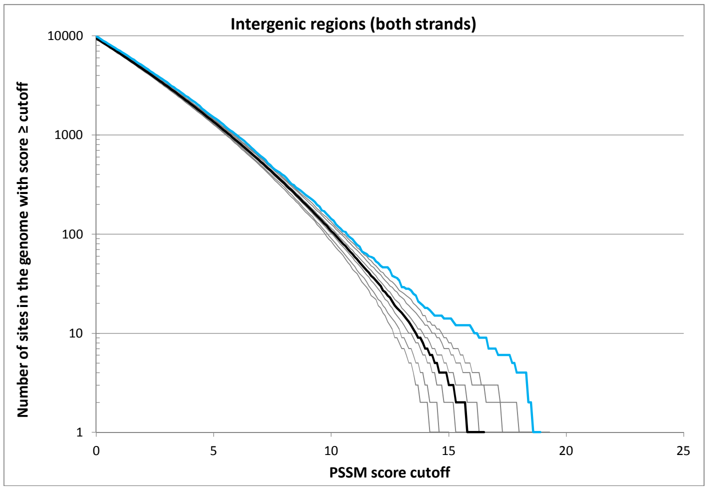

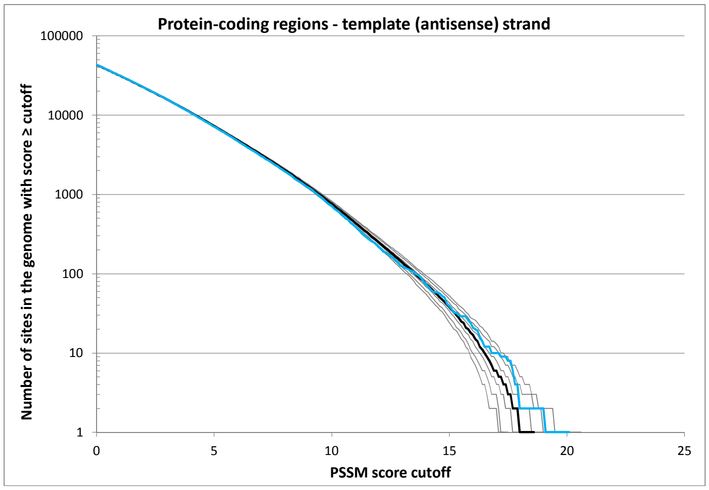

GlpR (*E. coli*)

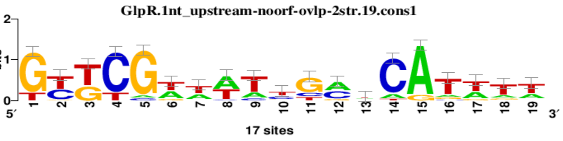

Motif length: 19
Motif information content: 15.30 bits
Max possible PSSM score: 23.98
Motif GC content: 39.63 %
Genome GC content: 50.79 %

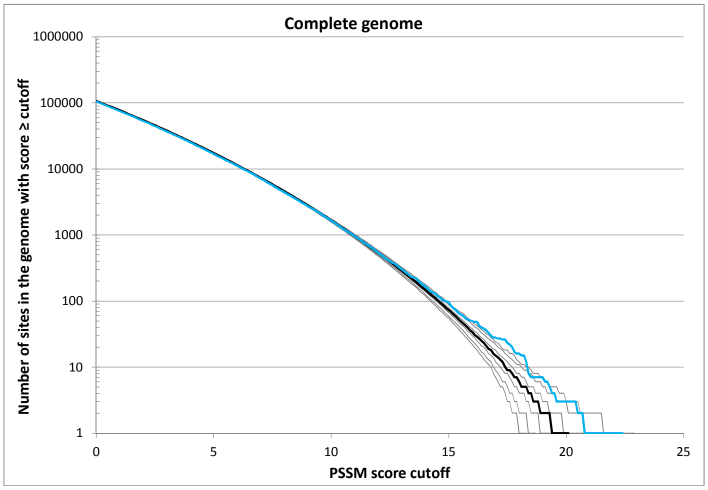

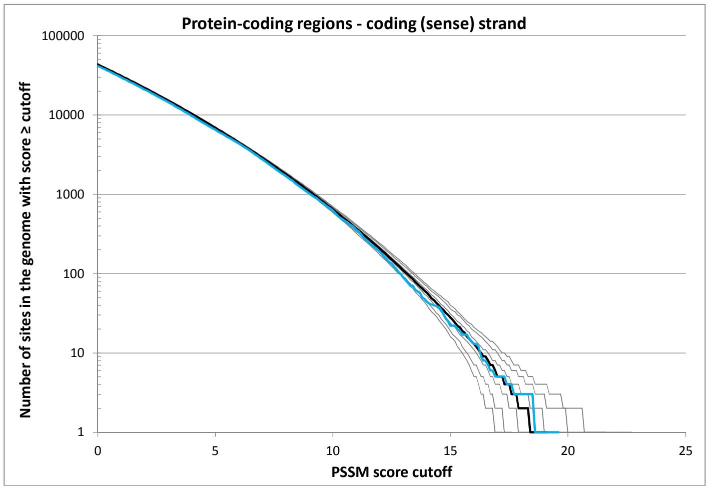

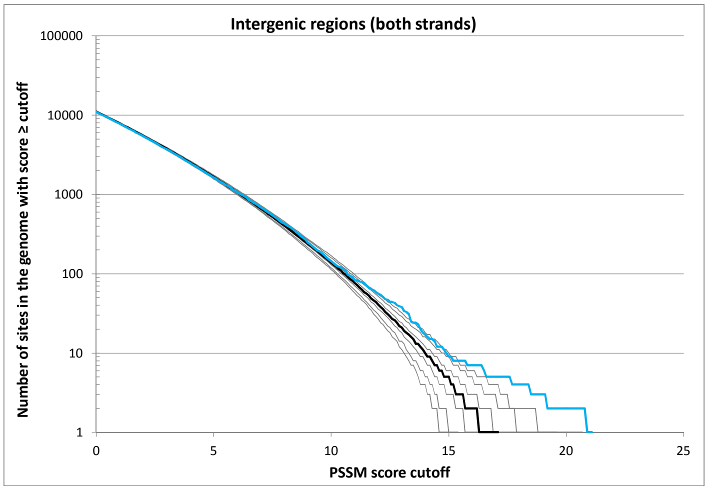

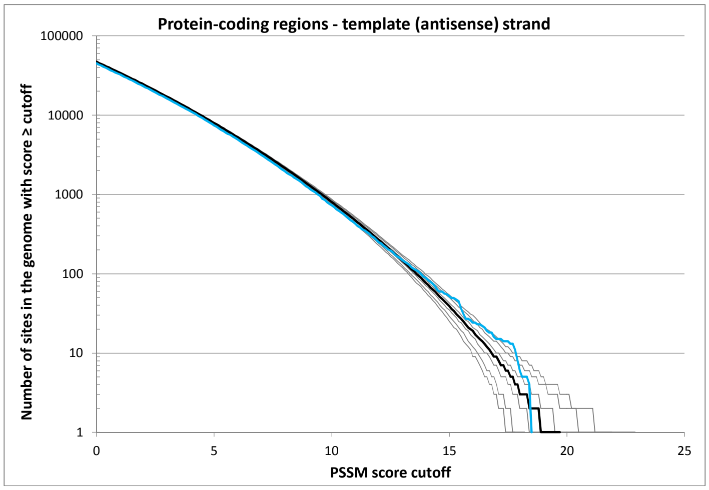

H-NS (*E. coli*)

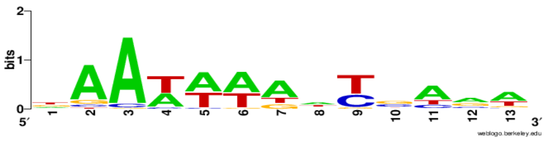

Motif length: 13
Motif information content: 8.22 bits
Max possible PSSM score: 14.84
Motif GC content: 24.39 %
Genome GC content: 50.79 %

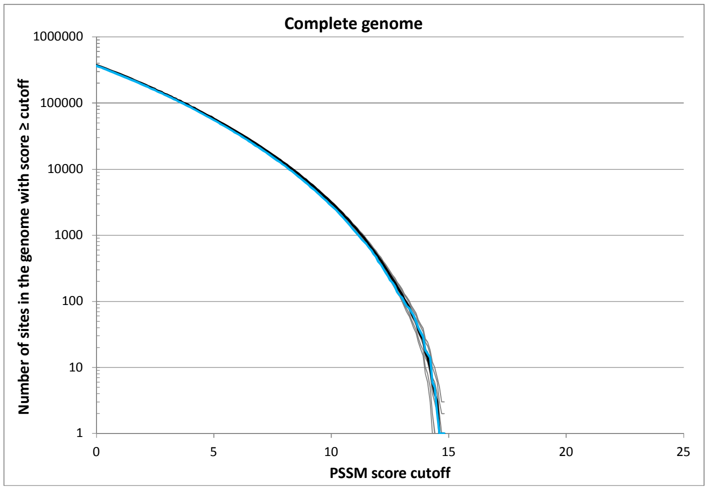

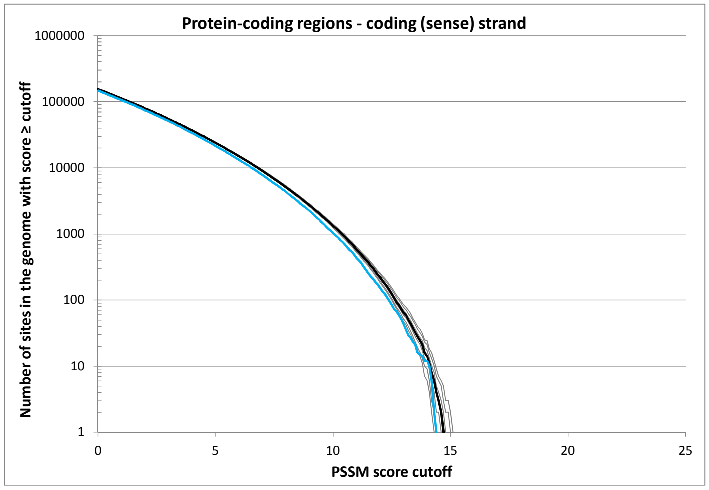

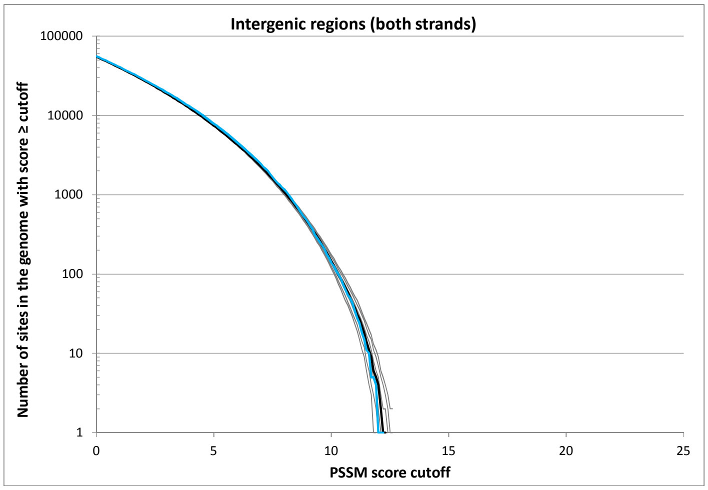

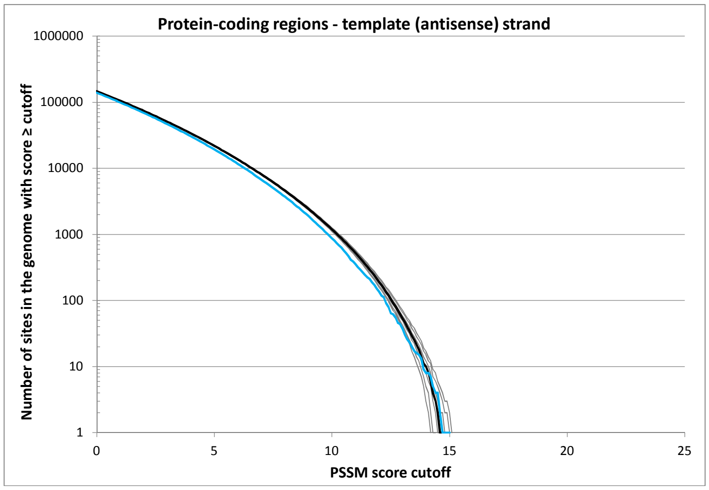

IHF (*E. coli*)

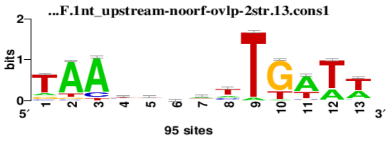

Motif length: 13
Motif information content: 8.39 bits
Max possible PSSM score: 15.85
Motif GC content: 25.83 %
Genome GC content: 50.79 %

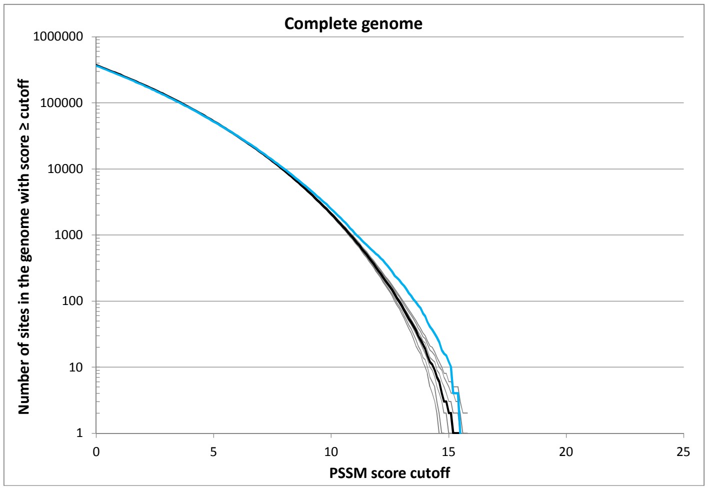

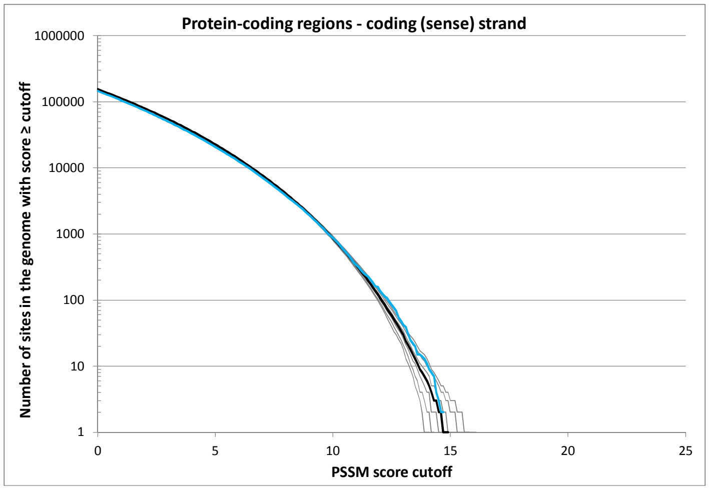

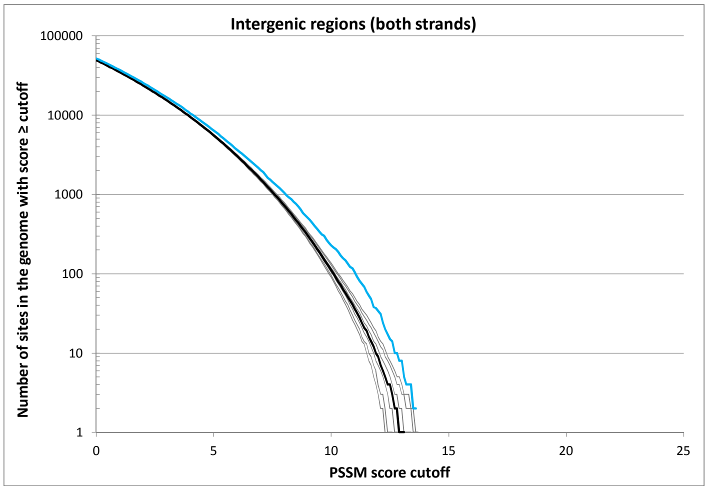

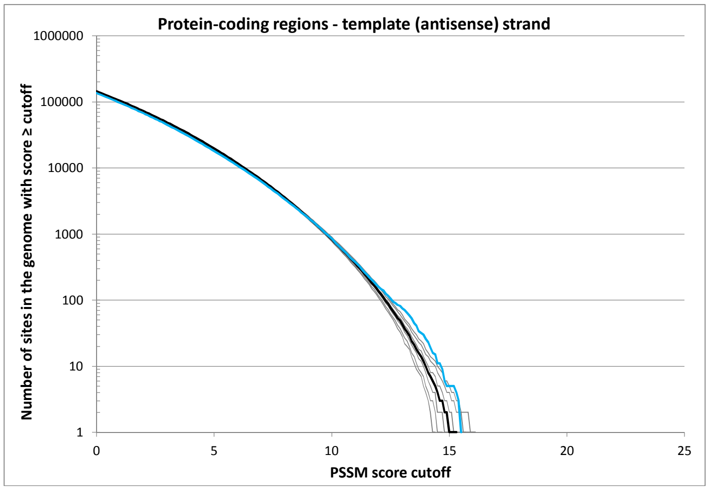

LexA (*E. coli*)

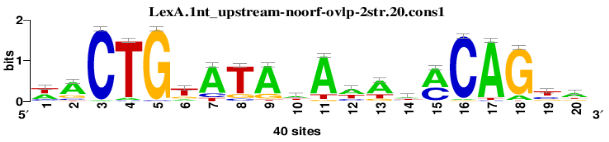

Motif length: 20
Motif information content: 17.93 bits
Max possible PSSM score: 27.92
Motif GC content: 36.12 %
Genome GC content: 50.79 %

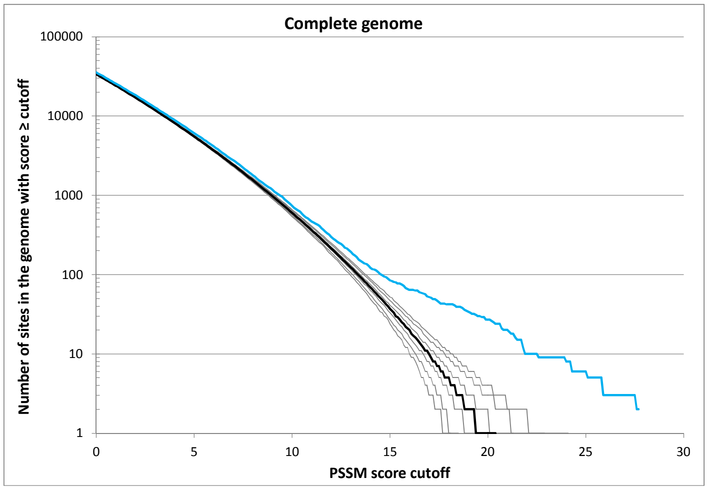

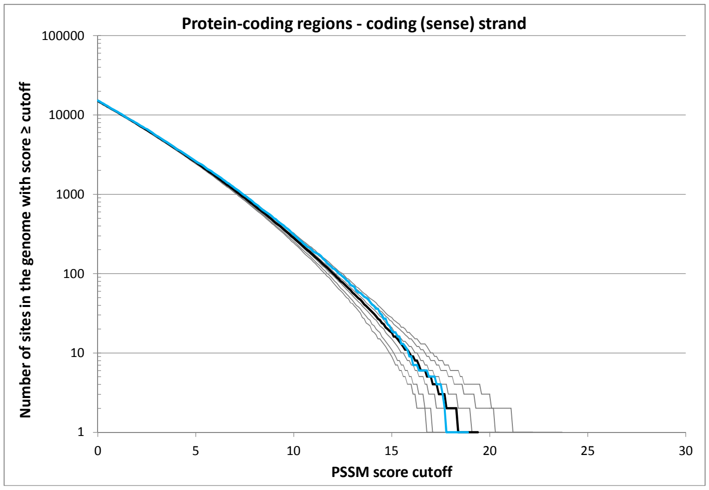

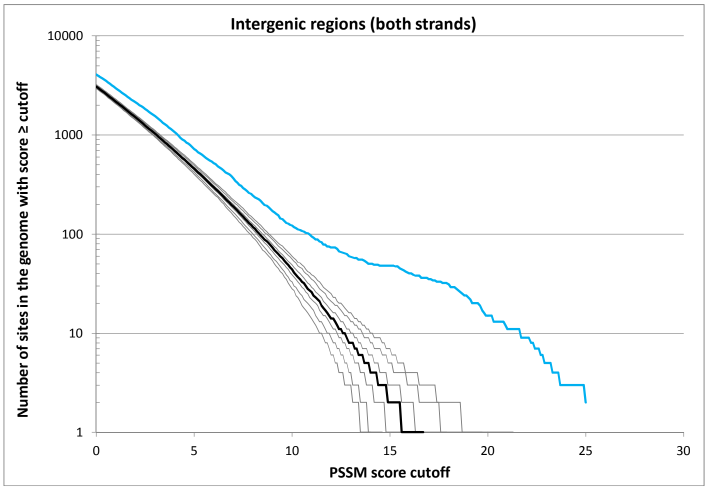

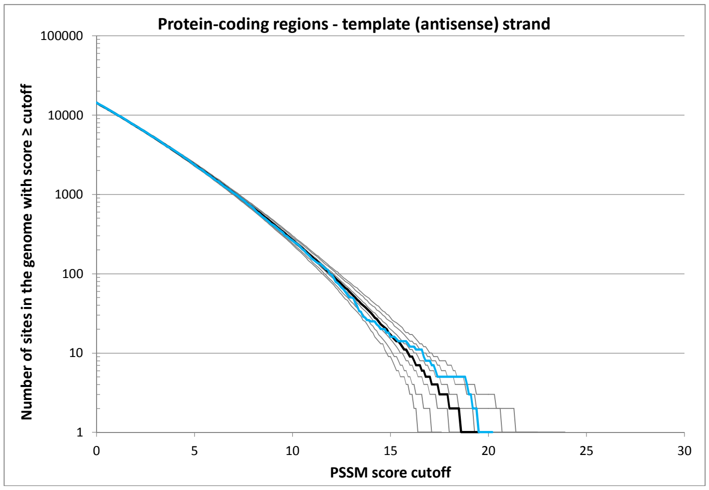

LexA (*C. difficile*)

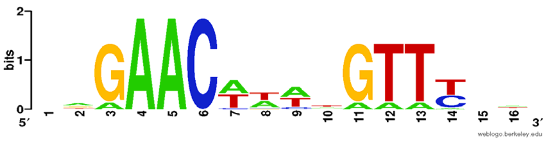

Motif length: 16
Motif information content: 15.44 bits
Max possible PSSM score: 18.31
Motif GC content: 31.62 %
Genome GC content: 28.81 %

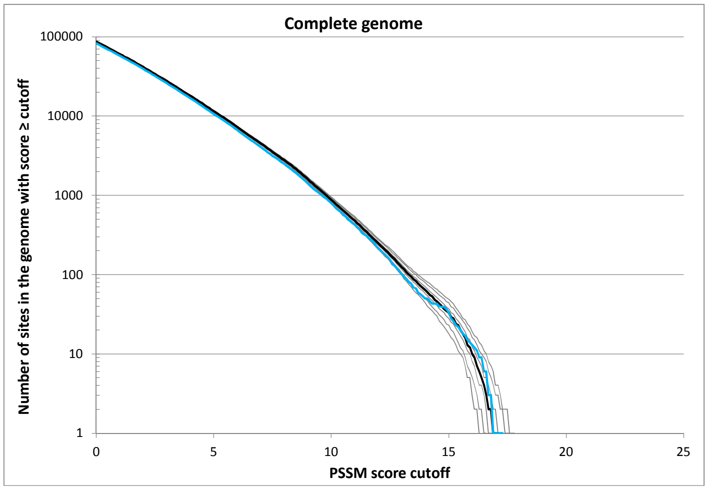

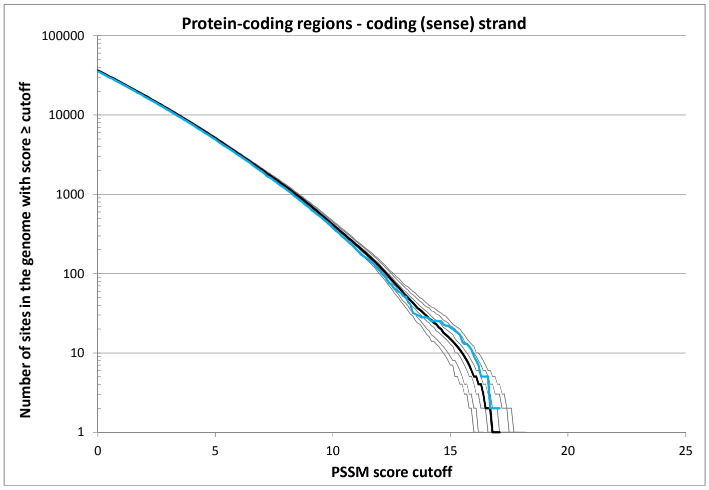

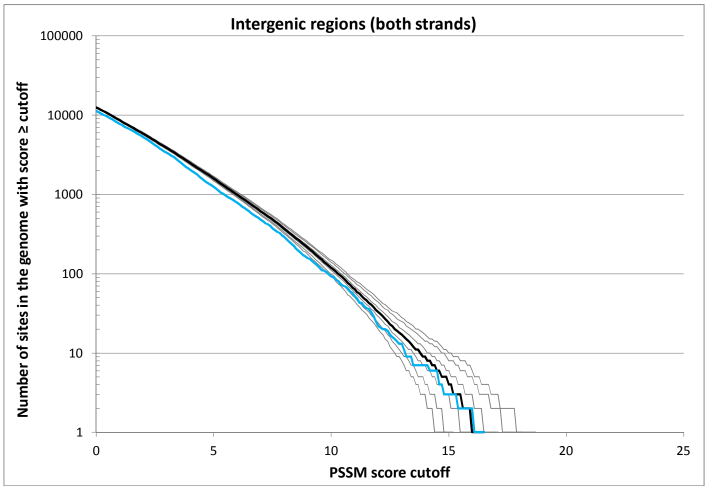

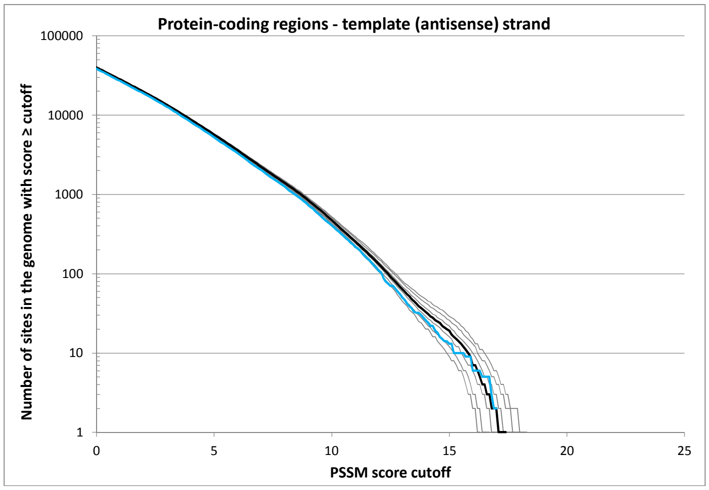

LexA (*M. tuberculosis*)

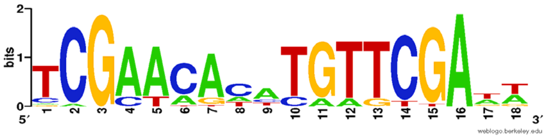

Motif length: 18
Motif information content: 22.54 bits
Max possible PSSM score: 31.87
Motif GC content: 40.58 %
Genome GC content: 65.61 %

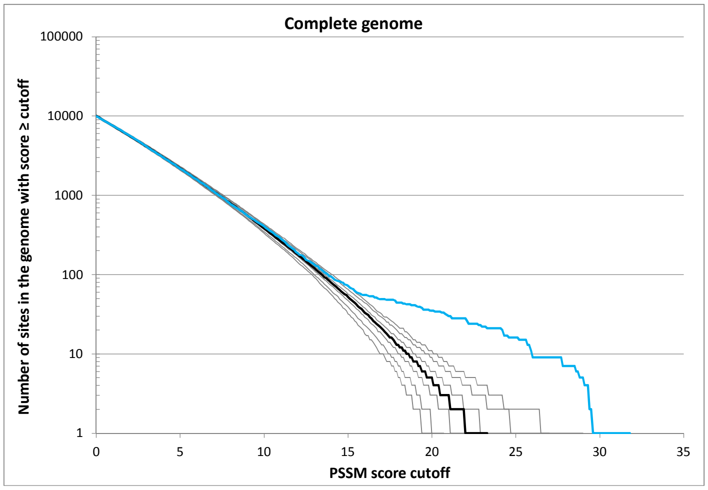

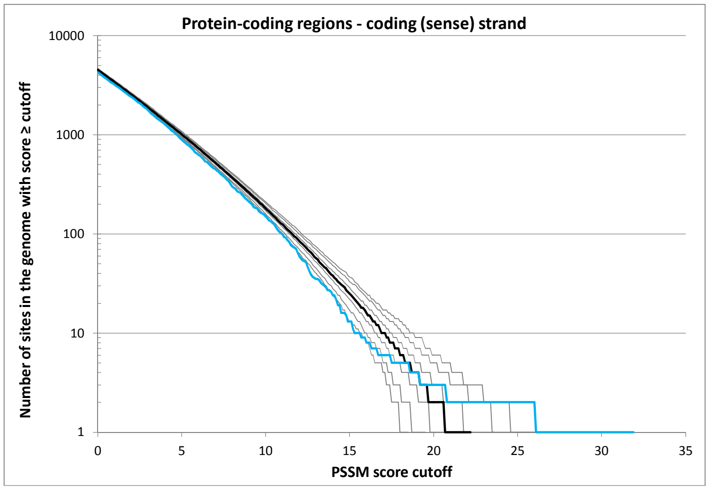

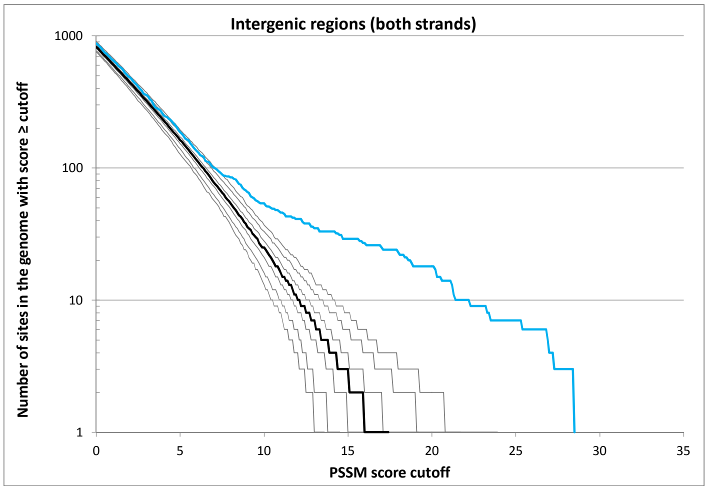

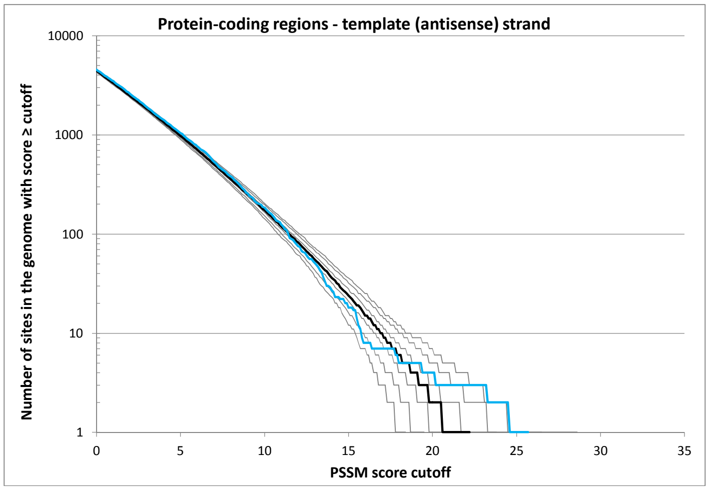

Lrp (*E. coli*)

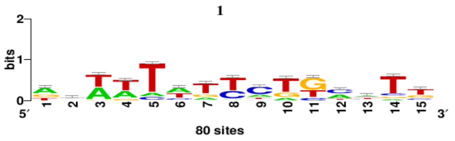

Motif length: 15
Motif information content: 7.11 bits
Max possible PSSM score: 15.96
Motif GC content: 31.75 %
Genome GC content: 50.79 %

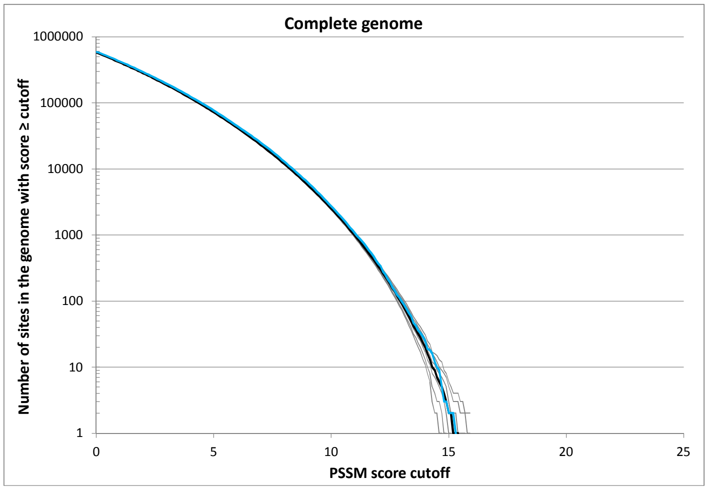

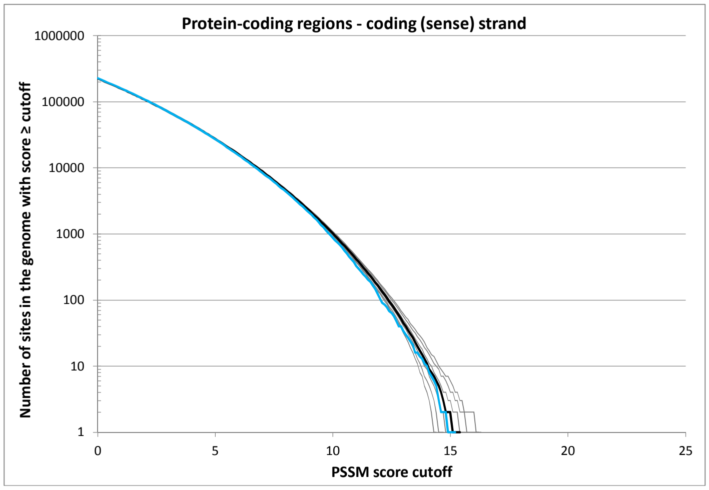

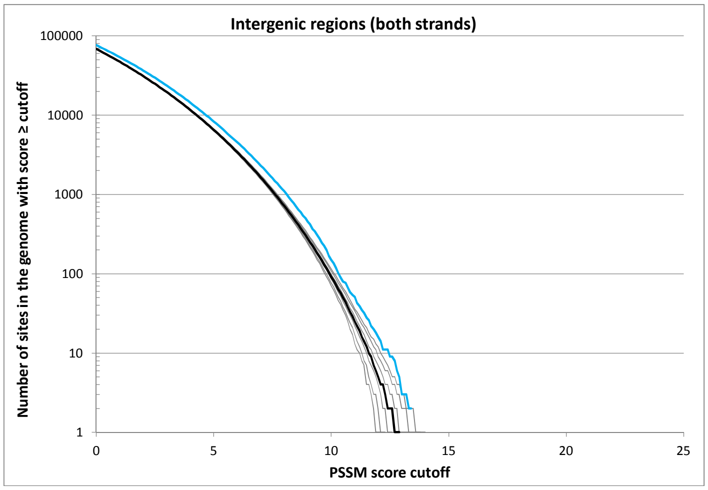

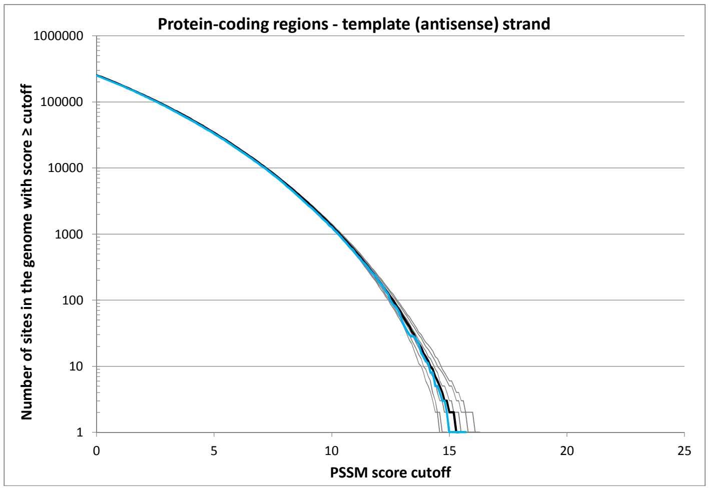

MalT (*E. coli*)

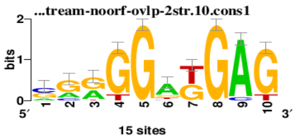

Motif length: 10
Motif information content: 11.96 bits
Max possible PSSM score: 14.37
Motif GC content: 65.33 %
Genome GC content: 50.79 %

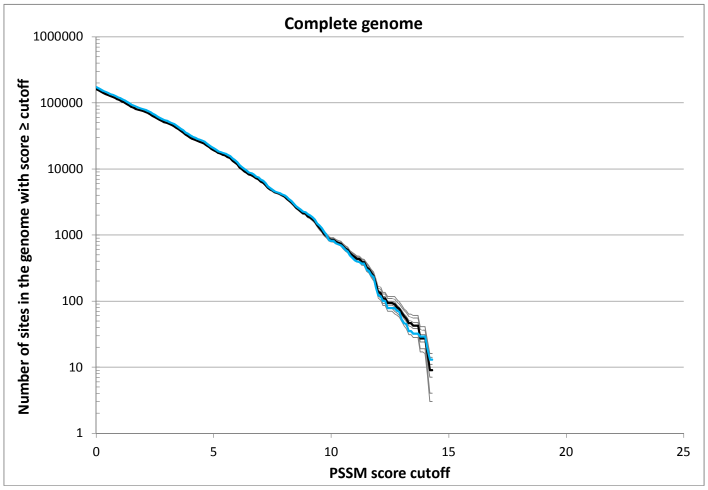

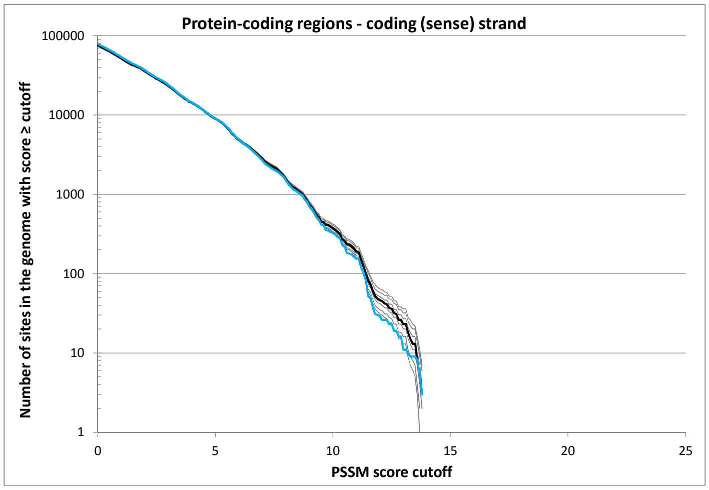

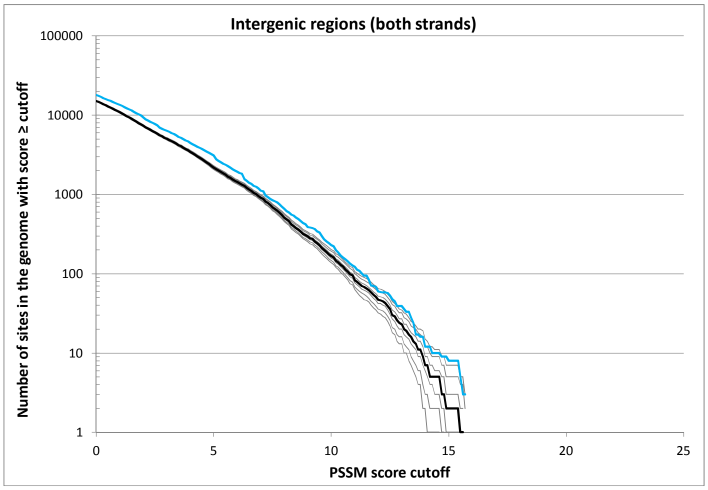

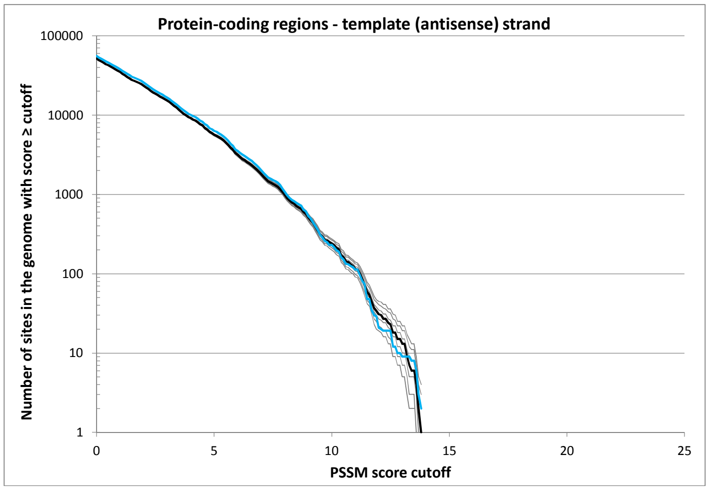

MarA (*E. coli*)

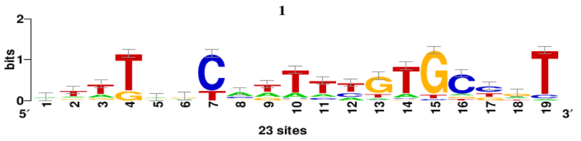

Motif length: 19
Motif information content: 12.43 bits
Max possible PSSM score: 23.20
Motif GC content: 40.50 %
Genome GC content: 50.79 %

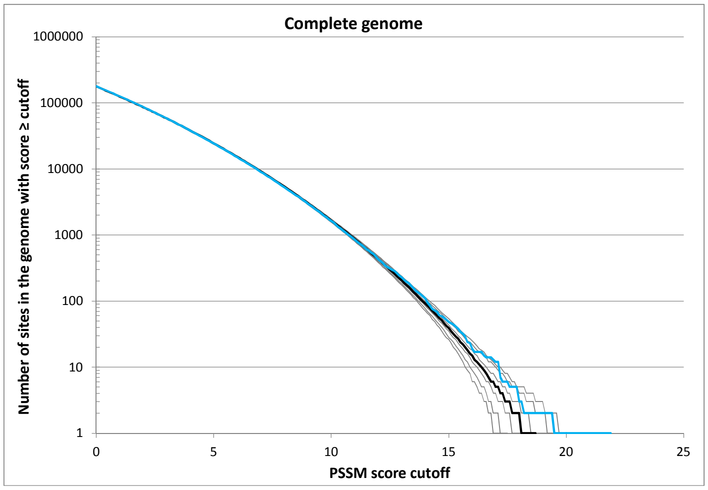

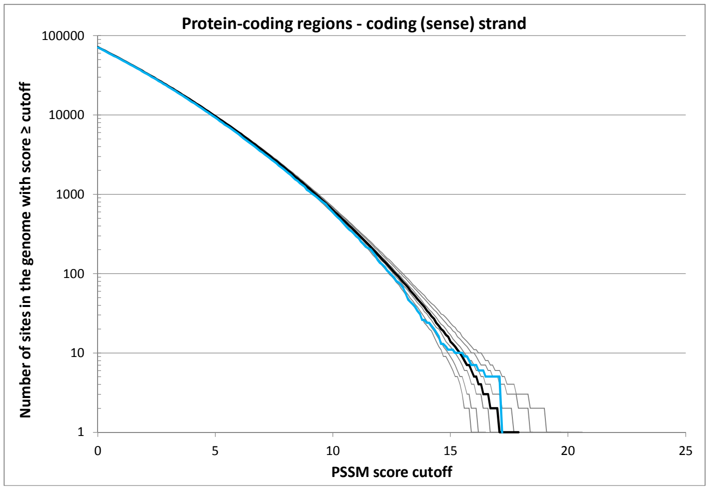

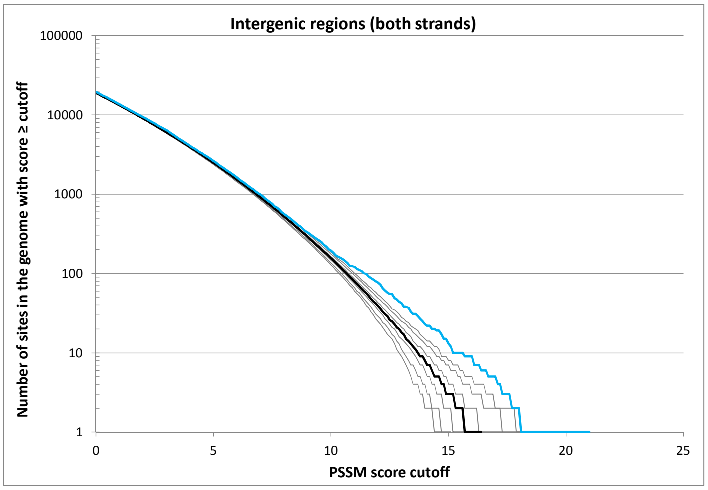

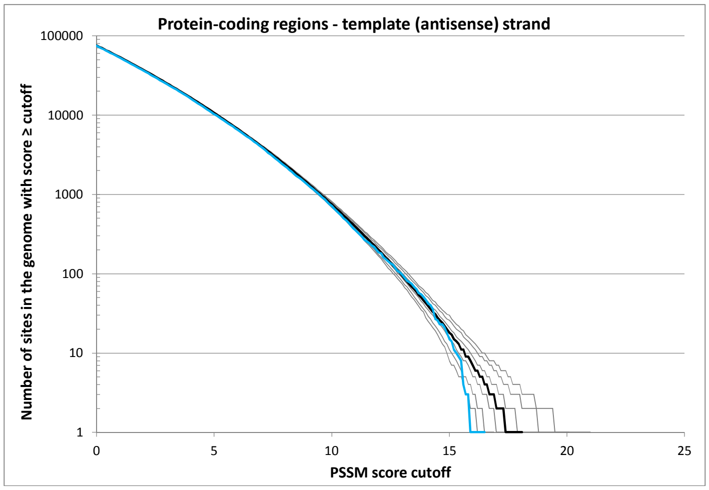

MetJ (*E. coli*)

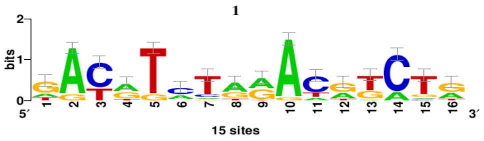

Motif length: 16
Motif information content: 14.11 bits
Max possible PSSM score: 20.96
Motif GC content: 41.67 %
Genome GC content: 50.79 %

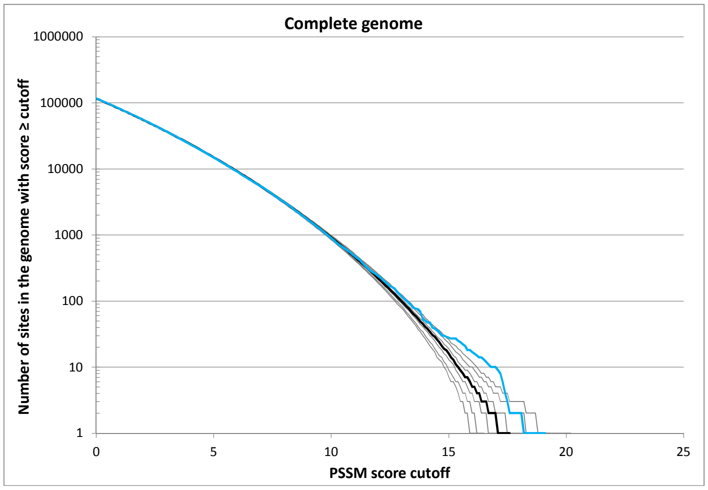

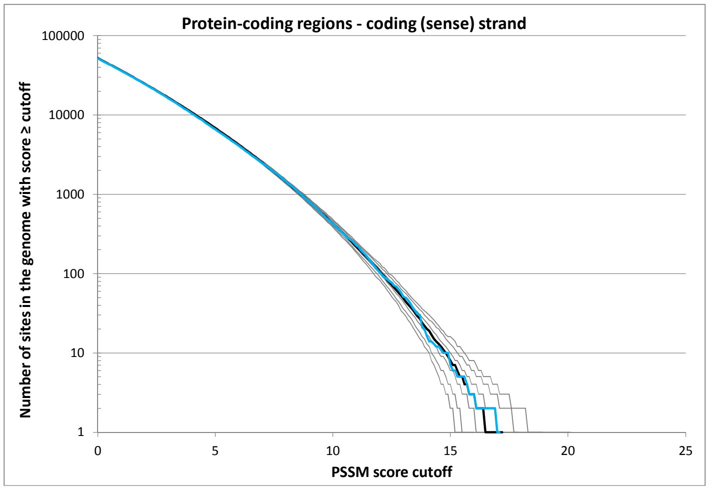

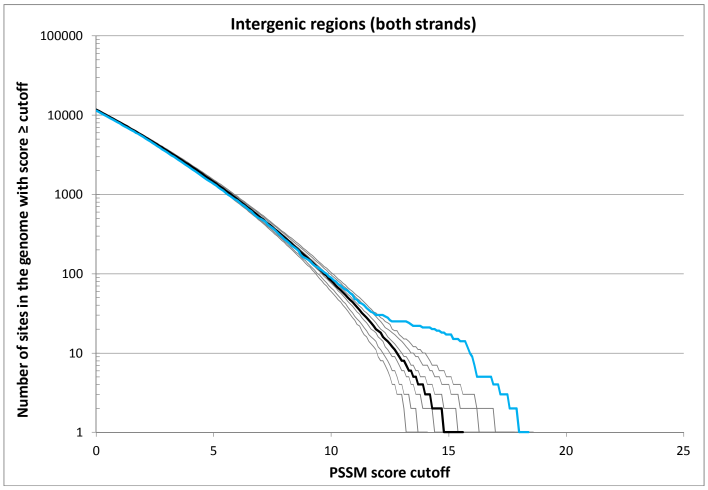

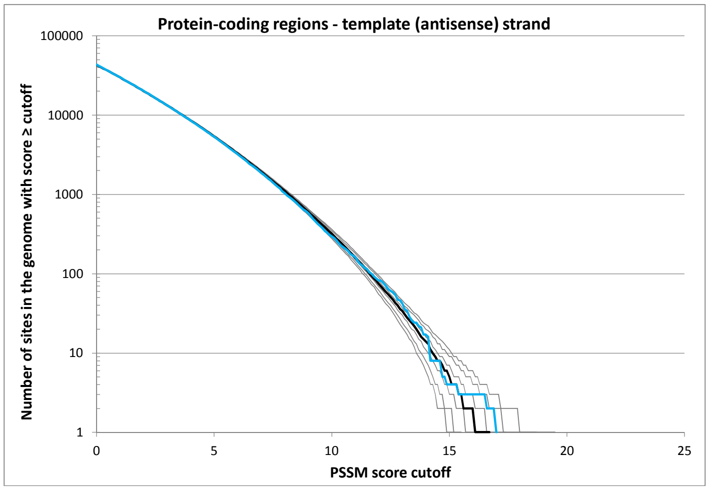

Nac (*E. coli*)

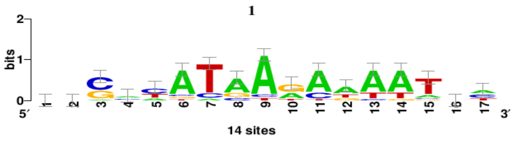

Motif length: 17
Motif information content: 10.56 bits
Max possible PSSM score: 18.63
Motif GC content: 31.51 %
Genome GC content: 50.79 %

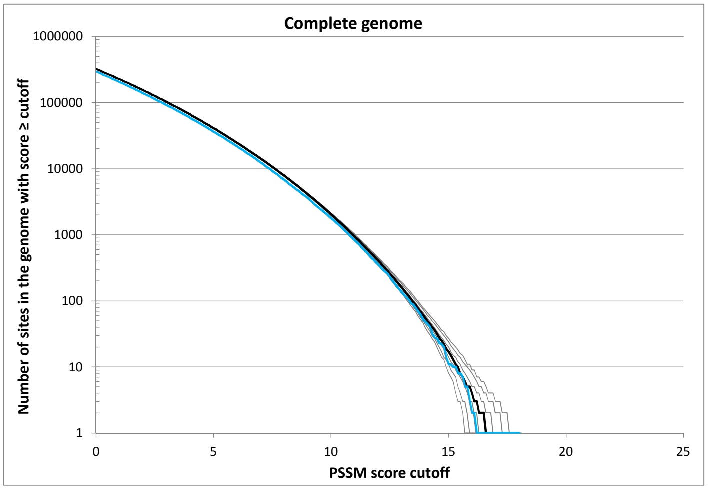

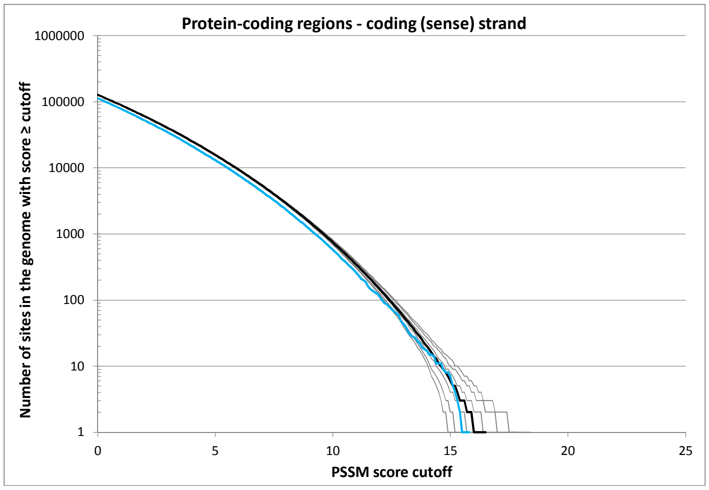

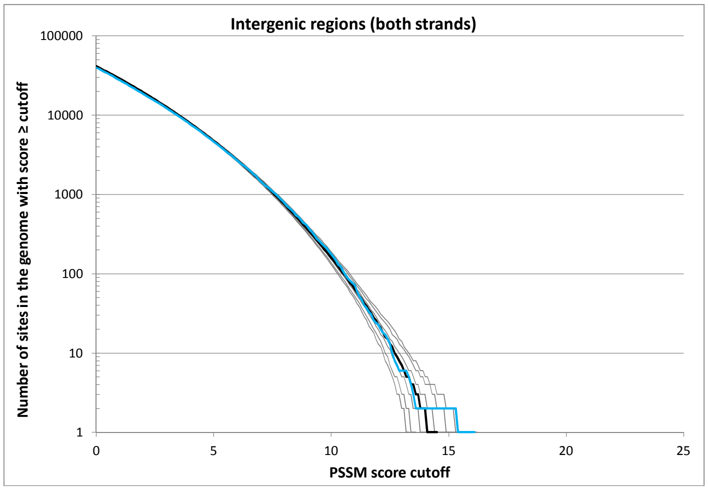

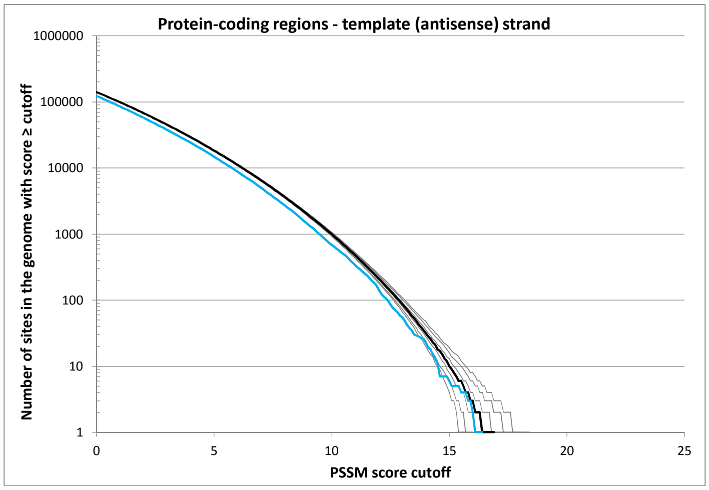

NagC (*E. coli*)

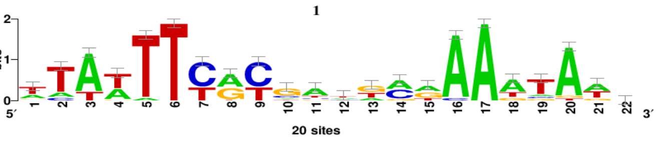

Motif length: 22
Motif information content: 19.86 bits
Max possible PSSM score: 28.05
Motif GC content: 27.50 %
Genome GC content: 50.79 %

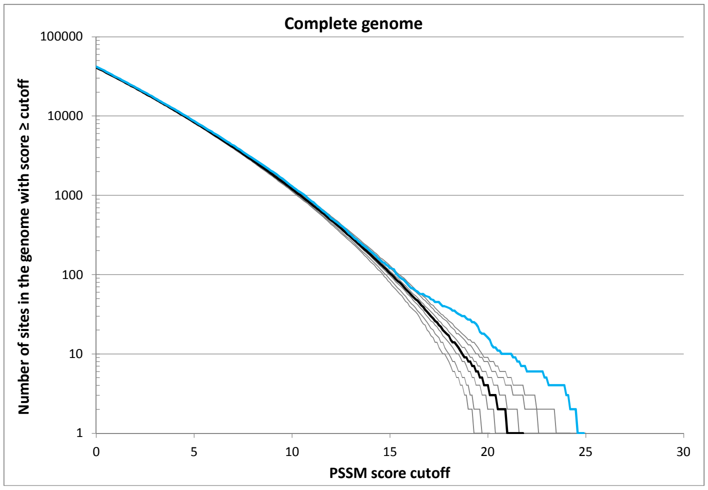

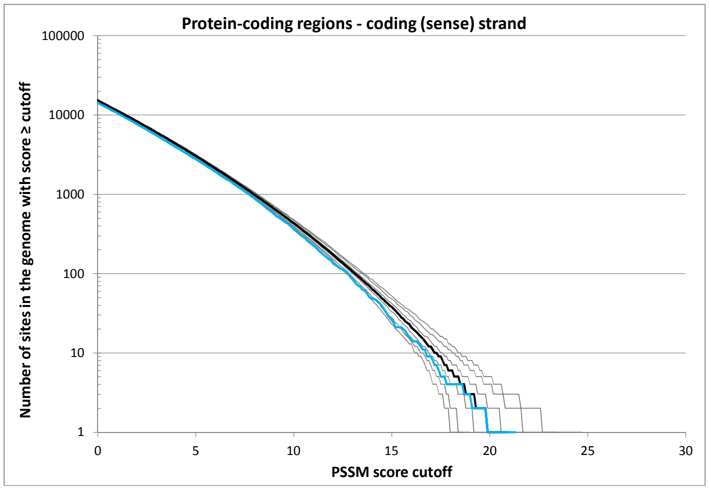

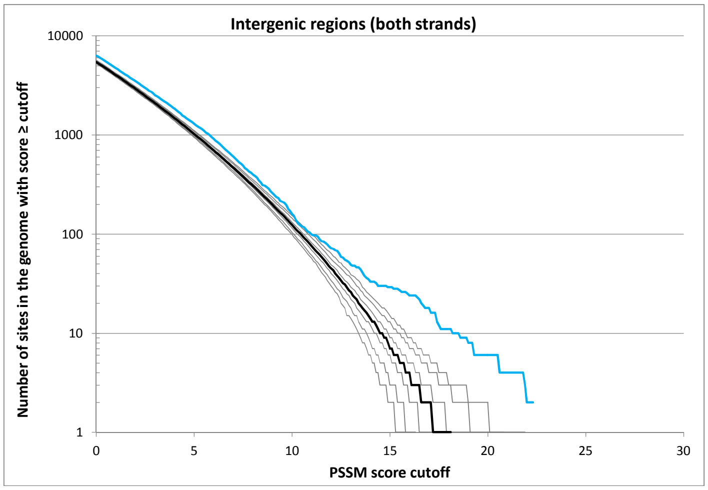

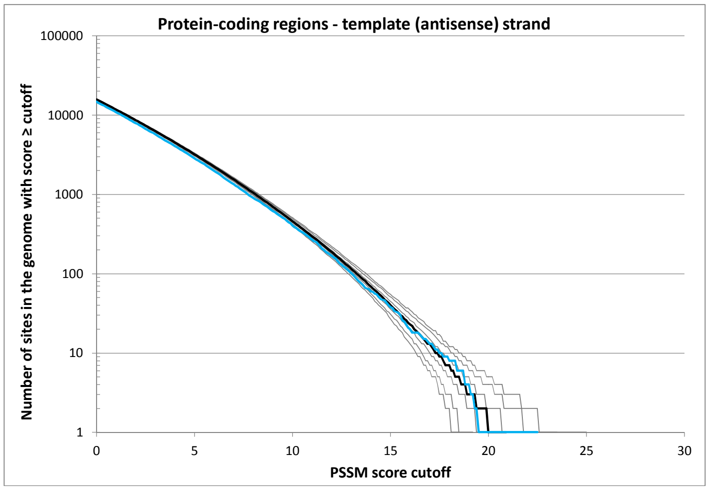

NarL (*E. coli*)

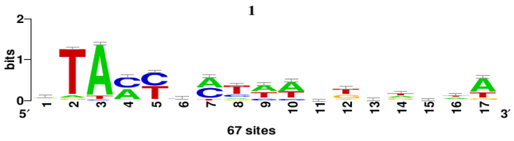

Motif length: 17
Motif information content: 7.77 bits
Max possible PSSM score: 15.43
Motif GC content: 34.42 %
Genome GC content: 50.79 %

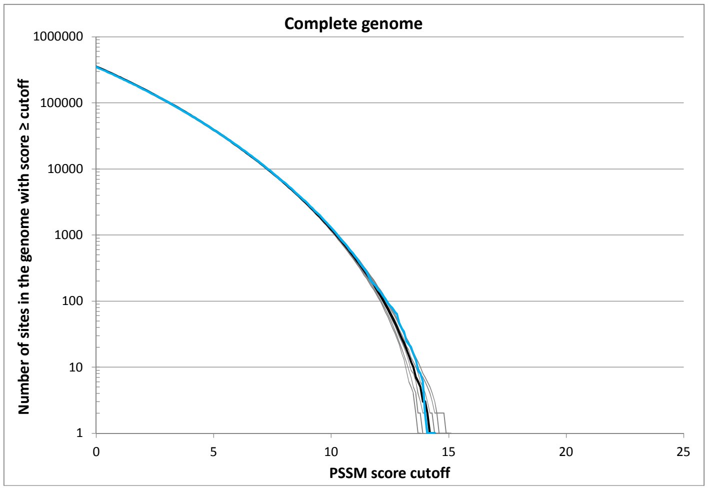

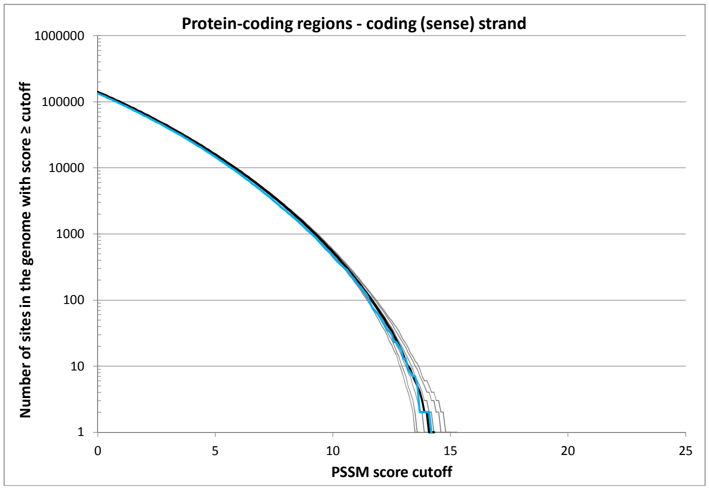

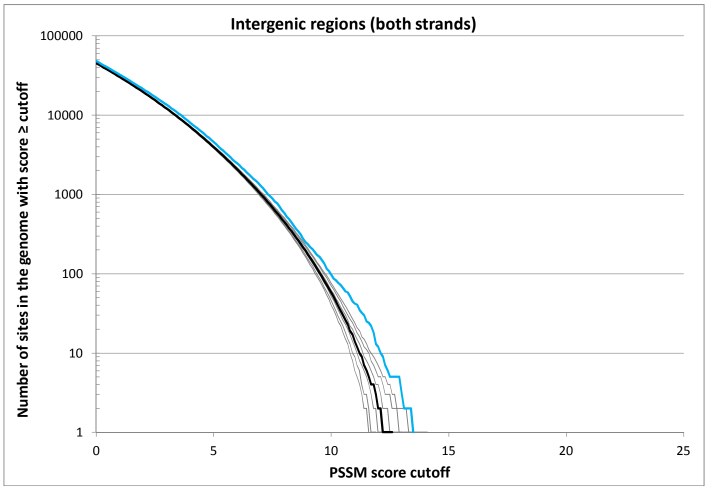

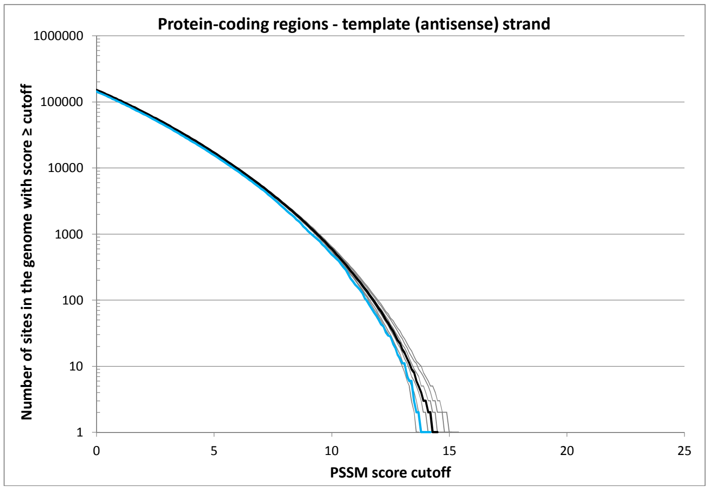

NsrR (*E. coli*)

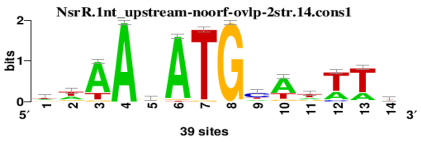

Motif length: 14
Motif information content: 12.18 bits
Max possible PSSM score: 18.64
Motif GC content: 26.74 %
Genome GC content: 50.79 %

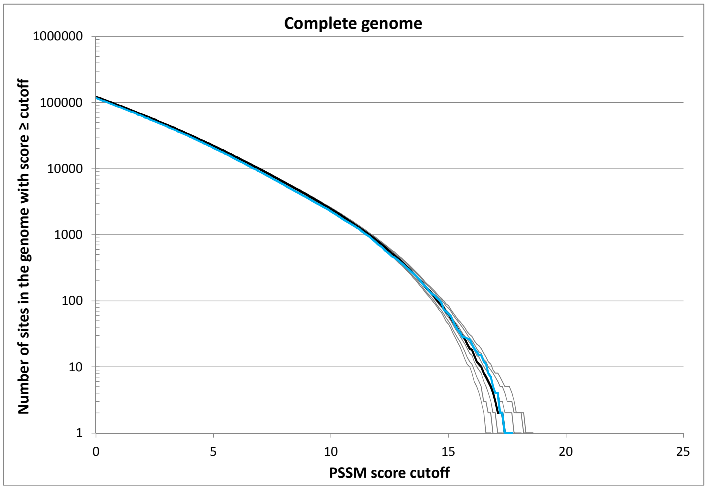

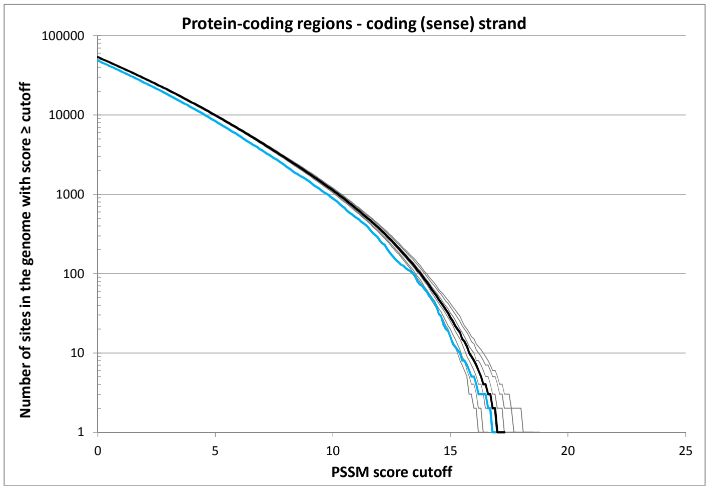

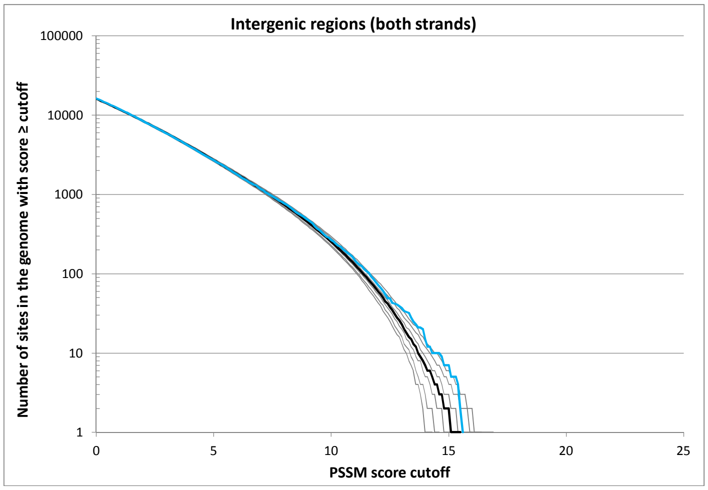

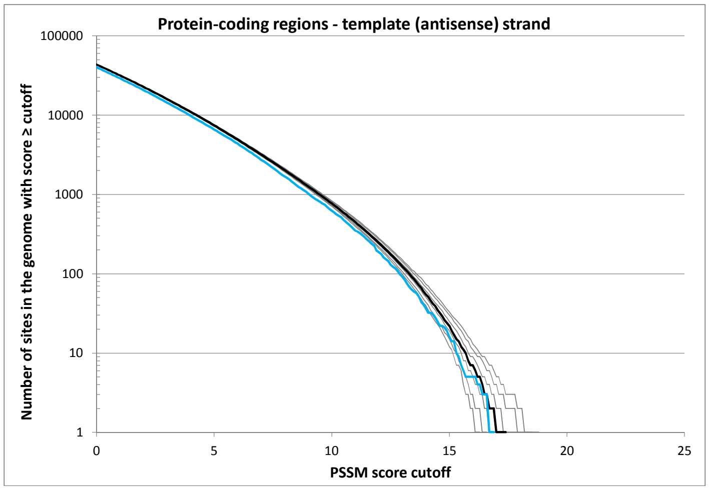

NtrC (*E. coli*)

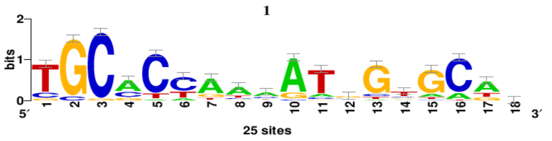

Motif length: 18
Motif information content: 14.37 bits
Max possible PSSM score: 24.98
Motif GC content: 51.33 %
Genome GC content: 50.79 %

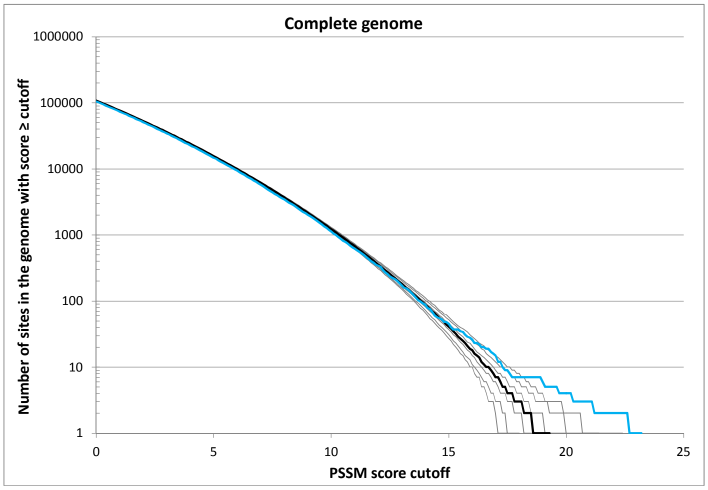

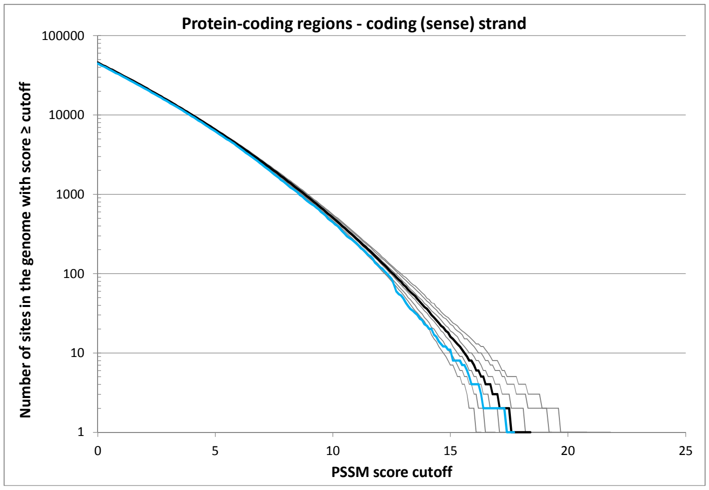

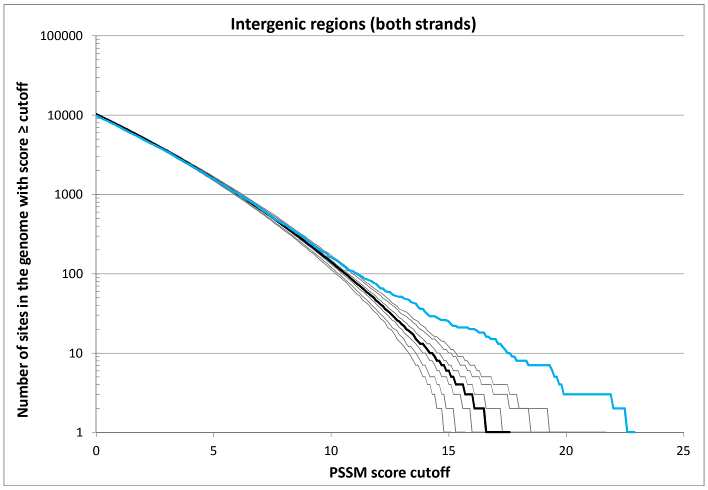

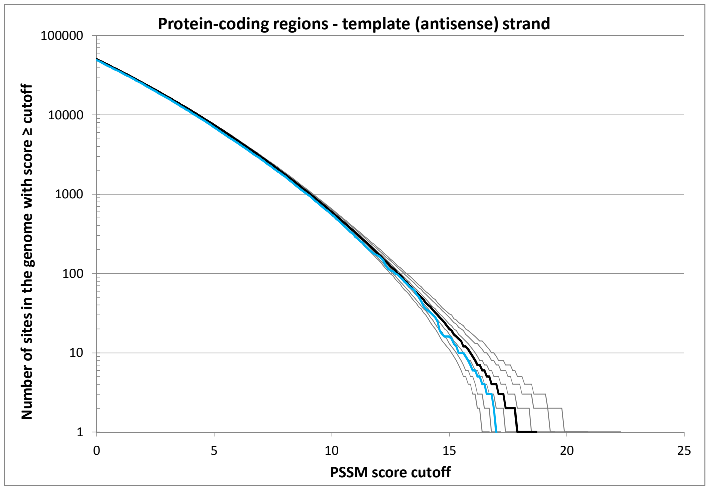

OmpR (*E. coli*)

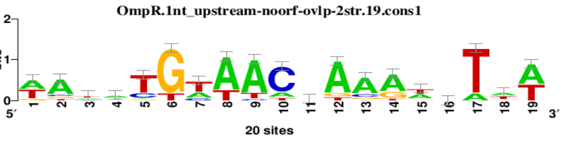

Motif length: 19
Motif information content: 13.53 bits
Max possible PSSM score: 22.72
Motif GC content: 26.05 %
Genome GC content: 50.79 %

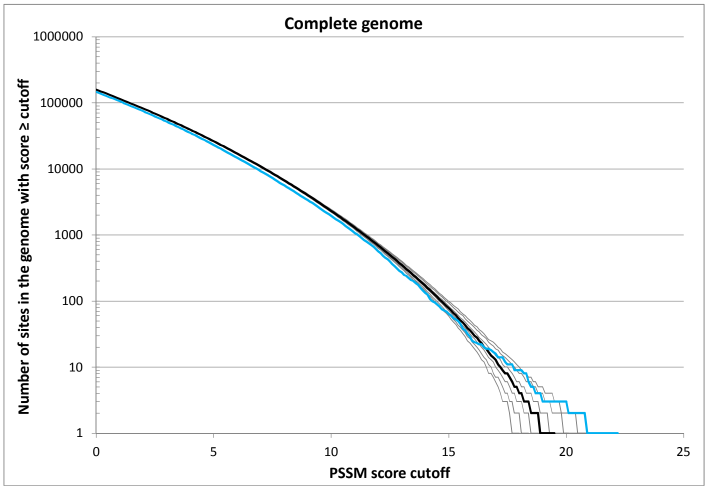

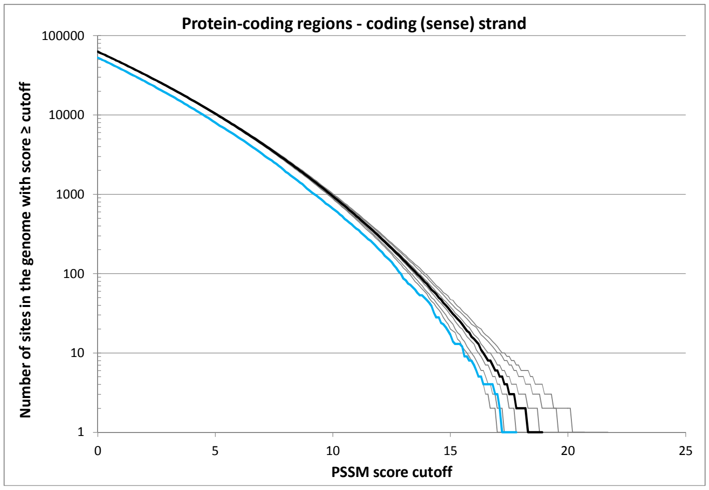

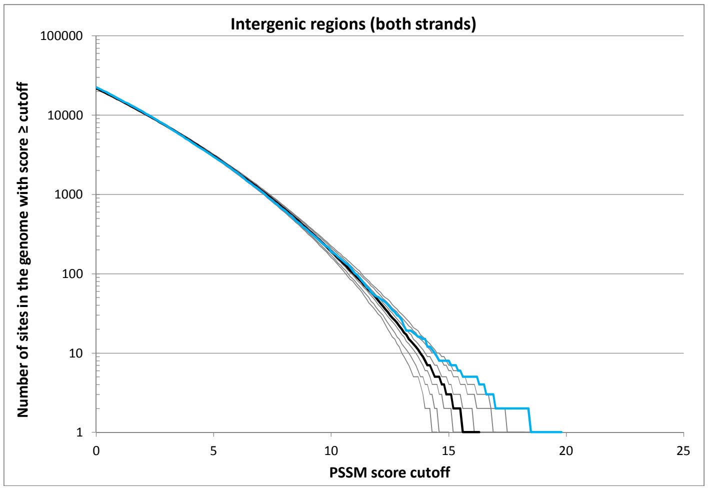

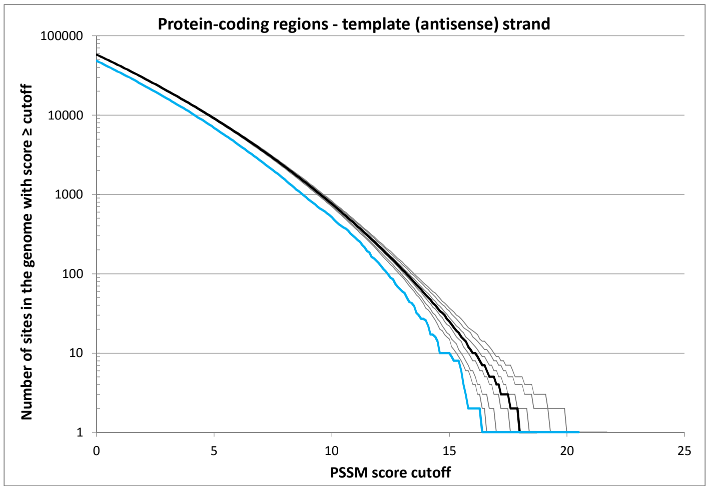

OxyR (*E. coli*)

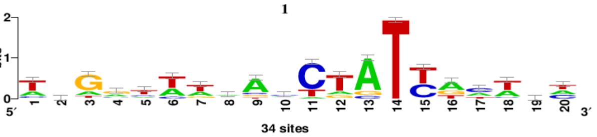

Motif length: 20
Motif information content: 11.16 bits
Max possible PSSM score: 21.28
Motif GC content: 32.94 %
Genome GC content: 50.79 %

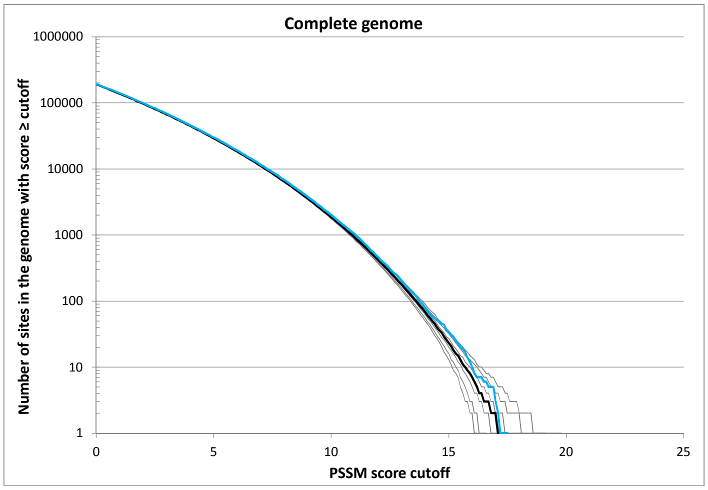

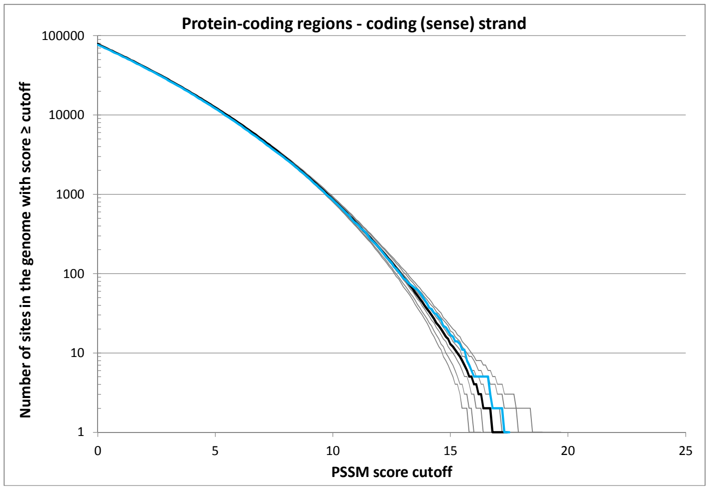

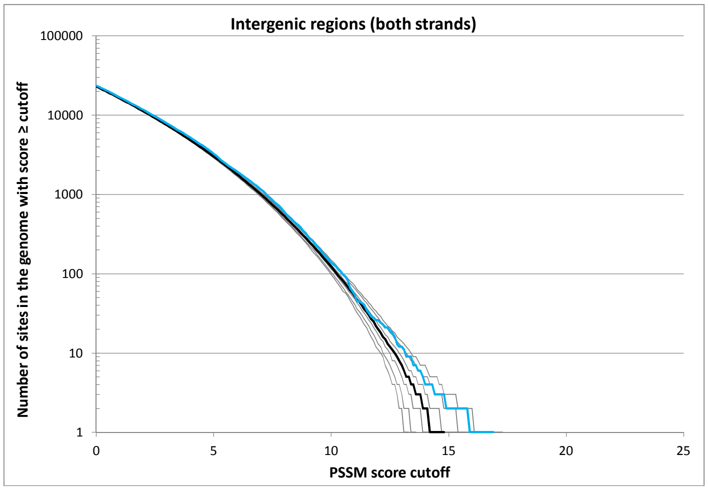

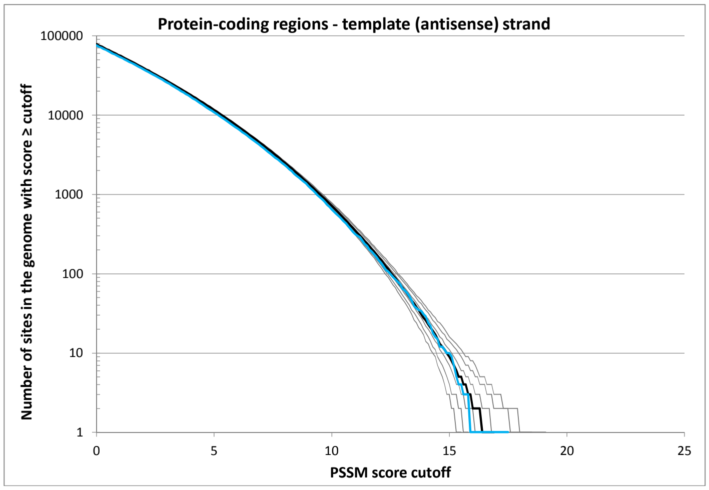

PhoB (*E. coli*)

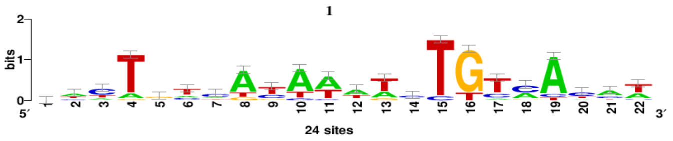

Motif length: 22
Motif information content: 13.36 bits
Max possible PSSM score: 26.04
Motif GC content: 33.33 %
Genome GC content: 50.79 %

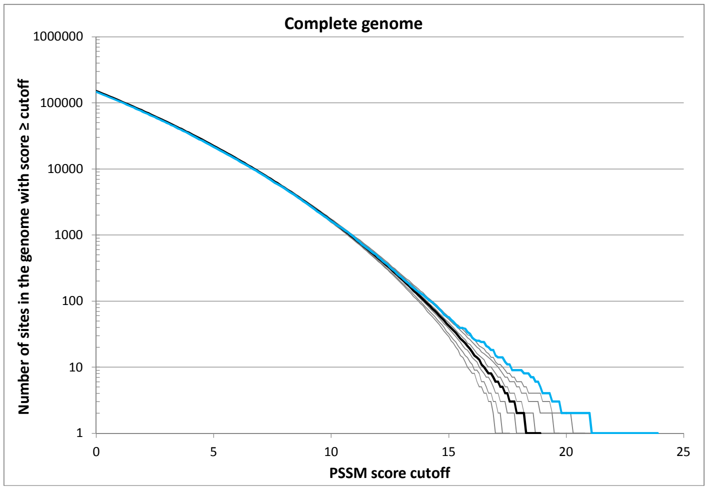

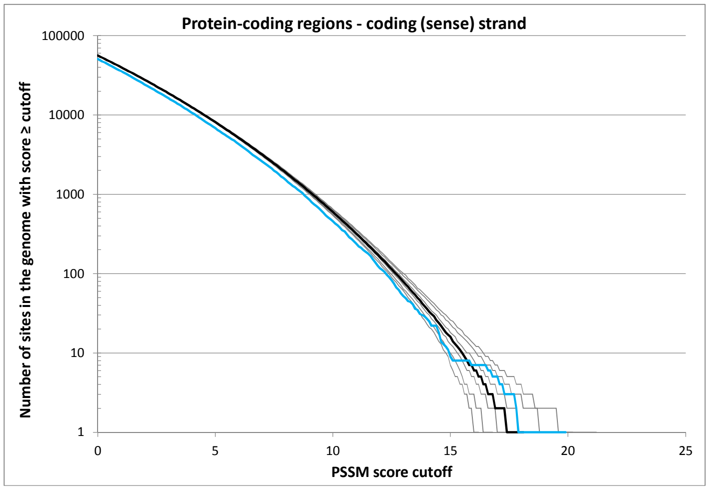

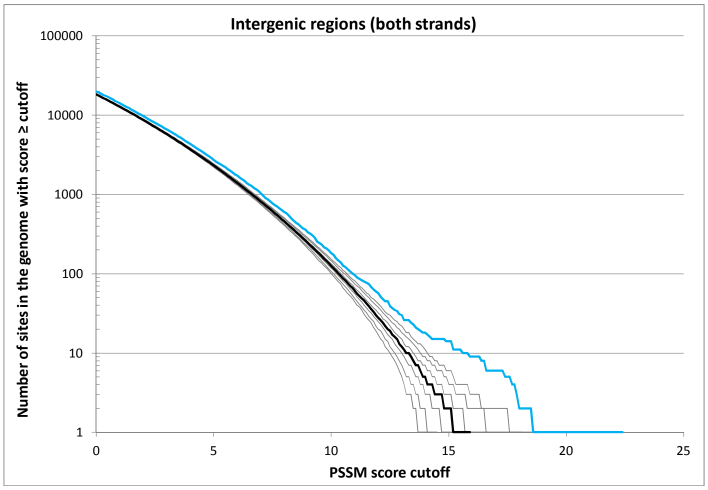

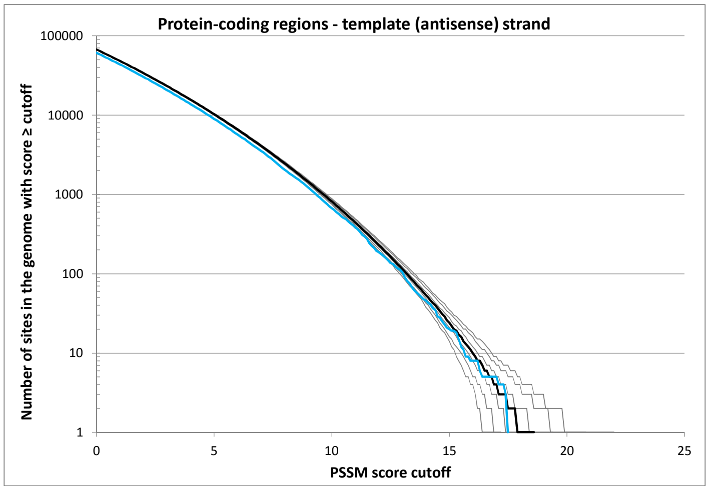

PhoP (*E. coli*)

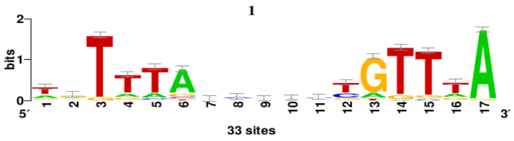

Motif length: 17
Motif information content: 12.03 bits
Max possible PSSM score: 21.55
Motif GC content: 29.95 %
Genome GC content: 50.79 %

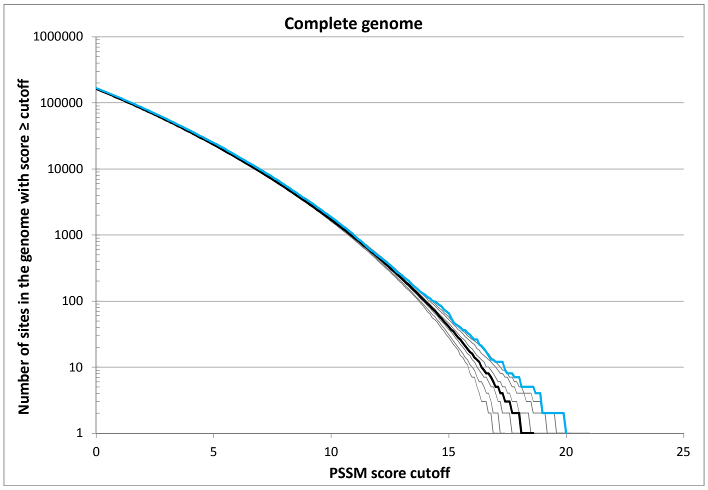

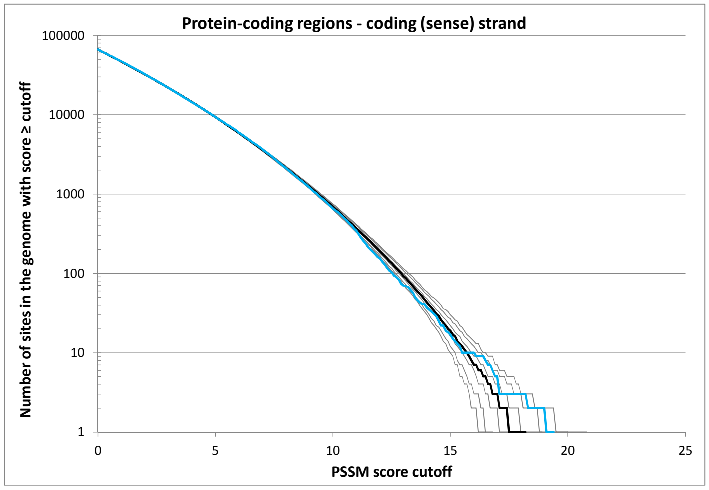

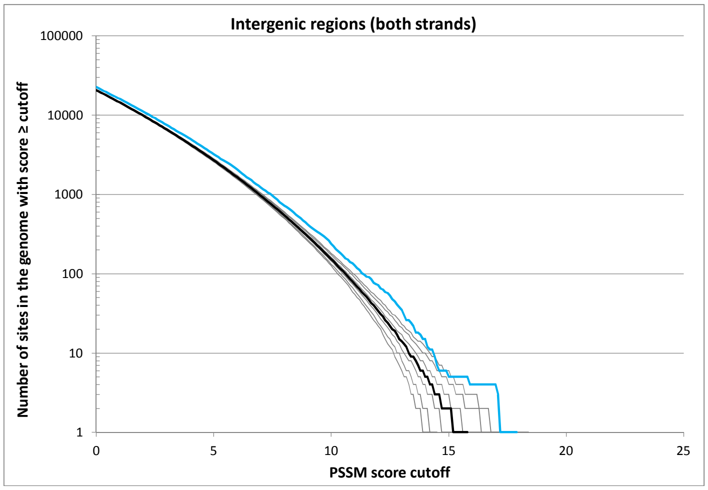

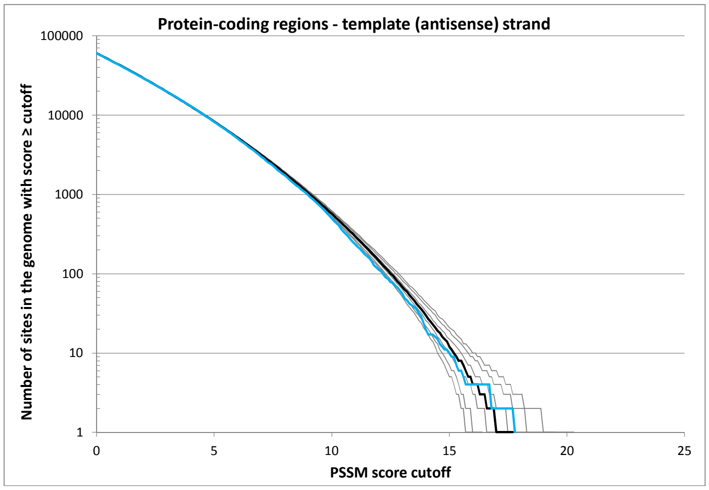

PurR (*E. coli*)

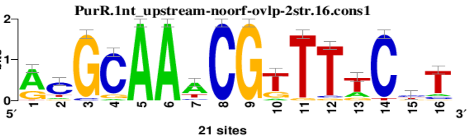

Motif length: 16
Motif information content: 20.45 bits
Max possible PSSM score: 25.83
Motif GC content: 46.13 %
Genome GC content: 50.79 %

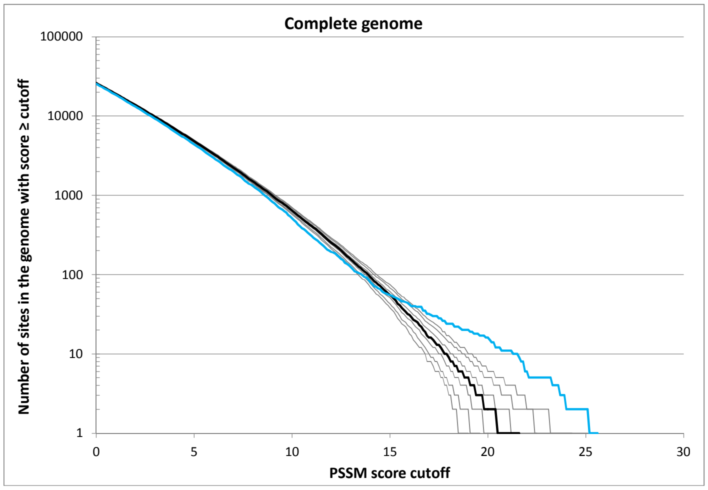

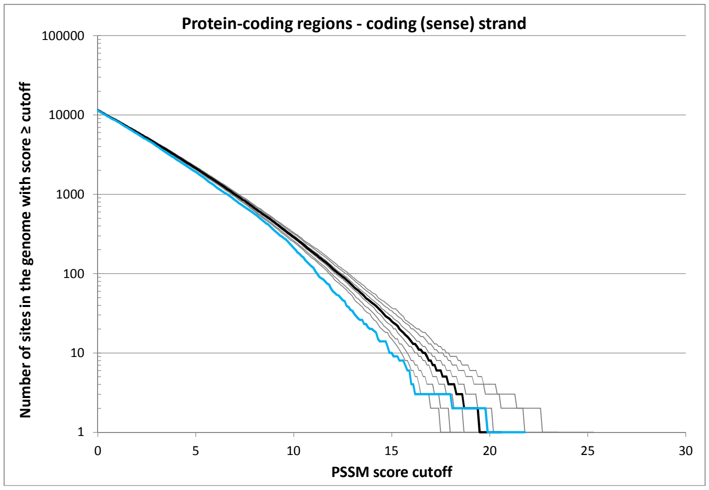

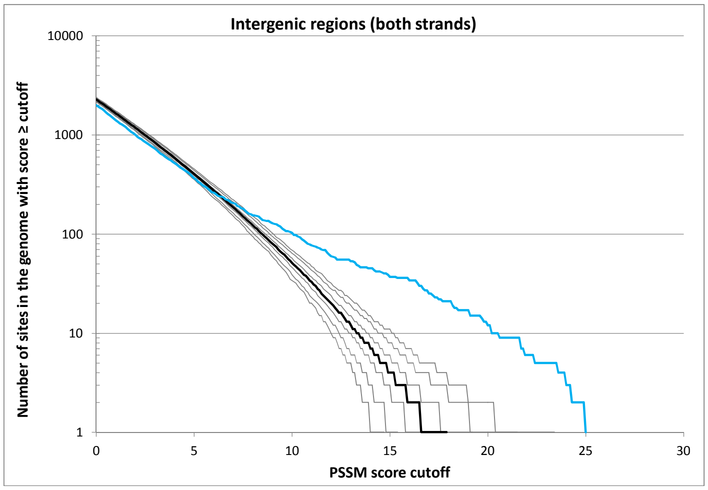

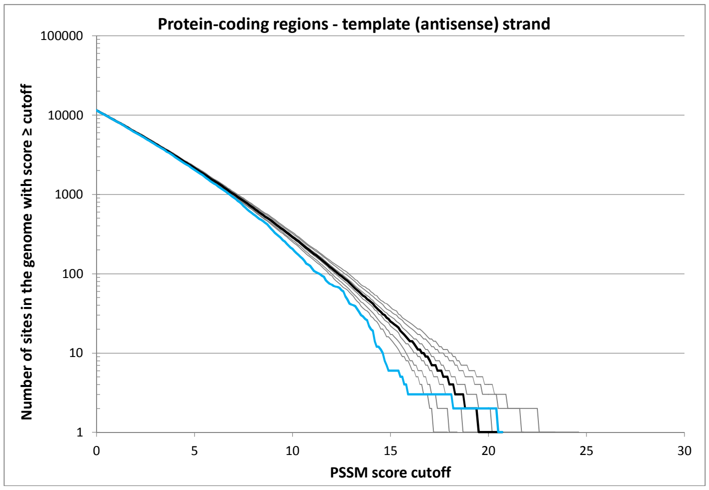

Rob (*E. coli*)

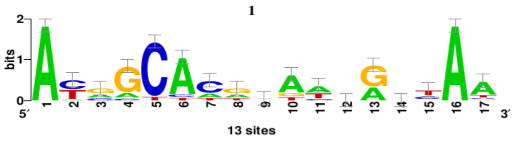

Motif length: 17
Motif information content: 14.17 bits
Max possible PSSM score: 20.63
Motif GC content: 40.27 %
Genome GC content: 50.79 %

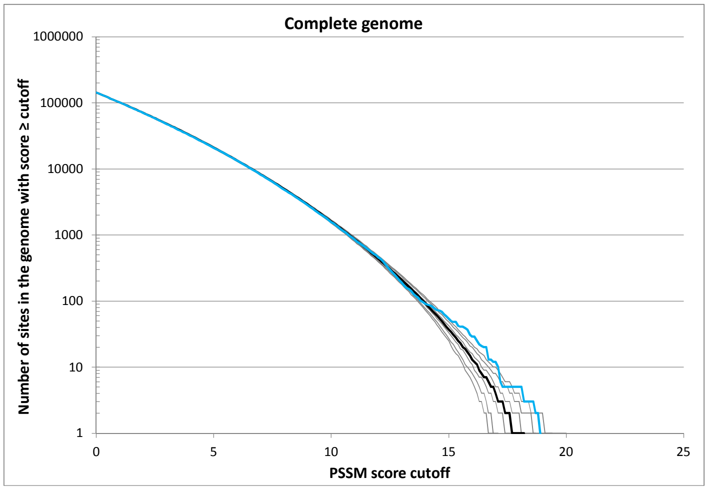

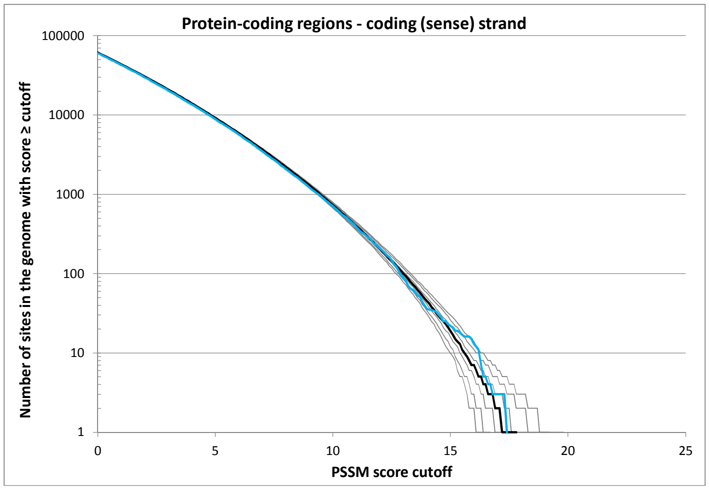

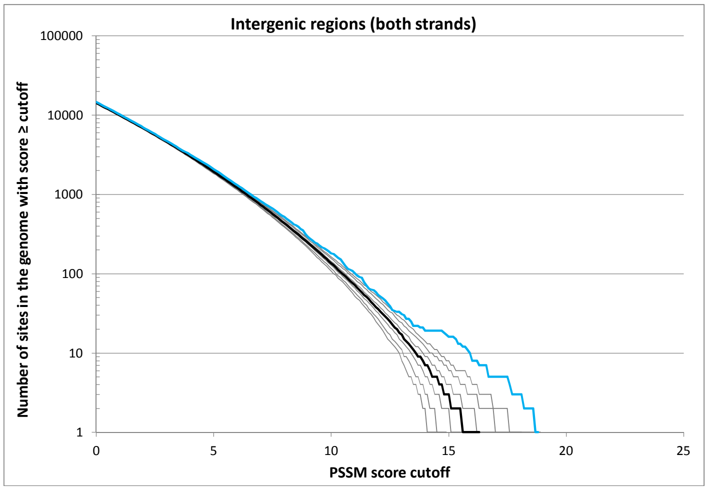

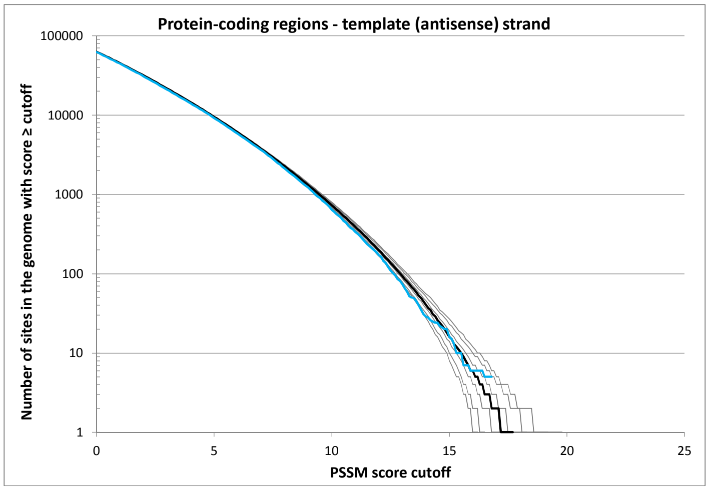

SoxS (*E. coli*)

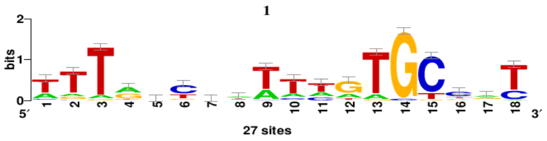

Motif length: 18
Motif information content: 12.54 bits
Max possible PSSM score: 22.15
Motif GC content: 39.30 %
Genome GC content: 50.79 %

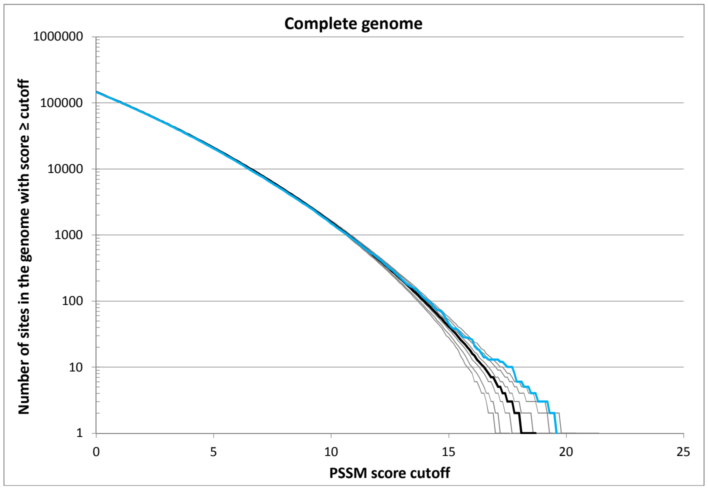

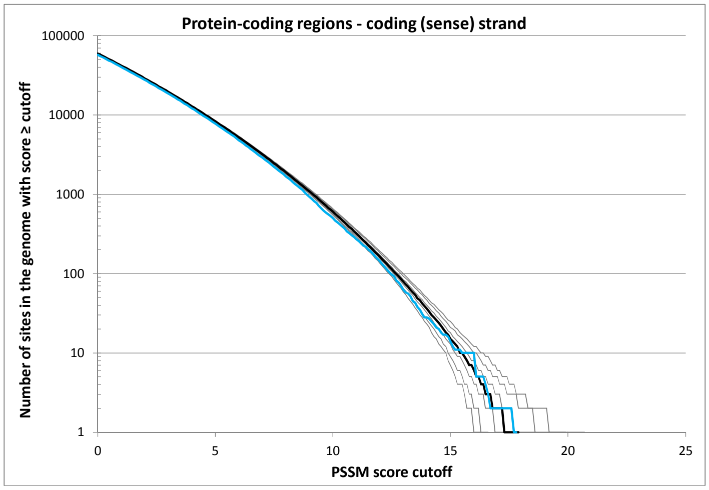

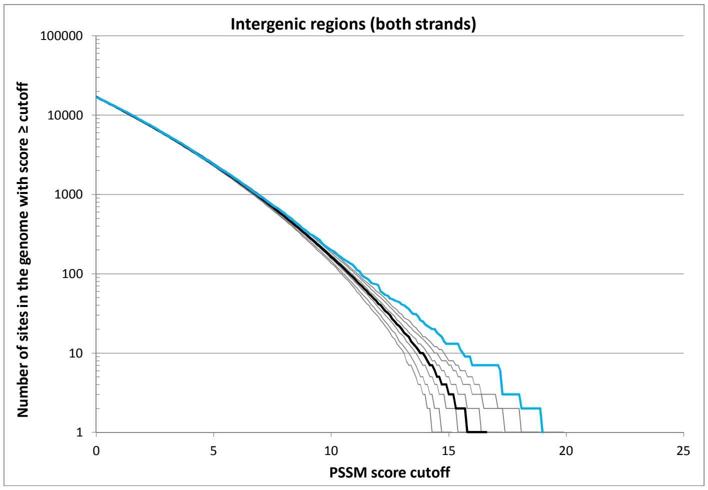

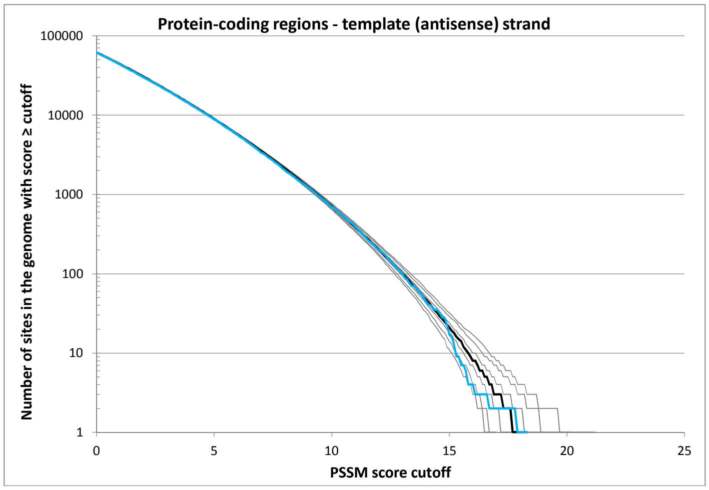

TyrR (*E. coli*)

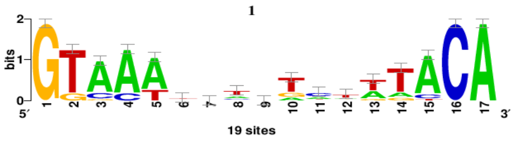

Motif length: 17
Motif information content: 15.77 bits
Max possible PSSM score: 22.87
Motif GC content: 30.65 %
Genome GC content: 50.79 %

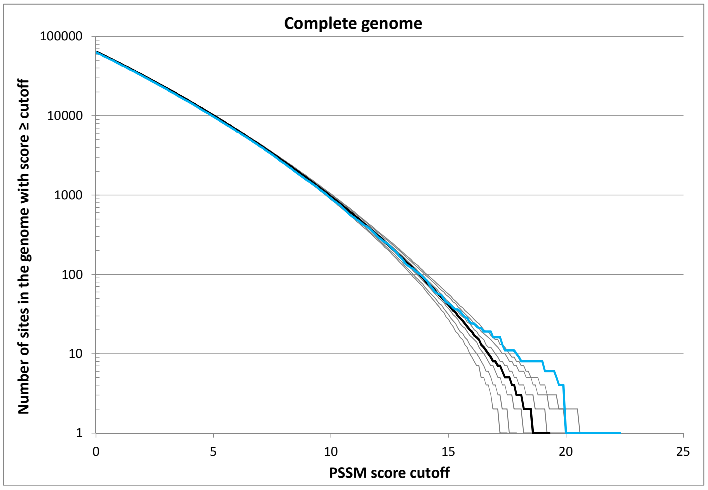

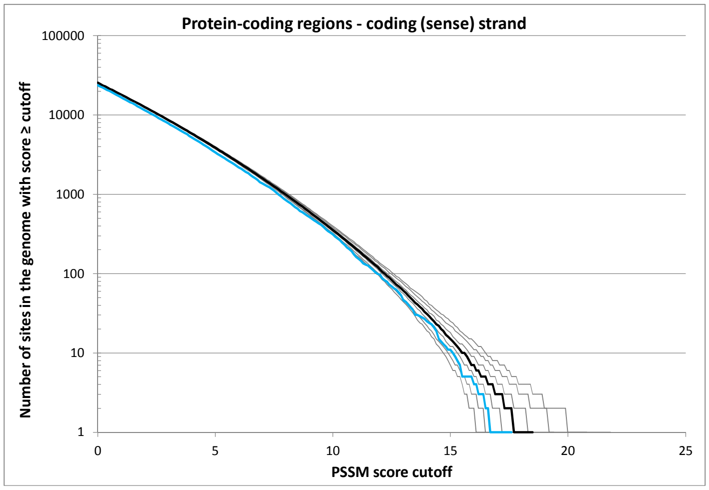

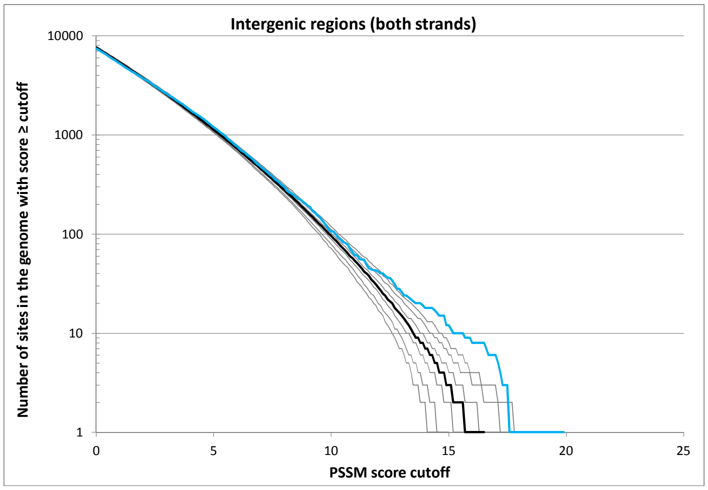

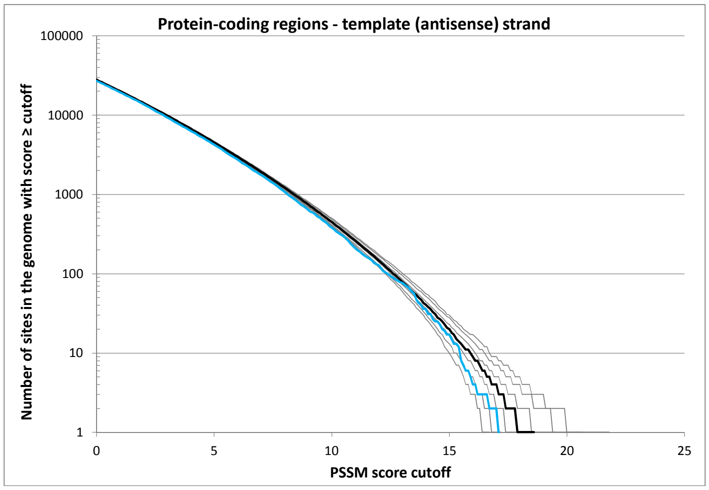

σ[54] (*S. enterica* subsp. Typhimurium)

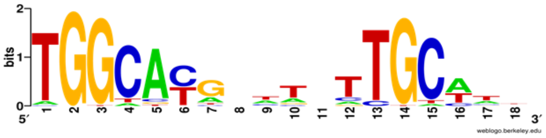

Motif length: 18
Motif information content: 16.88 bits
Max possible PSSM score: 25.00
Motif GC content: 46.12 %
Genome GC content: 52.20 %

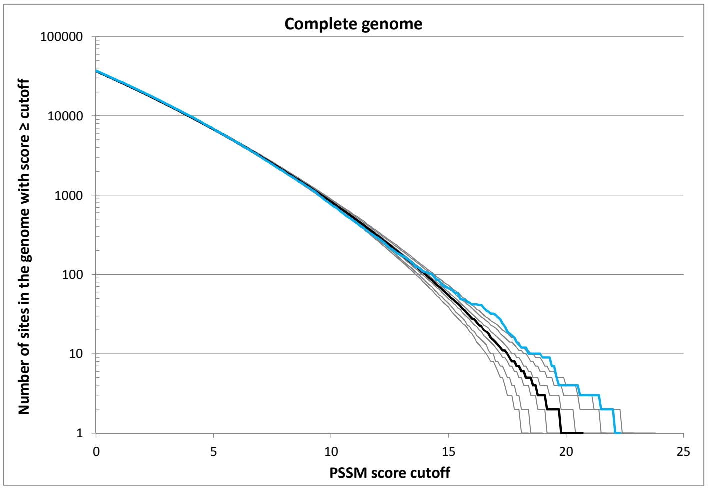

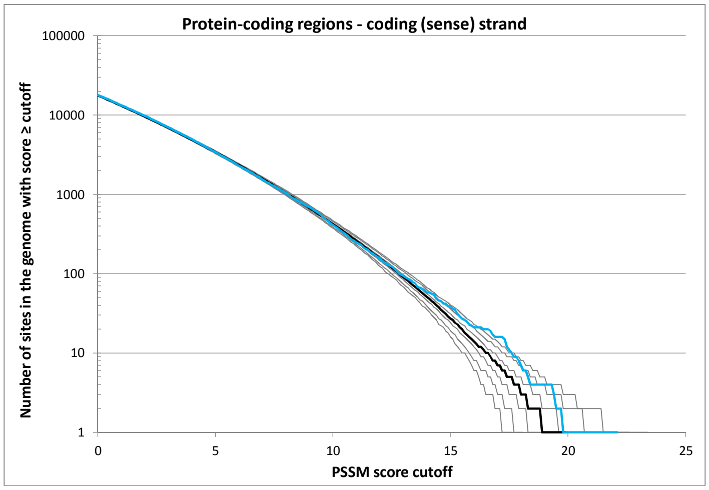

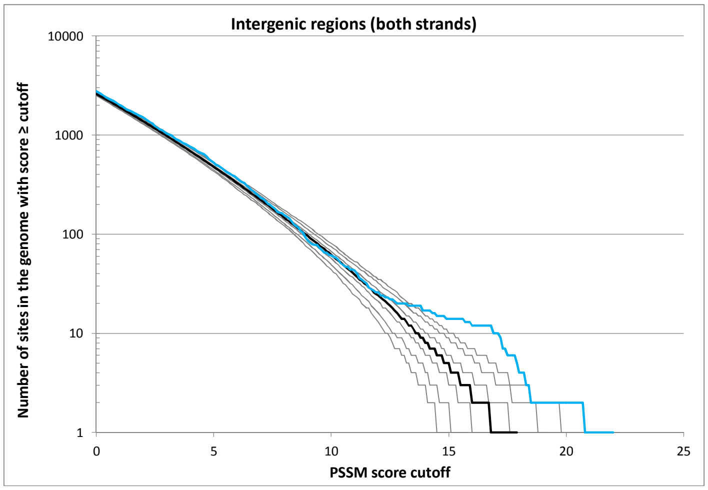

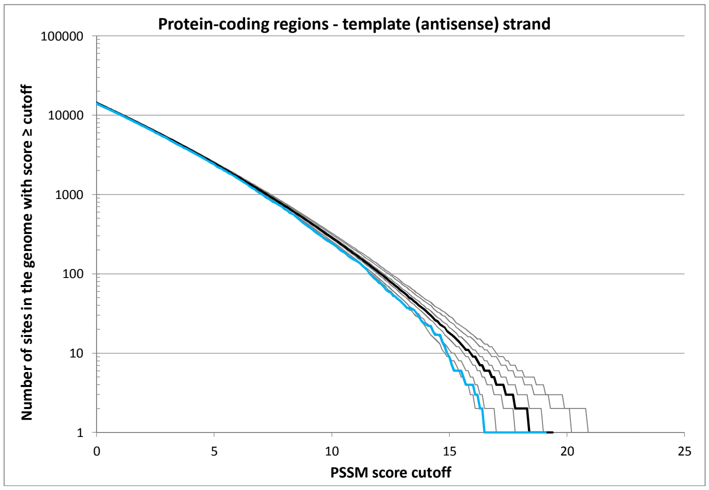

Supplement: Supplementary file 1 — Figure S1. Complete set of ‘report cards’ for transcription factor binding sites investigaed in this work. (PDF 3337 kb) [file 12862_2019_1381_MOESM1_ESM.pdf]
