# Supplementary material for: In silico simulations of occurrence of transcription factor binding sites in bacterial genomes
Source: BMC Evol Biol. 2019 Mar 1;19:67. doi: 10.1186/s12862-019-1381-8 (PMC6397444; doi:10.1186/s12862-019-1381-8)

Test: *E. coli* LexA in *C. difficile* genome

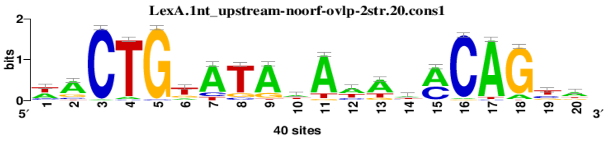

Motif length: 20
Motif information content: 17.93 bits
Max possible PSSM score: 24.65
Motif GC content: 36.12 %
Genome GC content: 28.81 %

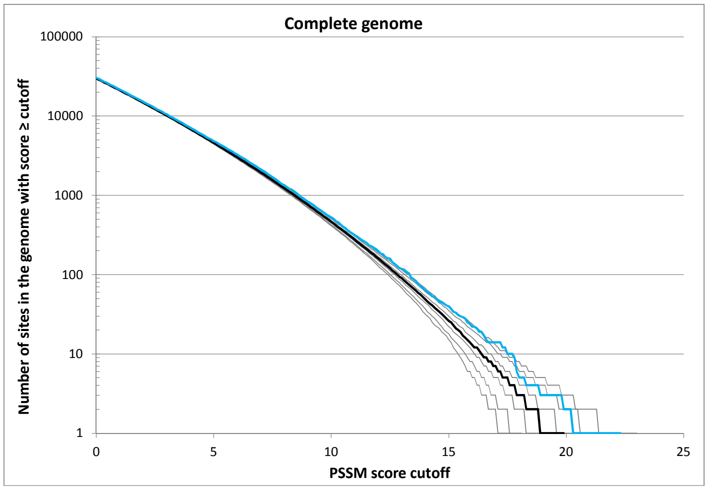

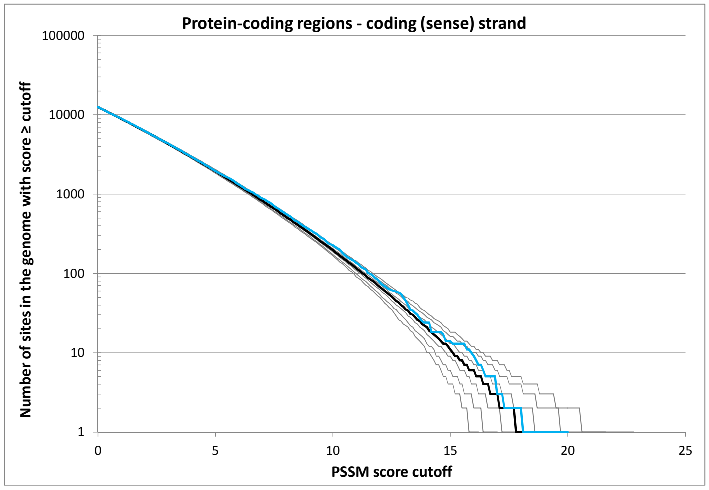

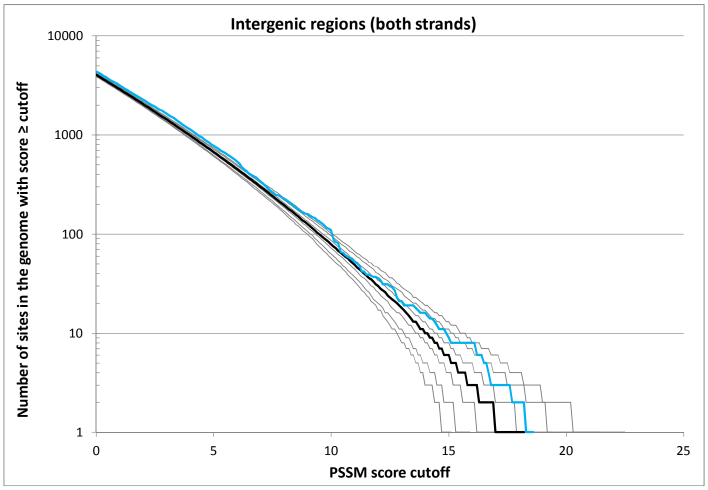

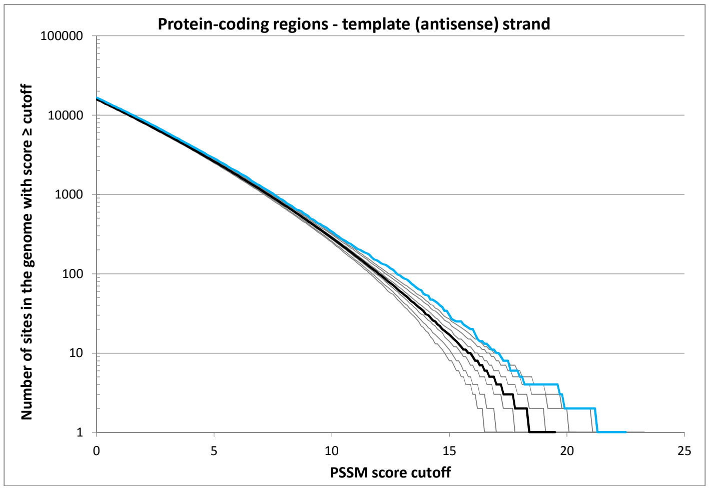

Test: *E. coli* LexA in *M. tuberculosis* genome

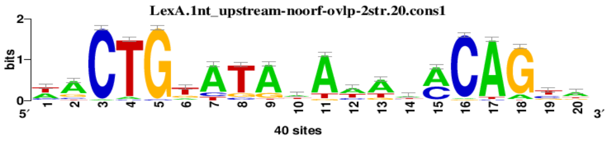

Motif length: 20
Motif information content: 17.93 bits
Max possible PSSM score: 34.66
Motif GC content: 36.12 %
Genome GC content: 65.61 %

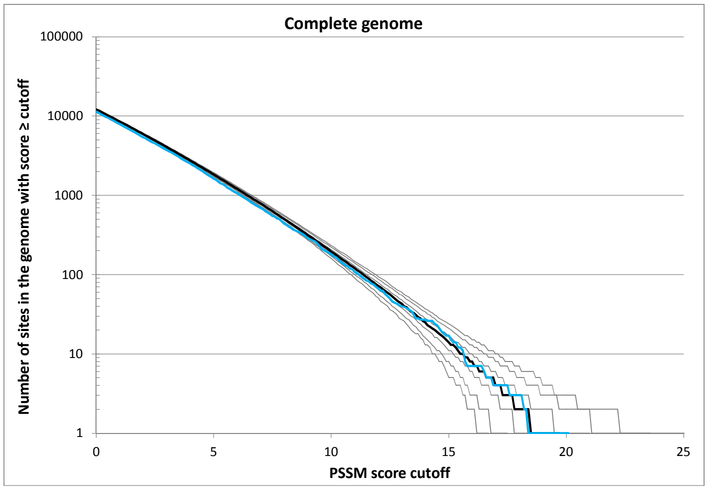

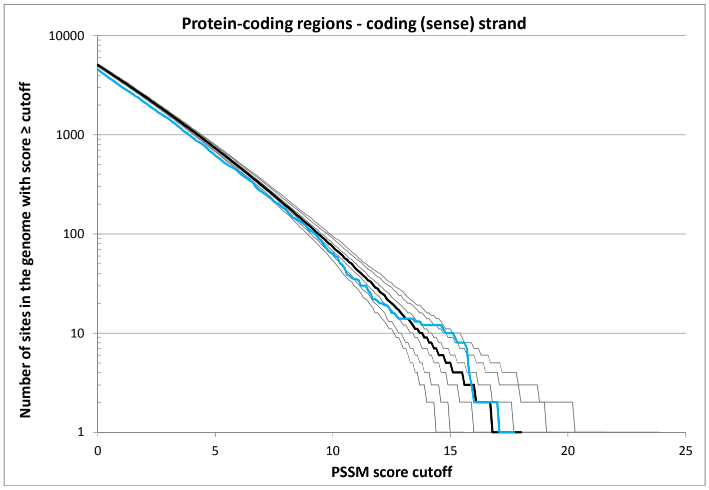

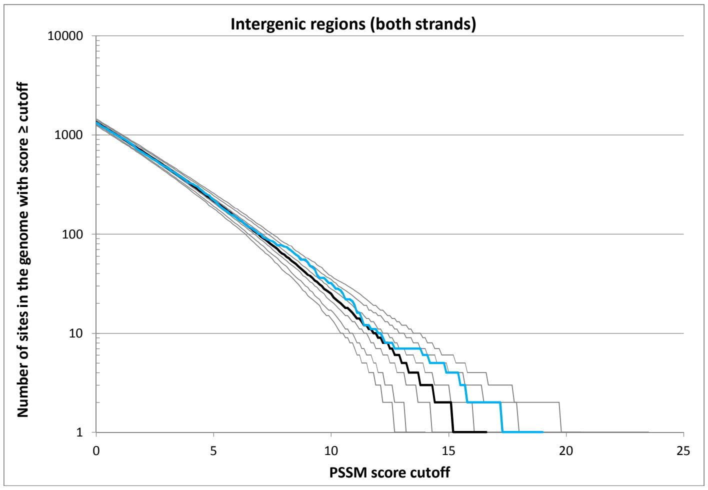

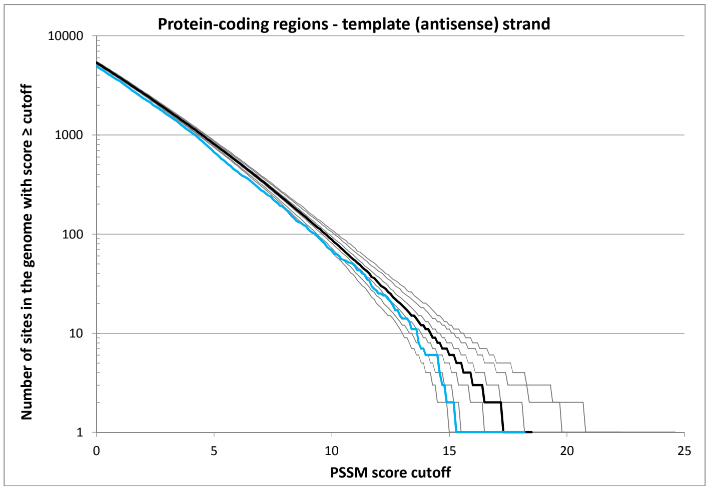

Test: *C. difficile* LexA in *E. coli* genome

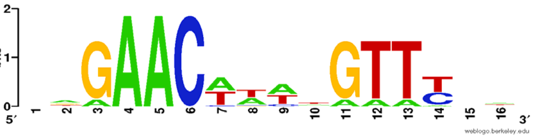

Motif length: 16
Motif information content: 15.44 bits
Max possible PSSM score: 20.16
Motif GC content: 31.62 %
Genome GC content: 50.79 %

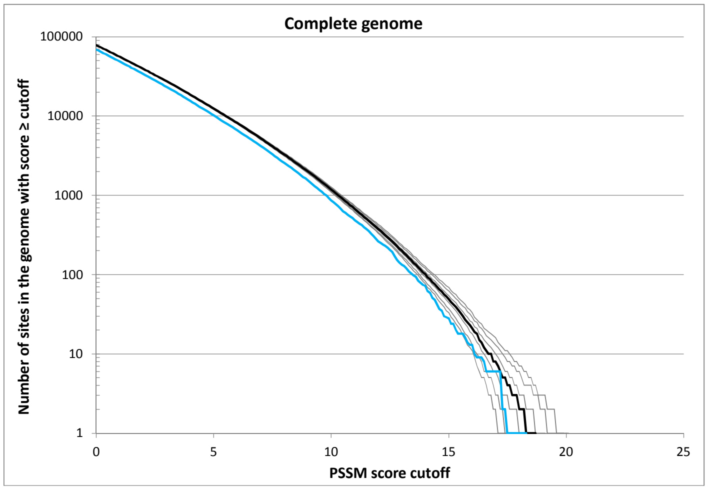

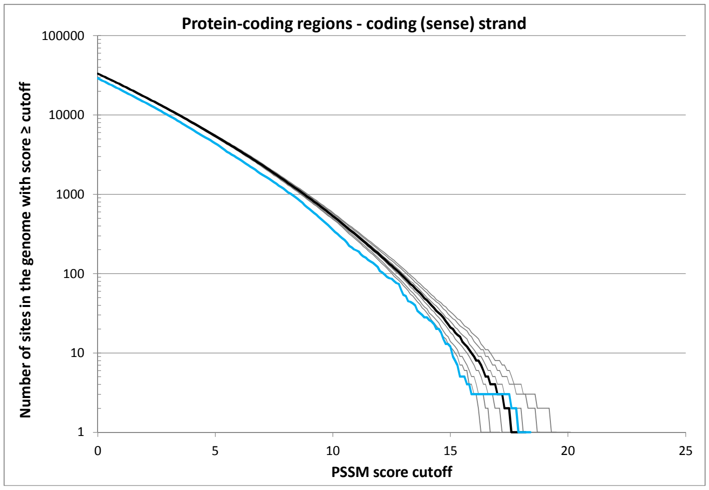

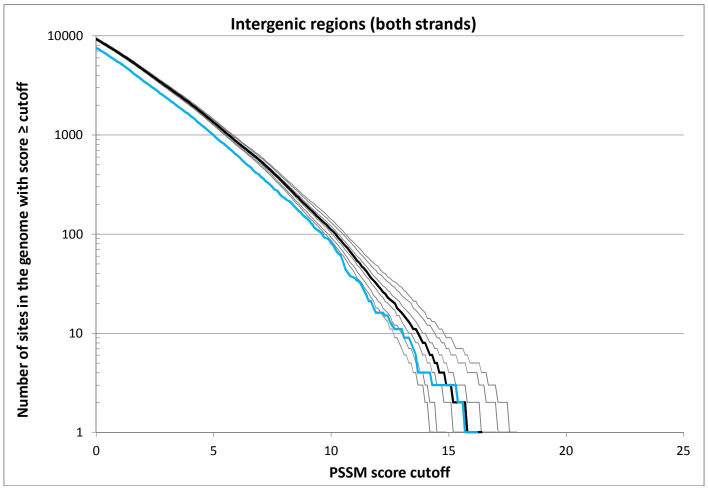

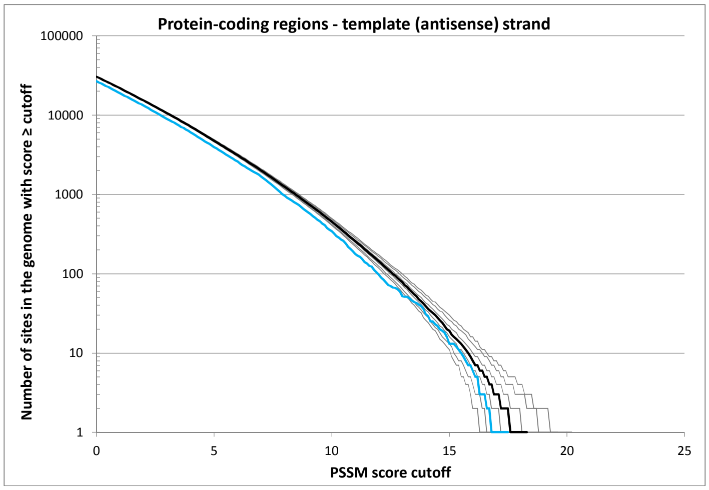

Test: *C. difficile* LexA in *M. tuberculosis* genome

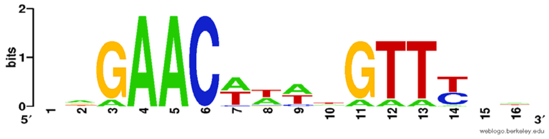

Motif length: 16
Motif information content: 15.44 bits
Max possible PSSM score: 25.64
Motif GC content: 31.62 %
Genome GC content: 65.61 %

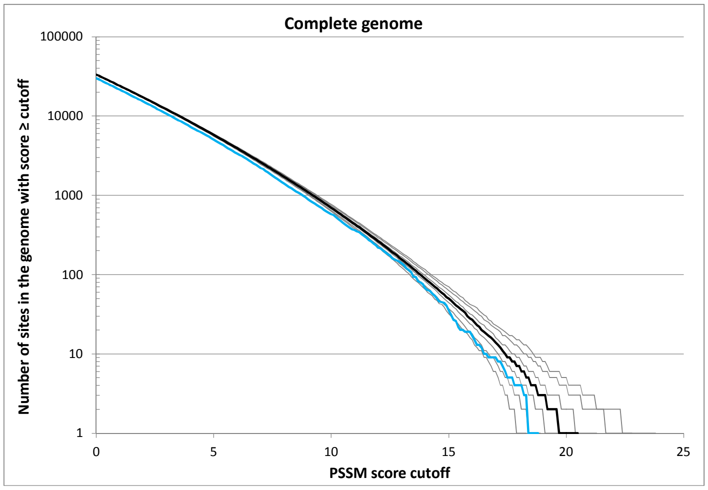

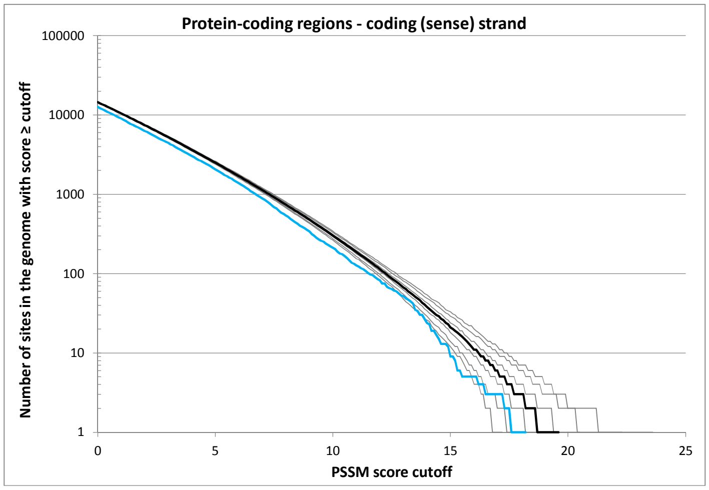

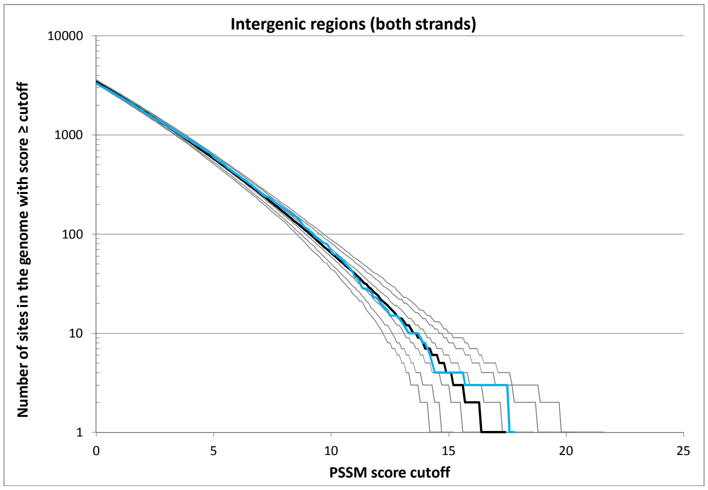

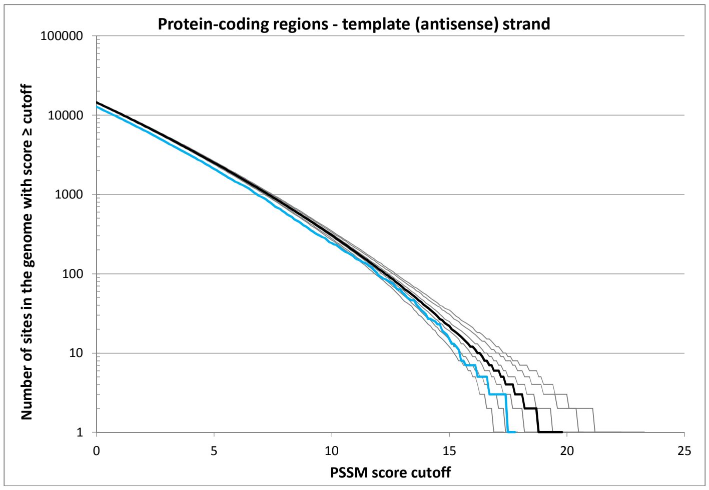

Test: *M. tuberculosis* LexA in *E. coli* genome

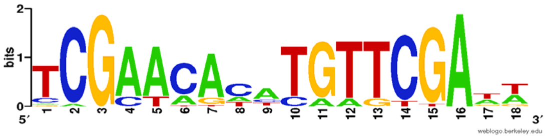

Motif length: 18
Motif information content: 22.54 bits
Max possible PSSM score: 28.80
Motif GC content: 40.58 %
Genome GC content: 50.79 %

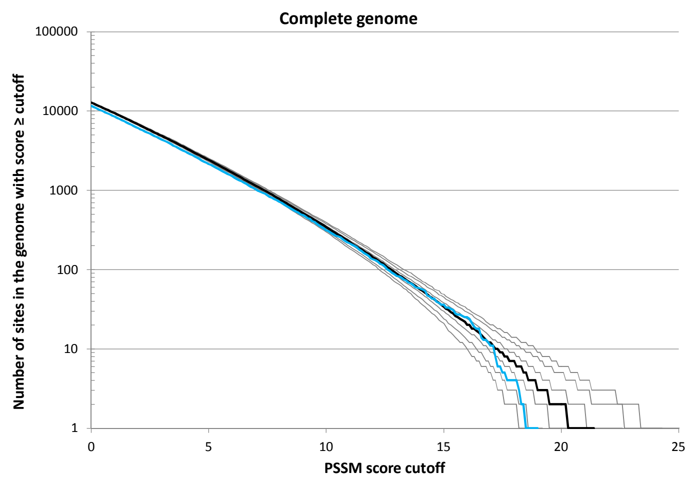

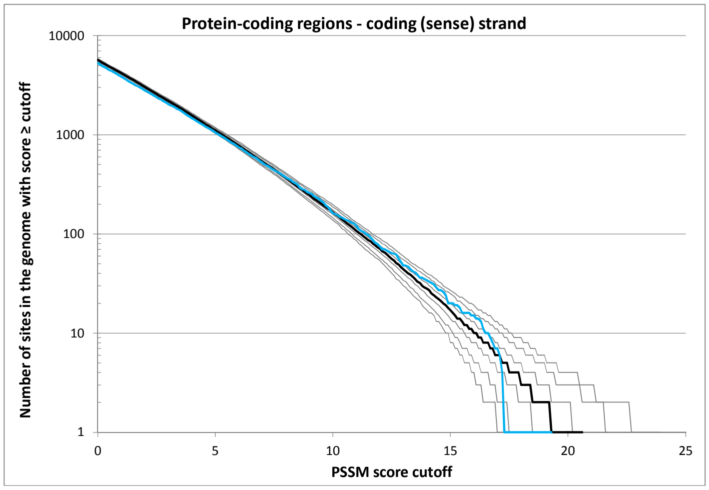

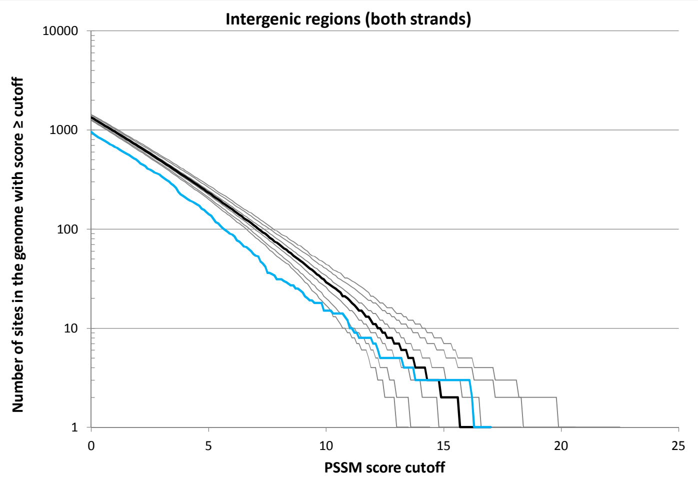

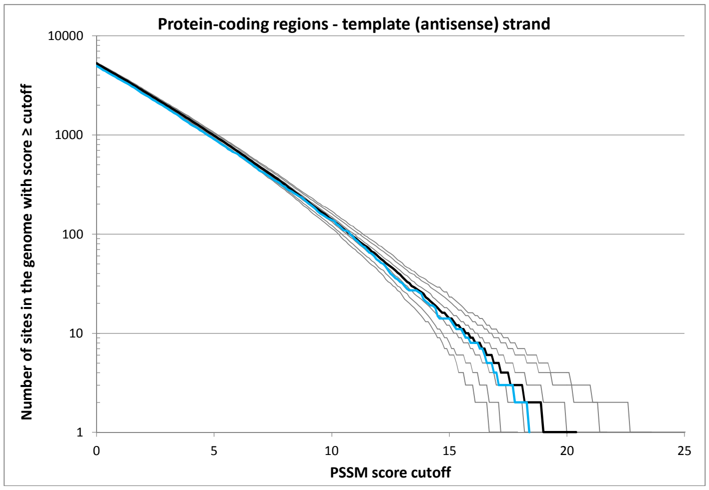

Test: *M. tuberculosis* LexA in *C. difficile* genome

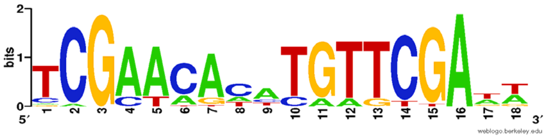

Motif length: 18
Motif information content: 22.54 bits
Max possible PSSM score: 28.74
Motif GC content: 40.58 %
Genome GC content: 28.81 %

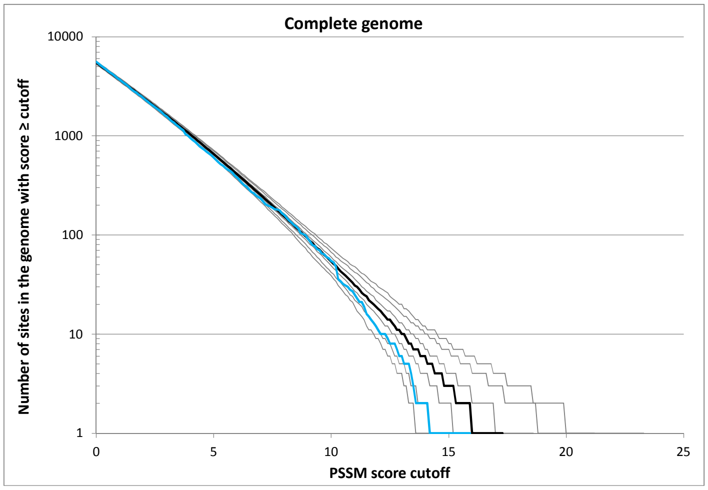

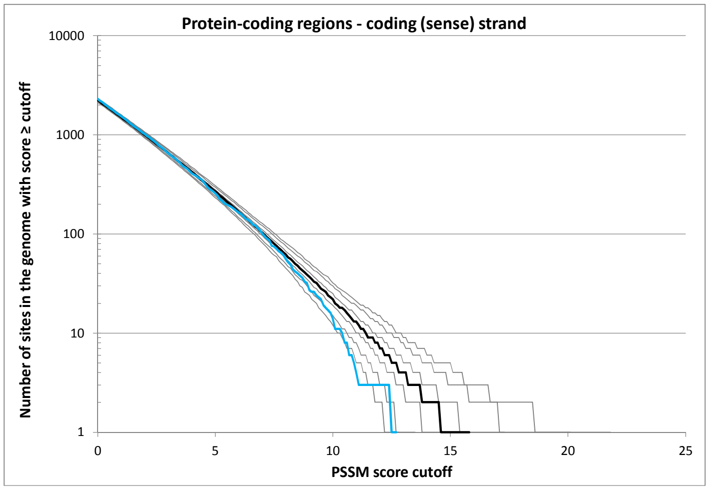

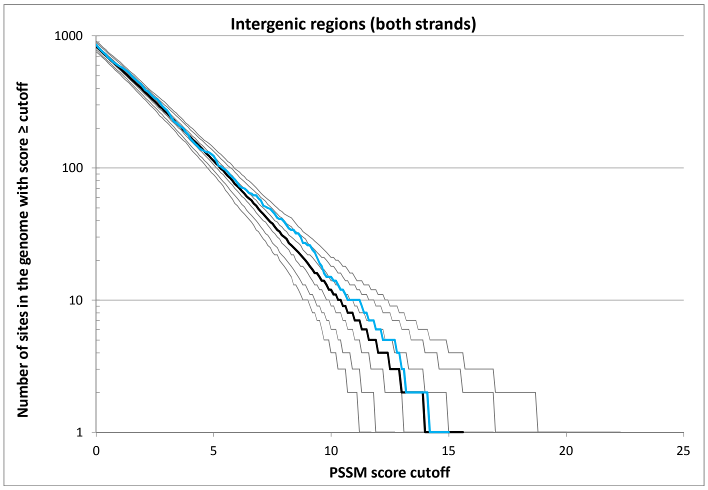

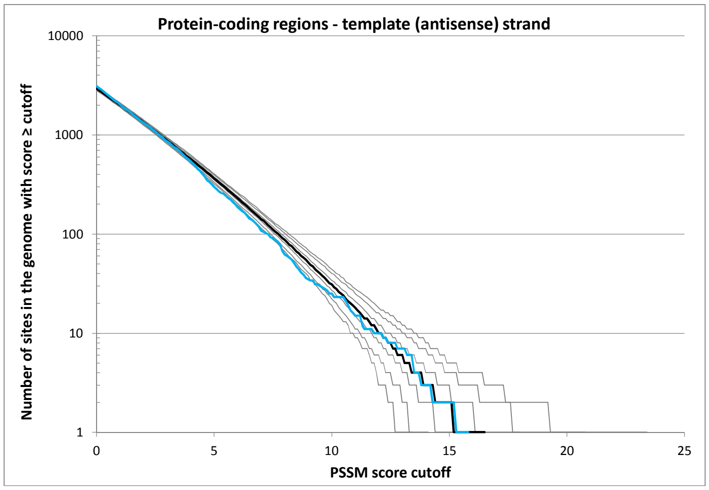

Test: *E. coli* FNR in *R. spharoides* genome

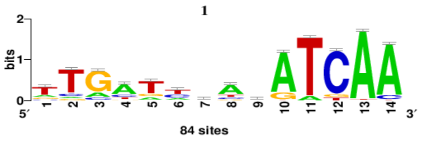

Motif length: 14
Motif information content: 11.06 bits
Max possible PSSM score: 26.26
Motif GC content: 30.10 %
Genome GC content: 69.01 %

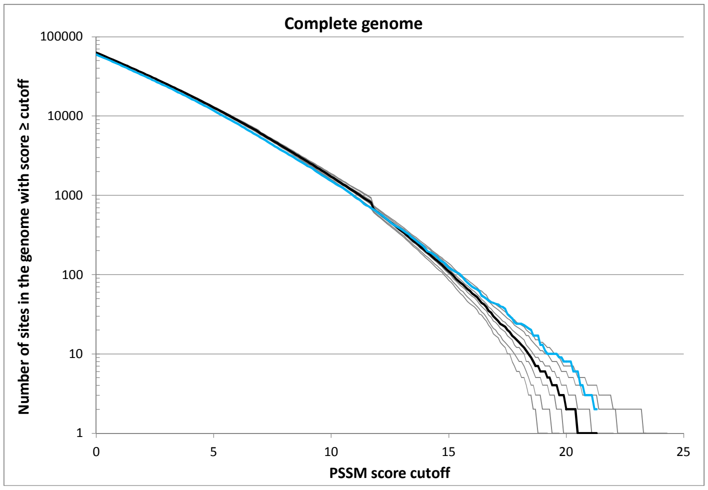

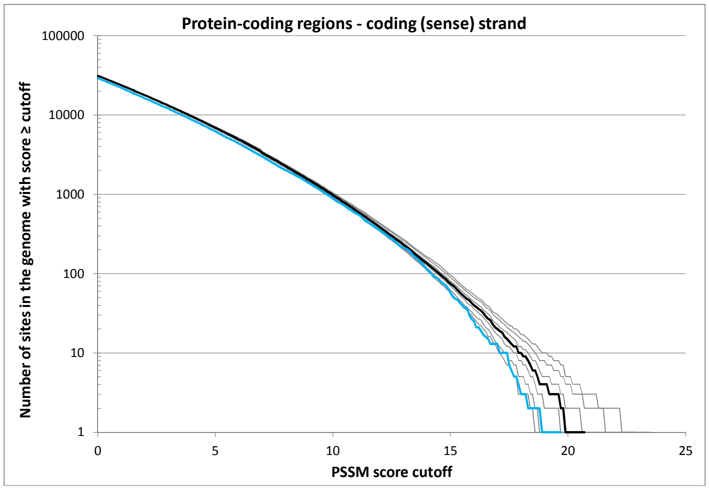

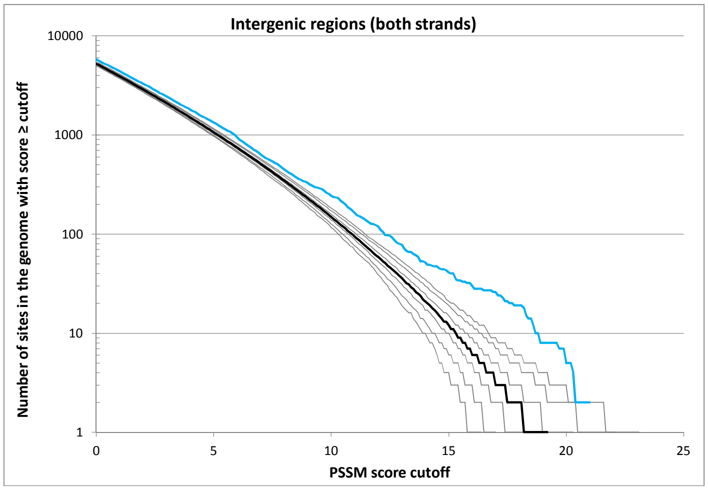

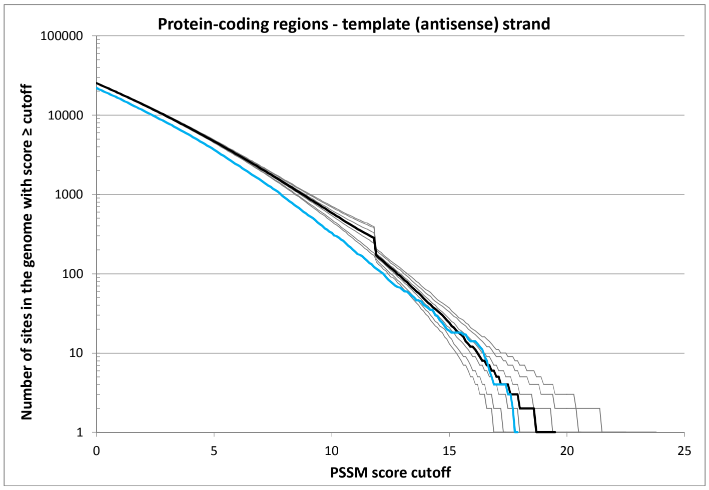

Test: *R. spharoides* FNR in *E. coli* genome

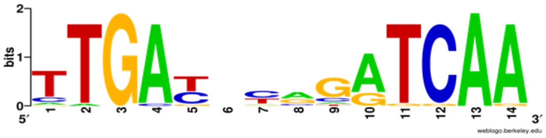

Motif length: 14
Motif information content: 17.13 bits
Max possible PSSM score: 21.49
Motif GC content: 39.95 %
Genome GC content: 50.79 %

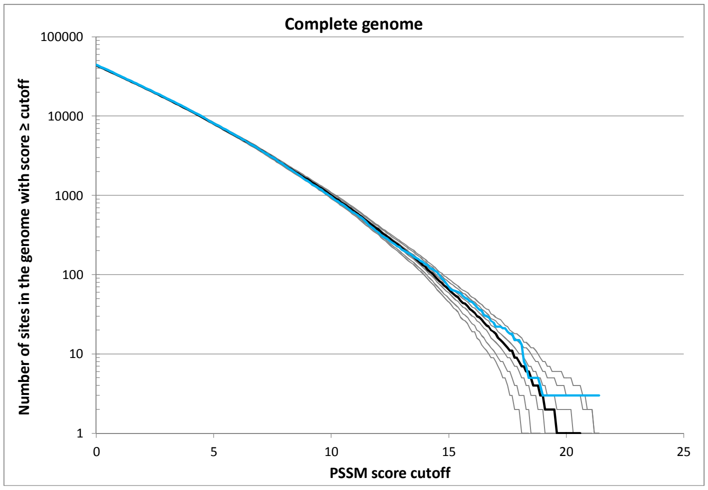

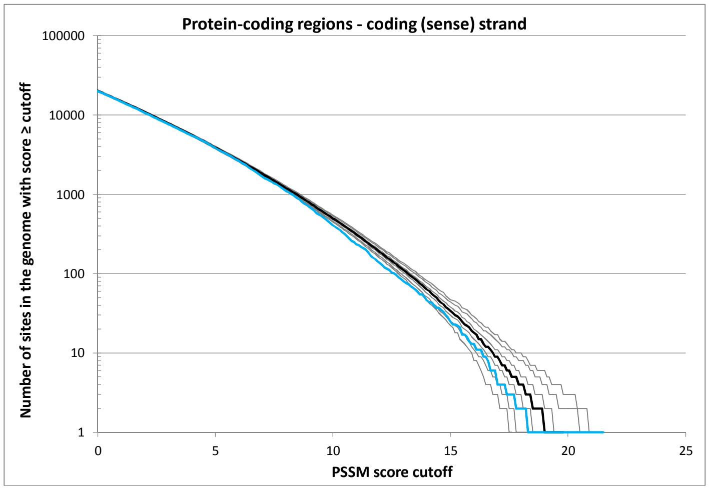

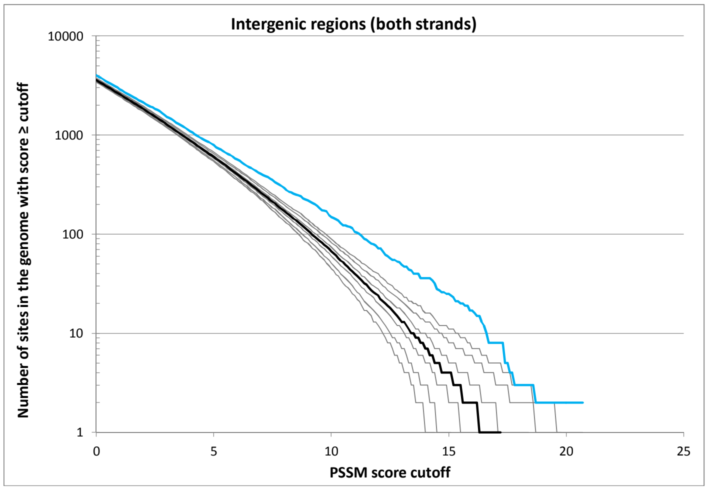

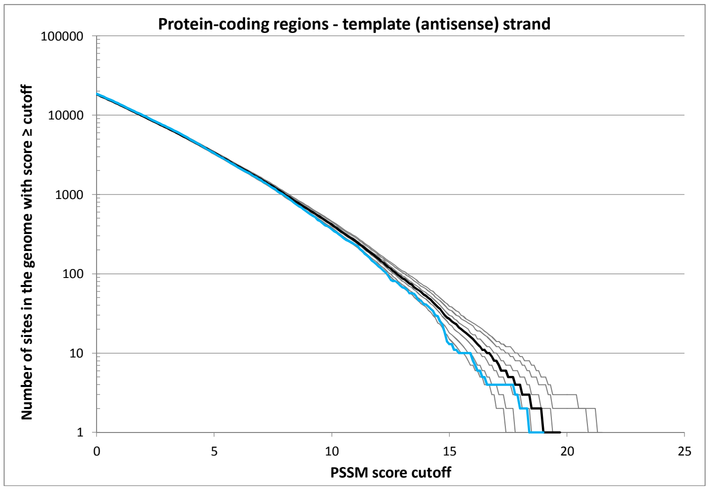

Test: FNR (*E. coli*) - only 20 sites most similar to the consensus

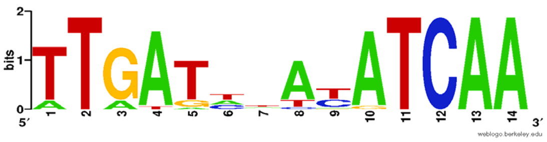

Motif length: 14
Motif information content: 19.61 bits
Max possible PSSM score: 22.68
Motif GC content: 22.14 %
Genome GC content: 50.79 %

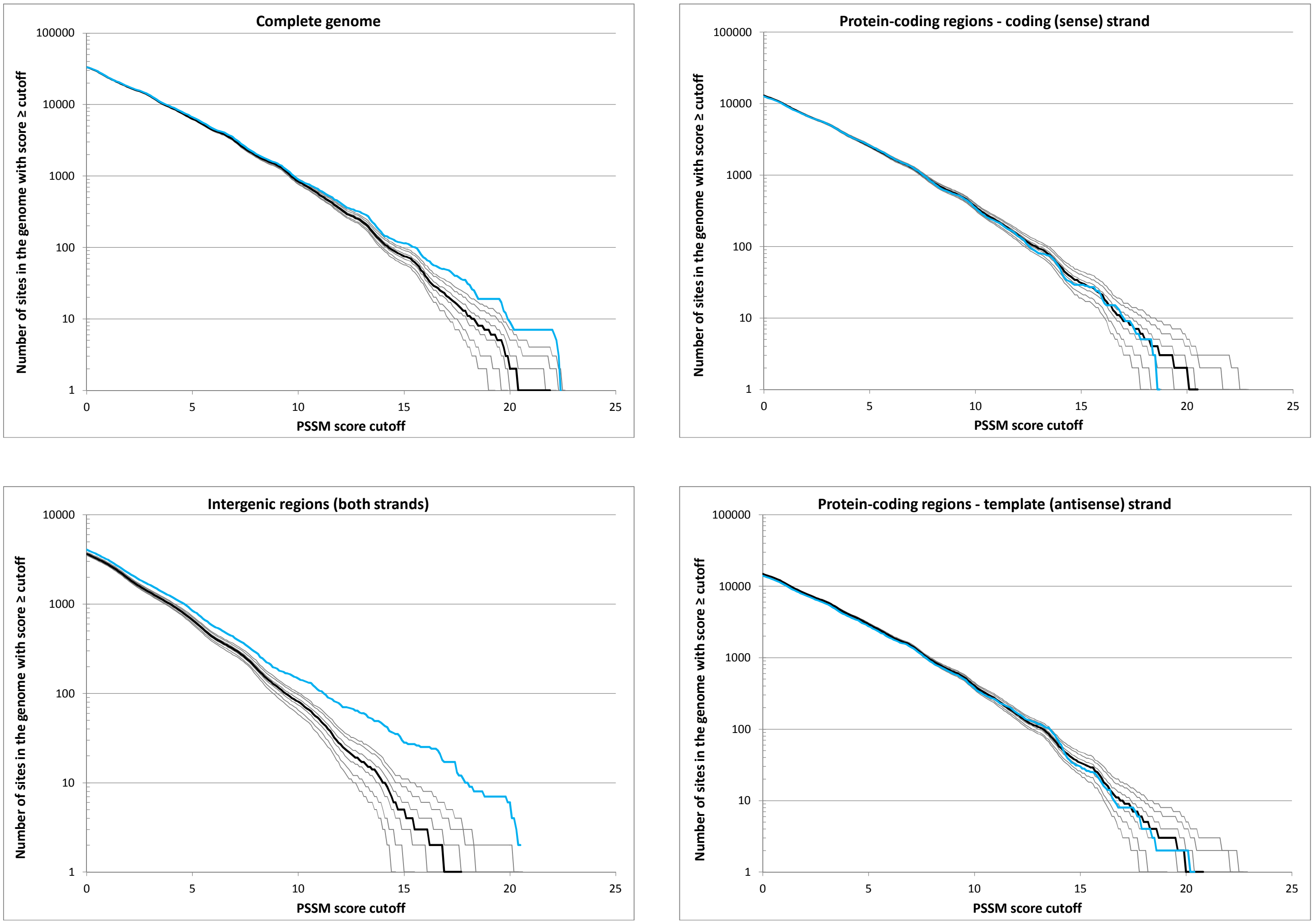

Supplement: Supplementary file 2 — Figure S2. ‘Report cards’ for additional tests including cross-species searches. (PDF 849 kb) [file 12862_2019_1381_MOESM2_ESM.pdf]
